# Cu(ii)-BODIPY photosensitizer for CAIX overexpressed cancer stem cell therapy†

**DOI:** 10.1039/d2sc03945a

**Published:** 2023-01-19

**Authors:** Hyo Sung Jung, Seyoung Koo, Miae Won, Seeun An, Haebeen Park, Jonathan L. Sessler, Jiyou Han, Jong Seung Kim

**Affiliations:** a Department of Biological Sciences, Hyupsung University Hwasung-si 18330 Korea hs0101j@gmail.com hanjiyou12@hanmail.net; b Department of Chemistry, Korea University Seoul 02841 Korea jongskim@korea.ac.kr; c Department of Chemistry, The University of Texas at Austin Austin Texas 78712-1224 USA sessler@cm.utexas.edu

## Abstract

Chemoresistance originating from cancer stem cells (CSCs) is a major cause of cancer treatment failure and highlights the need to develop CSC-targeting therapies. Although enormous progress in both photodynamic therapy (PDT) and chemodynamic therapy (CDT) has been made in recent decades, the efficacy of these modalities against CSC remains limited. Here, we report a new generation photosensitizer, CA9-BPS-Cu(ii), a system that combines three subunits within a single molecule, namely a copper catalyst for CDT, a boron dipyrromethene photosensitizer for PDT, and acetazolamide for CSC targeting *via* carbonic anhydrase-9 (CA9) binding. A therapeutic effect in MDA-MB-231 cells was observed that is ascribed to elevated oxidative stress mediated by a combined CDT/PDT effect, as well as through copper-catalysed glutathione oxidation. The CSC targeting ability of CA9-BPS-Cu(ii) was evident from the enhanced affinity of CA9-BPS-Cu(ii) towards CD133-positive MDA-MB-231 cells where CA9 is overexpressed *vs.* CD133-negative cells. Moreover, the efficacy of CA9-BPS-Cu(ii) was successfully demonstrated in a xenograft mouse tumour model.

## Abstract 2

Chemoresistance originating from cancer stem cells (CSCs) is a major cause of cancer treatment failure and highlights the need to develop CSC-targeting therapies.

## Graphical abstract

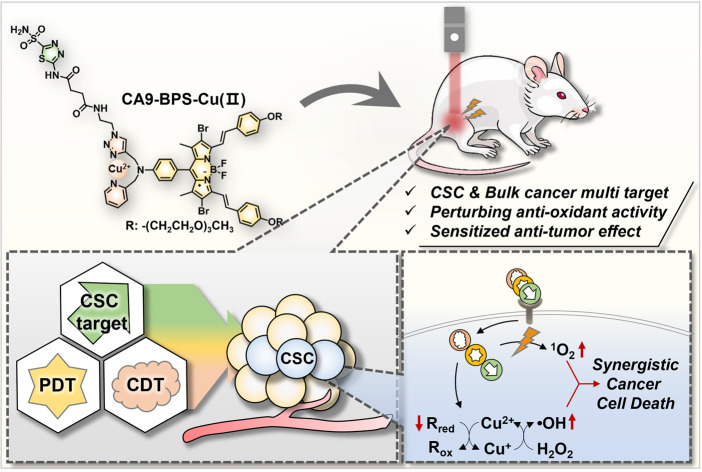

## Introduction

Cancer stem cells (CSCs) represent a small, highly tumourigenic subset of cancer cells recognized for their self-renewal capacity, multipotent differentiation, and propensity for sphere formation.^1^ CSCs have been found in most solid tumour types, including triple-negative breast cancer, and are linked to tumour recurrence and relapse following initial cancer treatment.^2^ Unfortunately, the current therapeutic approaches, including photodynamic therapy (PDT) and chemodynamic therapy (CDT), have little effect on CSCs. Advanced therapeutic strategies that could target and eradicate CSCs could improve the clinical outcomes by potentially reducing the risk of relapse after cancer treatment.

PDT is in clinical use for the treatment of skin cancer and several classes of subcutaneous tumours.^3^ The scope of PDT continues to expand and a broad range of deep tissue solid tumours, including breast cancers, are now being targeted through interstitial and intra-operative approaches.^4,5^ PDT typically relies on a combination of photo-irradiation and an appropriately chosen photosensitizer (PS) to produce singlet oxygen (^1^O_2_), a reactive oxygen species (ROS) that mediate cancer-killing effects, such as cell apoptosis, vascular degradation, and immune response.^6,7^ Ideally, the underlying photoactivation occurs with minimal side effects and high selectivity, thus endowing PDT with favourable safety profiles compared to conventional cancer therapies. In view of this, PDT has been considered a promising therapeutic option for tumours and a complement to other conventional therapies; however, the development of severe hypoxia within tumours significantly reduces the PDT outcome. PDT-induced hypoxia arises because of oxygen depletion directly through the photosensitization process or indirectly by vasculature damage and typically triggers the hypoxia inducible factor (HIF)-mediated signalling cascade.^8^ The resulting hypoxia and HIF signalling are considered potential contributors to CSC phenotypes,^9^ providing resistance to PDT.^10^ To date, enormous effort has been devoted to improving PDT, including selecting efficient PSs,^11^ effecting delivery to appropriate biological loci,^12^ regulating the reaction environments (*e.g.*, increasing effective O_2_ levels^13^ or decreasing cellular antioxidant levels^14^), and combining it with other modalities.^15^ However, there remains a need for more effective PDT methods and, in particular, developing CSC-targeted therapies remains a recognized therapeutic challenge.^16^

CDT is well-known for its unique ROS production pattern that is independent of local oxygen concentrations. In many cases, it relies on intracellular chemical reactions to decrease tumour vasculature or mediate an immune response.^17^ One key CDT reaction involves the conversion of hydrogen peroxide (H_2_O_2_) into the hydroxyl radical (·OH), which is a highly cytotoxic ROS capable of destroying cancer cells. Due to their abnormal metabolism, solid tumours are often characterized by high levels of H_2_O_2_ (100 μM to 1 mM), rendering this approach viable.^18,19^ Cu(i) ions are particularly effective redox-active catalysts that can trigger the formation of ·OH from H_2_O_2_*via* Cu(i)-catalysed Fenton-like reactions, including potentially in the acidic tumour microenvironment characteristic of many solid tumours. Cu(i) complexes are thus attractive for use in CDT.^20–22^ However, the presence of free Cu(i) ions can trigger adverse toxicity effects.^23,24^ Moreover, the cellular antioxidant system can limit the efficiency of CDT.^25^

Thus, it is important to target active Cu(i) ions in cancer cells and, if possible, harness endogenous reductants to promote conversion of H_2_O_2_ into ·OH. Several endogenous reductants, including non-enzymatic reductants (glutathione (GSH), cysteine (Cys), ascorbic acid (AA), *etc.*), and enzymatic reductants (GSH-S-transferase (GST), GSH-reductase (GR), catalase (CAT), superoxide dismutase (SOD), thioredoxin (Trx), *etc.*) are considered to be expressed at high levels in most cancer cells.^26^ Of these, GSH, the metabolism of which is enhanced in CSC-enriched culture, is arguably the best established biological reducing agent.^27^ Therefore, we sought to design a Cu(ii) ion-containing PS that would be stable in cellular environments and release a Cu(i) ion upon interaction with GSH and potentially other endogenous reductants, such as Cys and AA. This free Cu(i), in turn, would be expected to react with local H_2_O_2_ to produce toxic ·OH *via* a chemodynamic process. Since the concentrations of endogenous reductants and H_2_O_2_ are expected to be higher in cancer cells and CSCs, this approach might allow for a tumour-specific effect while also providing good cancer-killing efficiency.

The use of PDT, in combination with other treatment modalities, such as CDT, represents an attractive approach to improving therapeutic outcomes.^28^ To date, the combined effect of CDT and PDT has been shown to provide superior therapeutic effects in model studies, an observation that is rationalized in terms of the PDT-induced ROS acting as a substrate for CDT, thereby providing a synergistic effect.^29,30^ Several agents (*e.g.*, manganese silicate/calcium peroxide/indocyanine green nanoagents,^31^ copper ferrite nanoagents,^32^ porphyrin–ferrocene conjugates,^33^ ROS-activatable liposomes,^34^ copper/manganese silicate nanospheres,^35^*etc.*) that rely on different strategies, including GSH-depletion, hypoxia relief, and H_2_O_2_ supplementation, have been studied for improving therapeutic efficacy; however, to the best of our knowledge, the use of such strategies to target and eradicate CSCs has yet to be reported.

We felt that combining a PDT PS and Cu(i)-based CDT could be further enhanced by CSC targeting. Recently, a correlation between carbonic anhydrase IX (CA9) overexpression and CSCs, which mainly reside in hypoxic cancer cell niches, has been established.^36,37^ CA9 has emerged as a therapeutic target because of its role as a driver of ‘stemness’, including Notch1 and Jagged1 of CSCs, and in the expression of epithelial–mesenchymal transition (EMT) markers and regulators.^38^ Previous work has shown that targeting CA9 using an acetazolamide moiety can be useful in this regard.^39,40^ Recently, Lock *et al.* reported that inhibition of CA9 with CA9-specific inhibitors led to the depletion of CSCs within orthotopic breast tumour models.^41^ In particular, an enhanced therapeutic effect was observed *in vivo* in metastatic lung cancer mouse models after CA9 knockdown and treatment with the anticancer drug, paclitaxel.

**Scheme 1:**
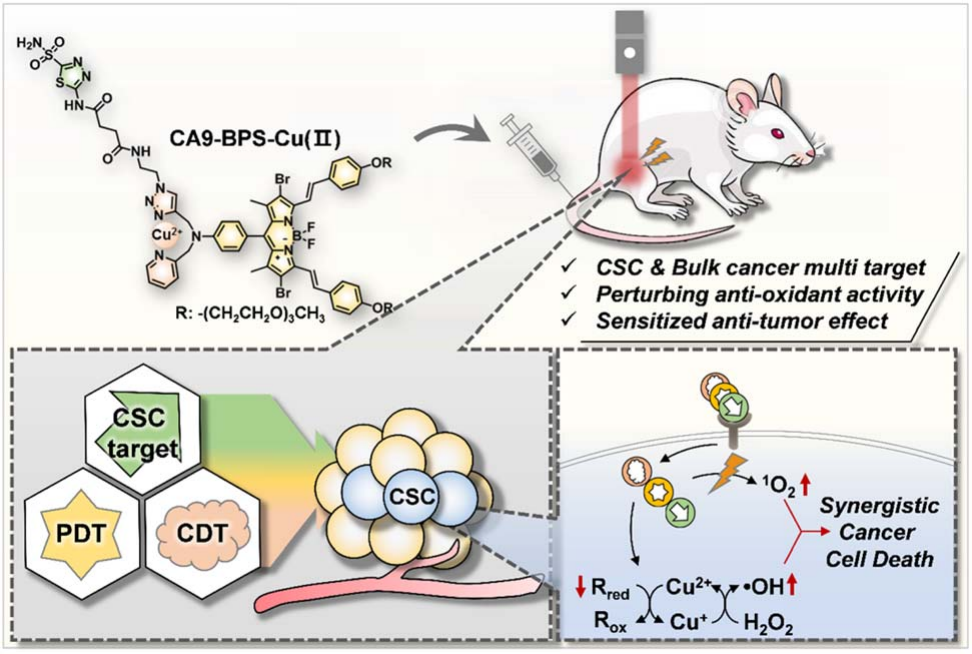
Schematic illustration of the synergistic anti-cancer effect expected to be produced by CA9-BPS-Cu(ii). R_red_, reduced form of endogenous reductants; R_ox_, oxidized form of endogenous reductants.

In this study we report a CA9-targeting copper–PS complex, CA9-BPS-Cu(ii), specifically designed to combine chemo- and photodynamic effects with an acetazolamide-based approach to CSC targeting (Scheme 1). Based on a combination of *in vitro* and *in vivo* studies, we found that the CA9-BPS-Cu(ii) system is effective at depleting CSCs in high CA9 breast cancer cells and retarding tumour growth under conditions of combined PDT and CDT. To the best of our knowledge, this is the first time these three disparate modalities (*i.e.*, CDT, PDT, and CSC targeting therapy) have been used as a single system to target and eradicate CSCs.

## Results and discussion

### Design and characterization of CA9-BPS and CA9-BPS-Cu(ii)

To design a putative anti-CSC sensitizer, an acetazolamide ligand was conjugated to a boron dipyrromethene (BODIPY) PS *via* a 2-picolyl-triazole copper-binding unit. BODIPY was chosen as the PS because of its good photo- and chemo-stability, high molar absorptivity, negligible photobleaching, and excellent bio-compatibility.^42^ The 2-picolyl-triazole linker was expected to coordinate Cu(ii) ions and release them as Cu(i) for CDT catalysis following endogenous reductant-mediated reduction and subsequent demetalation.^43^ With such considerations in mind, the metal-free form of the CA9-targeting BODIPY PS used in this study (CA9-BPS) was synthesized as shown in Fig. S1.† The analytical results (ESI-MS data, ^1^H NMR and ^13^C NMR spectra) for CA9-BPS and other new compounds were fully consistent with the proposed structures (*cf.* Fig. S34–S55†).

**Fig. 1:**
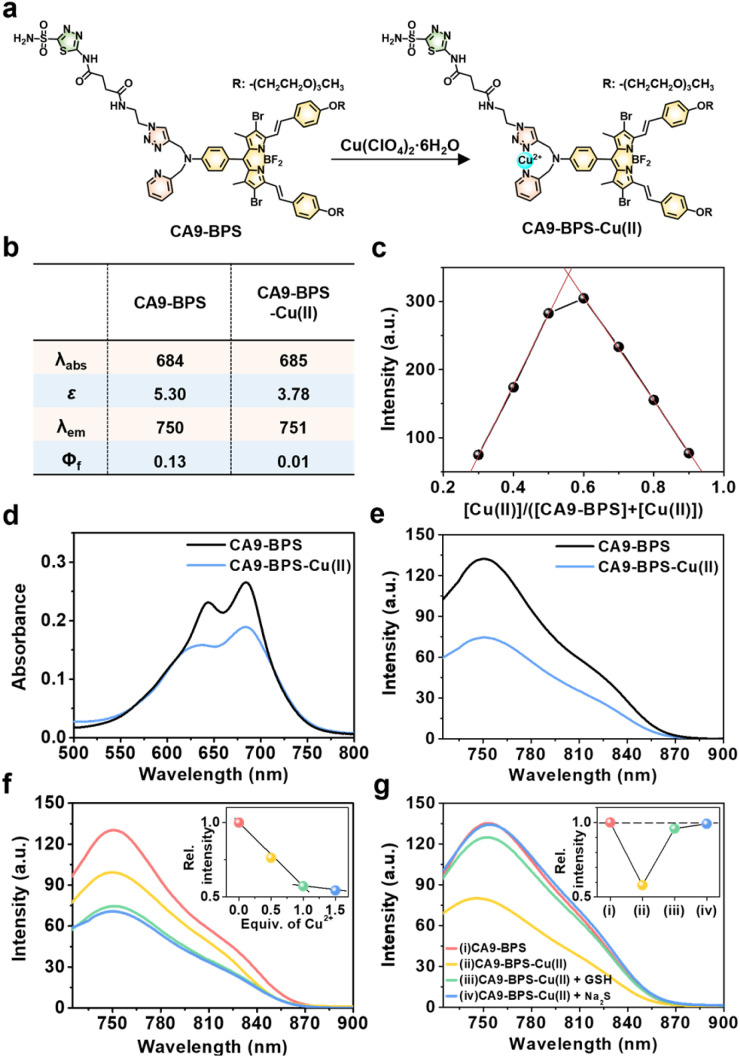
Effect of Cu(ii) complexation on CA9-BPSs. (a) Chemical structures of CA9-BPS-Cu(ii) and CA9-BPS. (b) Photophysical properties of CA9-BPS-Cu(ii) and CA9-BPS in PBS buffer solution (10 mM, pH 7.4, containing 5% DMSO). (c) Job's plot derived from the fluorescence changes seen for mixtures of CA9-BPS and Cu(ii). (d) Absorption and (e) fluorescence spectra of CA9-BPS-Cu(ii) and CA9-BPS (both at 5.0 μM) in PBS buffer solution (10 mM, pH 7.4, containing 5% DMSO). (f) Fluorescence spectra of CA9-BPS (5.0 μM) in PBS buffer solution (10 mM, pH 7.4, containing 5% DMSO) recorded at various relative concentrations of Cu(ii) (0–1.5 equiv.). Inset: plot of emission intensity at 750 nm *vs.* Cu(ii) equivalents. (g) Fluorescence spectra of CA9-BPS (red), CA9-BPS-Cu(ii) (yellow), CA9-BPS-Cu(ii) treated with 30 μM GSH (green), and CA9-BPS-Cu(ii) treated with 50 μM Na_2_S (blue) (5.0 μM CA9-BPSs in all cases) in PBS buffer solution (10 mM, pH 7.4, containing 5% DMSO). Excitation at 660 nm (slit = 20/20). The inset shows the relative change in intensity at 750 nm. *λ*_abs_: absorption maximum wavelength (nm). *ε*: Molar extinction coefficient (×10^4^ M^−1^ cm^−1^). *λ*_em_: emission maximum wavelength (nm). *Φ*_f_: fluorescence quantum yield.

Initial support for the expectation that CA9-BPS would support Cu(ii) complexation came from spectroscopic analyses. Addition of 1.0 equiv. of Cu(ii) (as the perchlorate salt) to CA9-BPS (both in ethanol solution) led to a 9 nm red shift in the absorption feature at 654 nm. The emission band of CA9-BPS at 680 nm was also substantially quenched, presumably due to a MLCT-based heavy-metal ion effect (Fig. 1a and S2†).^44^ The changes in the fluorescence intensity as a function of added Cu(ii) concentration could be fitted well to a 1 : 1 ligand : metal binding profile; however, poor fits were observed for a possible 2 : 1 complexation mode (*cf.* Fig. S3† for chemical structure of the proposed 2 : 1 complex).

Using a standard treatment as codified by Thordarson,^45^ the corresponding 1 : 1 binding constant was calculated to be (1.61 ± 0.07) × 10^5^ M^−1^ in ethanol. The MALDI-TOF/TOF-MS spectrum (Fig. S4†) and Job's plot analysis (Fig. 1c) of CA9-BPS treated with Cu(ii) also proved consistent with a 1 : 1 binding stoichiometry. Under conditions of PBS buffered solution (pH 7.4, 10 mM) containing 5% DMSO, the changes in the absorption and fluorescence spectra as a function of added Cu(ii) concentration were clearly observed (Fig. 1b and d–f). Importantly, the Cu(ii) complex of CA9-BPS was found to be stable over a period of 4 hours in PBS buffered solution (pH 7.4, 10 mM) containing 5% DMSO using HPLC analysis (Fig. S5†). However, the addition of 30 μM of GSH led to a significant recovery in the fluorescence intensity of CA9-BPS (Fig. 1g), which was taken as evidence of GSH-mediated loss of the bound Cu(ii) cation. The Cu(ii) complex used in this study (CA9-BPS-Cu(ii)) was purified using HPLC after adding 1.0 equiv. of Cu(ii) perchlorate to CA9-BPS. The integrity of CA9-BPS-Cu(ii) prepared in this way was confirmed using ESI-MS analyses (Fig. S55†).

### Photodynamic and chemodynamic properties of CA9-BPS-Cu(ii) and CA9-BPS

**Fig. 2:**
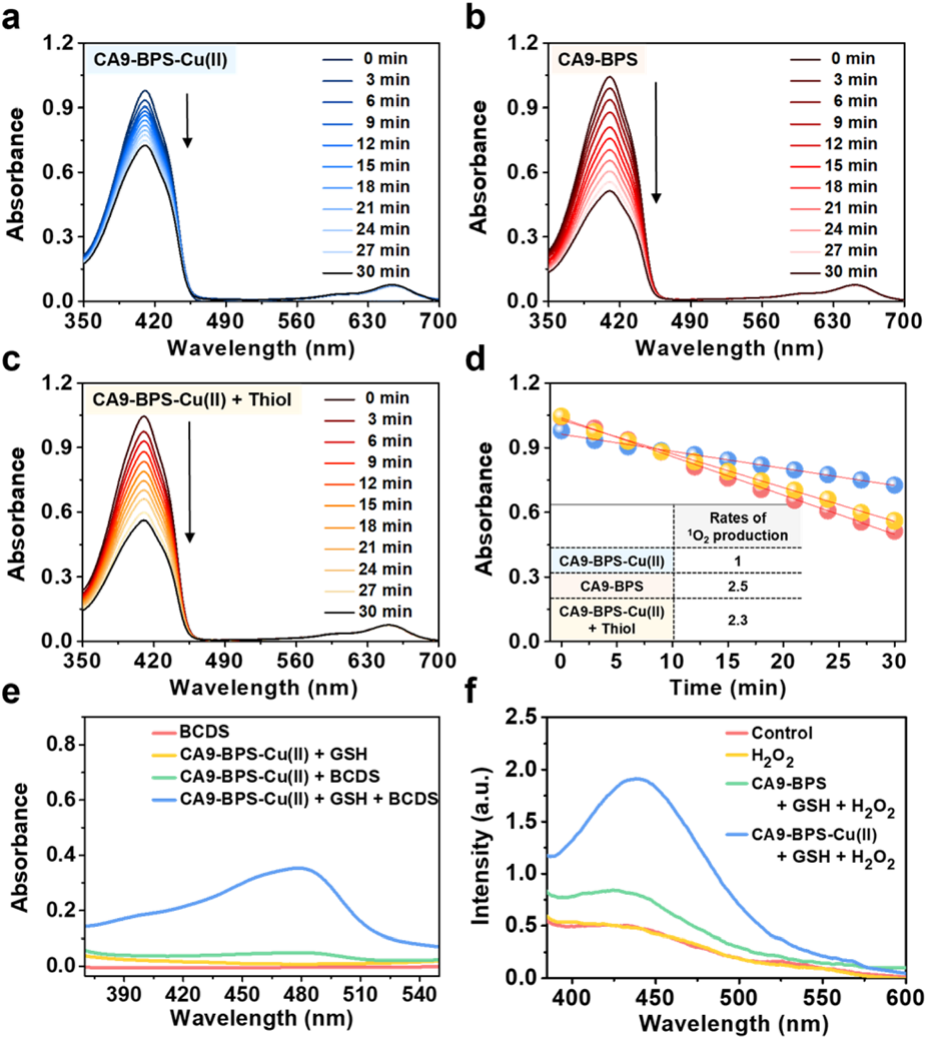
Photodynamic and chemodynamic properties of CA9-BPS-Cu(ii) and CA9-BPS. (a–d) Photosensitized ^1^O_2_ generation by CA9-BPS-Cu(ii) and CA9-BPS. Time-dependent absorption spectral changes seen for 80 μM solutions of 1,3-diphenylisobenzofuran (DPBF) containing 1 μM of (a) CA9-BPS-Cu(ii), (b) CA9-BPS or (c) CA9-BPS-Cu(ii) with added thiol mimic (1 equiv. Na_2_S); irradiation was effected at 660 nm (slit width = 15–1.5, Xe-lamp) in all three experiments. (d) Plots of the change in the absorption intensity at 412 nm for the experiments shown in (a–c). (e–h) Chemodynamic properties of CA9-BPS-Cu(ii). (e) Absorption spectra of bathocuproinedisulfonic acid disodium salt (BCDS), upon treatment with CA9-BPS-Cu(ii) in the presence and absence of GSH. (f) Emission spectra of terephthalic acid (TPA) upon treatment with CA9-BPS-Cu(ii) or CA9-BPS in the presence of other species, including GSH, and H_2_O_2_.

The ability of CA9-BPS-Cu(ii) and CA9-BPS to produce ^1^O_2_ under photo-irradiation was measured in acetonitrile solution using 1,3-diphenylisobenzofuran (DPBF) as a ^1^O_2_ indicator.^46^ Irradiation of CA9-BPS-Cu(ii) and CA9-BPS solutions, respectively, in the presence of DPBF with a 660 nm laser irradiation decreased the spectral absorption intensity ascribed to DPBF, as would be expected under conditions of ^1^O_2_ production (Fig. 2a and b). In contrast, no spectral changes were observed in the absence of photo-irradiation (Fig. S6†). In addition, negligible photothermal effects were seen for either CA9-BPS-Cu(ii) or CA9-BPS in biomimetic model systems, including MDA-MB-231 cell cytosol extract (20 μg mL^−1^), under conditions of high power density photo-irradiation (2.0 W cm^−2^, 10 min; 1200 J cm^−2^) (Fig. S7†). The rate of ^1^O_2_ production for the CA9-BPS-Cu(ii) solution was about 2.5 times smaller than that for CA9-BPS under these experimental conditions (Fig. 2d). Nevertheless, it was concluded that both systems act as PDT photosensitizers.

Furthermore, treating CA9-BPS-Cu(ii) with Na_2_S, a copper precipitant, increased the rate of ^1^O_2_ production to a similar level as that of CA9-BPS under identical conditions, which could be due to the release of the Cu(ii) cation triggered by Na_2_S, leading to regeneration of the more active metal-free form, CA9-BPS (Fig. 2c). On this basis we propose that the lower ^1^O_2_ production efficacy of CA9-BPS-Cu(ii) relative to CA9-BPS is due in large measure to quenching of the excited state by the Cu(ii) centre.

To probe whether CA9-BPS-Cu(ii) would act to release Cu(i) under reducing conditions, a mixed solution containing CA9-BPS-Cu(ii) and GSH was prepared. The addition of bathocuproinedisulfonic acid disodium salt (BCDS), a specific Cu(i) chelating agent,^47^ to this mixed solution gave rise to a new absorption band at 480 nm, which could be ascribed to the formation of the BCDS-Cu(i) complex (Fig. 2e). Furthermore, the addition of BCDS to CA9-BPS-Cu(ii) solutions containing the endogenous reductants Cys and AA showed similar absorption bands at 480 nm (Fig. S8†). In contrast, this band at 480 nm was not observed when a solution of CA9-BPS-Cu(ii) was tested in the absence of an endogenous reductant as a function of time (for 1 h to 24 h) (Fig. S9a and S9c†). Similar results were seen for CA9-BPS-Cu(ii) when the same experiment was performed using an RPMI cell culture media containing a diversity of biological species, including amino acids, inorganic salts, and vitamins (10% FBS, without phenol red) (Fig. S9b and S9d†). This difference was consistent with the design expectation, namely that cellular reductant-mediated reduction of CA9-BPS-Cu(ii) promotes the release of free Cu(i).

It has been shown that Cu(i)-catalysed Fenton-like reactions can efficiently produce ·OH in the presence of H_2_O_2_ in weakly acidic tumour microenvironments.^48^ To confirm that CA9-BPS-Cu(ii) could promote a Cu(i)-catalysed Fenton-like reaction, ·OH production by CA9-BPS-Cu(ii) was measured using terephthalic acid (TPA), a known ·OH trap.^49^ As shown in Fig. 2f, the addition of H_2_O_2_ to a mixed solution of CA9-BPS-Cu(ii) and GSH led to a dramatic enhancement in the TPA emission band at 440 nm after 1 h of incubation. In contrast, a negligible change was observed in a mixed copper-free solution consisting of CA9-BPS and GSH under identical experimental conditions. This was also true for solutions containing TPA only or TPA + H_2_O_2_. Taken together, these findings lead us to suggest that CA9-BPS-Cu(ii) could play a role as a Fenton reaction-assisted PDT sensitizer that is effective in tumour microenvironments.

### *In vitro* characterization of CA9-BPS-Cu(ii) as a putative anticancer sensitizer

Prior to the *in vitro* characterization of CA9-BPS-Cu(ii), the cellular expression of CA9 in various breast cancer cells (MDA-MB-231, MCF-7, T47D, SK-BR-3, BT-474, ZR-75-1, Hs578T, and MDA-MB-453) and non-malignant breast epithelial cells (MCF10A) was confirmed by western blot analysis. As shown in Fig. S10,† the expression of CA9 was considerably higher in MDA-MB-231 cells than in other breast cancer cells, including MCF-7. Expression of CA9 was also observed in the MDA-MB-453 cells under the identical experimental conditions. This finding is in agreement with a previous report^50^ and is considered supportive of the notion that CA9-targeting systems, such as CA9-BPS-Cu(ii), would display excellent CSC targeting affinity.^41^ Therefore, high CA9 MDA-MB-231 breast cancer cells were used to assess the therapeutic potential of CA9-BPS-Cu(ii).

**Fig. 3:**
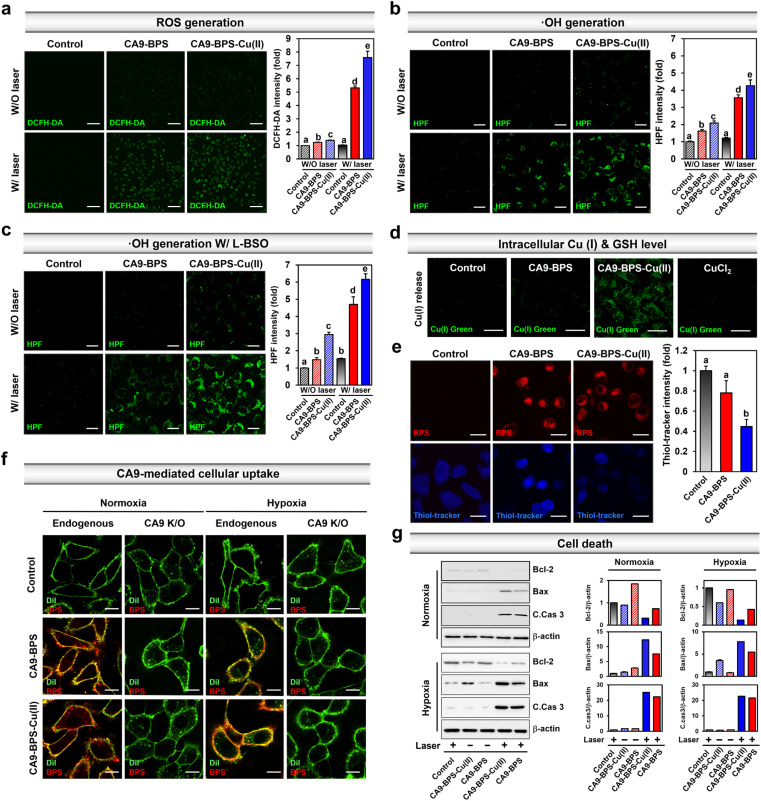
Mechanistic studies of CA9-BPS-Cu(ii) and CA9-BPS. (a) Confocal fluorescence microscopic images of MDA-MB-231 cells incubated with the two CA9-BPSs considered in the present study (5 μM, respectively) and 2′,7′-dichlorodihydrofluorescein diacetate (DCFH-DA; 10 μM) or (b and c) hydroxyphenyl fluorescein (HPF; 10 μM). The images were recorded with or without 660 nm laser irradiation (100 mW cm^−2^, 5 min; 30 J cm^−2^). For the experiment shown in (c), cells were subject to 100 μM l-buthionine sulfoximine (l-BSO) pretreatment 24 h before administering CA9-BPSs. Magnification & scale bars: (a) 20× &100 μm, (b and c) 90× & 20 μm. Histogram of the emission intensity was obtained by quantifying 5-region fields in cell images. (d) Confocal fluorescence microscopic images of MDA-MB-231 cells incubated with CA9-BPSs (5 μM) or CuCl_2_ (5 μM), respectively, with BioTracker GREEN Copper Dye. Magnification: 120×. Scale bars: 30 μm. (e) Confocal fluorescence microscopic images of MDA-MB-231 cells incubated with CA9-BPSs (5 μM) and with ThiolTracker®. Magnification: 120×. Scale bars: 20 μm. Histograms of the emission intensity for ThiolTracker® were generated by quantifying 5-region fields in the cell images. (f) Confocal fluorescence microscopic images of MDA-MB-231 cells (control or CA9 knockout cells) cultured under normoxic or hypoxic conditions and incubated with 5 μM CA9-BPSs (red) and 5 μM Dil (green; membrane staining dye) for 1 h. Magnification: 150×. Scale bars: 20 μm. (g) Western blot analysis of cell death markers in CA9-BPS-Cu(ii) or CA9-BPS-treated MDA-MB-231 cells which were cultured under normoxic or hypoxic conditions. A 660 nm LED lamp was used for photo-irradiation (100 mW cm^−2^, 3 min; 18 J cm^−2^). Data are presented as the mean, while the error bars indicate the standard deviation from the mean (*n* = 3). Statistical significance was determined by a two-way ANOVA test with a *post hoc* Bonferroni test. Different letters (a, b, c, d, e) in (a–c) and (e) signify datasets that are statistically distinct (*p* < 0.05).

To validate CA9-BPS-Cu(ii) as a putative anticancer sensitizer, intracellular ROS levels were measured in the MDA-MB-231 cell line using an intracellular ROS probe 2′,7′-dichlorodihydrofluorescein diacetate (DCFH-DA).^51^ Upon subjecting MDA-MB-231 cells incubated with CA9-BPS and CA9-BPS-Cu(ii) (5 μM, respectively) for 24 h to 660 nm photo-irradiation (100 mW cm^−2^, 5 min; 30 J cm^−2^), significant fluorescence enhancement of the DCFH-DA was observed for both CA9-BPS-Cu(ii) and CA9-BPS. Importantly, however, the fluorescence intensity in the CA9-BPS-Cu(ii)-treated group was about 1.4 times greater than that of the corresponding group treated with CA9-BPS (Fig. 3a).

Under identical photo-irradiation conditions, the CA9-BPS-Cu(ii)-mediated production of ^1^O_2_ in MDA-MB-231 cells was measured using singlet oxygen sensor green (SOSG) as a ^1^O_2_ probe.^52^ As shown in Fig. S11,† subjecting MDA-MB-231 cells incubated with CA9-BPS-Cu(ii) to photo-irradiation with 660 nm light led to significant fluorescence enhancement, as would be expected for ^1^O_2_ production under these conditions. A slightly weaker signal was observed for CA9-BPS under the same experimental conditions. In contrast, in the absence of photo-irradiation, very weak fluorescence intensities were observed for both CA9-BPS-Cu(ii) and CA9-BPS.

Next, the CA9-BPSs-mediated production of ·OH in MDA-MB-231 cells was measured using hydroxyphenyl fluorescein (HPF), a commercial ·OH probe.^53^ As shown in Fig. 3b, the addition of CA9-BPS-Cu(ii) to MDA-MB-231 cells containing HPF increased the fluorescence of HPF slightly relative to that of control. A fluorescence feature ascribable to HPF was observed for CA9-BPS-Cu(ii)-treated cells in the presence of photo-irradiation. Of note is that under identical photo-irradiation conditions, about 1.2 times greater ·OH production was observed in the case of CA9-BPS-Cu(ii)-treated cells than those treated with CA9-BPS (Fig. 3b). This finding can be construed as evidence for radical generation by this BODIPY PSs^54^ even if it is not potent as a singlet oxygen generator. The enhanced ·OH production in CA9-BPS-Cu(ii) is rationalized in terms of a combined CDT/PDT effect that serves to enhance the baseline PDT cytotoxicity.

As shown in Fig. 3c, pretreatment of l-buthionine sulfoximine (l-BSO), an inhibitor of *r*-glutamylcysteine synthetase, which reduces the levels of GSH,^55^ significantly enhanced the HPF fluorescence was seen in the cells incubated with CA9-BPS-Cu(ii) relative to those treated with CA9-BPS. These results could be ascribed to the critical role of GSH, which is an antioxidant recognized for its ability to neutralize intracellular ·OH.^56^ The CA9-BPSs-mediated ·OH production in normal BJ cells was negligible under identical experimental conditions as shown in Fig. S12.† As inferred from inductively coupled plasma-mass spectrometry (ICP-MS) analyses, the amount of copper uptake inside CA9-BPS-Cu(ii)-treated MDA-MB-231 cells was higher than in the control, CuCl_2_, and CA9-BPS-treated groups (Fig. S13†). In addition, a significant increase in Cu(i) fluorescence signals and decrease in intracellular GSH levels was observed in the CA9-BPS-Cu(ii)-treated cell group (Fig. 3d, e and S14†).

Based on the results in Fig. 3, we conclude that the addition of CA9-BPS-Cu(ii) to cancer cells followed by reaction with endogenous reductants, promotes demetallation and release Cu(i). The generated Cu(i) then produces ·OH in the tumour microenvironment. CA9-BPS-Cu(ii) is also expected to reduce the GSH levels *via* oxidation of GSH to GSSG. The corresponding reduction in GSH levels would, in turn, serve to enhance the cytotoxic effects arising from ·OH generation by CA9-BPS-Cu(ii). The net result would be a synergistic effect that abets cell apoptosis by increasing the ROS levels within the cancer cell.

The CA9-dependent cellular uptake of CA9-BPSs was studied using CA9-knockout MDA-MB-231 cell lines established by CA9 CRISPR-Cas9 transfection (Fig. S15†). After 1 h incubation with the CA9-BPSs in MDA-MB-231 cells, bright red fluorescence signals attributed to CA9-BPSs were observed, while negligible fluorescence signals corresponding to the CA9-BPSs were seen in the CA9-knockout MDA-MB-231 cells; this was true under both normoxic and hypoxic conditions (Fig. 3f and S16†). Notably, the fluorescence signals of the CA9-BPSs were more densely localized on the cellular membrane when the cells were cultured under hypoxic conditions, presumably due to the upregulated expression of membrane-bound CA9 proteins (Fig. S16†). Meanwhile, as the incubation time was extended to 24 h, CA9-BPSs were seen distributed throughout the cell compartment, rather than within specific cell organelles (Fig. S17†). Western blot analyses were then carried out in an effort to probe ROS-mediated cell death mechanisms.^57^ In the absence of light irradiation, MDA-MB-231 cells treated with CA9-BPS or CA9-BPS-Cu(ii) did not show significant changes in apoptosis-related markers. On the other hand, after photo-irradiation, CA9-BPS-Cu(ii)-treated MDA-MB-231 cells showed an enhanced expression of pro-apoptotic proteins (Bax, C.Cas 3; cleaved caspase 3) and reduced expression of anti-apoptotic protein (Bcl-2) under both normoxic and hypoxic conditions, compared to what was seen in the case of the CA9-BPS-treated cells (Fig. 3g). These results provide support for the core suggestion that CDT/PDT processes mediated by CA9-BPS-Cu(ii) are effective in promoting cancer cell death.

### Cytotoxicity of CA9-BPS-Cu(ii) and CA9-BPS

**Fig. 4:**
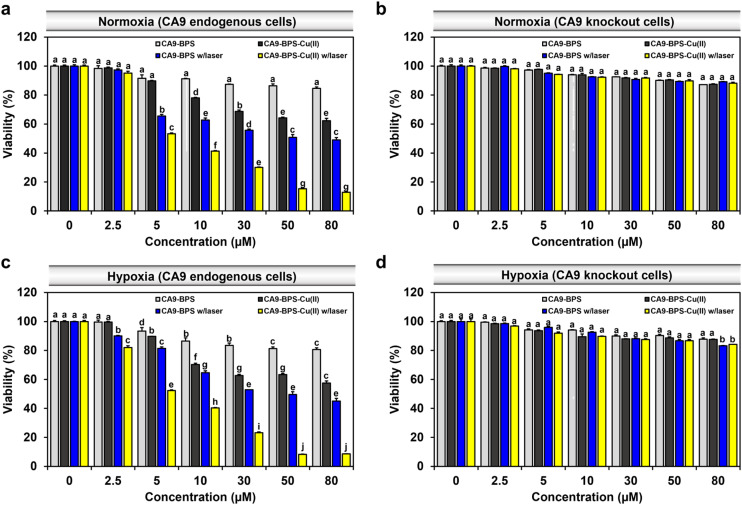
Cytotoxicity of CA9-BPS-Cu(ii) and CA9-BPS. *In vitro* cytotoxicity of CA9-BPS-Cu(ii) and CA9-BPS as tested in (a) normoxic MDA-MB-231, (b) normoxic CA9 knockout MDA-MB-231, (c) hypoxic MDA-MB-231, and (d) hypoxic CA9 knockout MDA-MB-231 cells treated with various concentrations (2.5 to 80 μM) of CA9-BPS-Cu(ii) or CA9-BPS and 1% DMSO (as a control) for 24 h. Then, the cells were photo-irradiated with a 660 nm LED lamp (100 mW cm^−2^, 5 min; 30 J cm^−2^). WST-8 assays were performed 24 h after irradiation. Data are presented as the mean, while the error bars indicate the standard deviation from the means (*n* = 3). Statistical significance was determined using a two-way ANOVA test with a *post hoc* Bonferroni test. Different letters (a–j) signify statistically distinct datasets (*p* < 0.05) in (a–d).

To compare the relative *in vitro* cytotoxicity of CA9-BPS-Cu(ii) and CA9-BPS, a cell viability assay was conducted with MDA-MB-231 cells. As shown in Fig. 4a, the viability of the MDA-MB-231 cells treated with 30 μM CA9-BPS-Cu(ii) was reduced (∼31%), whereas less reduction was observed in CA9-BPS-treated cells under identical experimental conditions. In contrast, the toxicity effects of CA9-BPS-Cu(ii) or CA9-BPS at a concentration of <80 μM were negligible in normal human fibroblasts BJ cells (Fig. S18†). From these results, it is evident that CA9-BPS-Cu(ii) stimulates a CDT effect in MDA-MB-231 cells but produces little damage to BJ cells at the test concentrations. The minimal damage seen for the BJ cells is ascribed to the extremely low concentration of CA9, as well as the low H_2_O_2_ activity, characteristic of this normal cell line.^12,58^

To assess the benefit of the putative combined CDT/PDT effect, MDA-MB-231 cells were tested with CA9-BPS-Cu(ii) in the presence and absence of laser irradiation. Under conditions of photo-irradiation (660 nm, 100 mW cm^−2^, 5 min; 30 J cm^−2^), the cytotoxic effect of CA9-BPS-Cu(ii) was found to increase in a dose-dependent fashion. At equal concentrations (30 μM) and otherwise identical conditions, CA9-BPS-Cu(ii) reduced the cell viability more effectively than CA9-BPS (∼70% *vs.* ∼44%) (Fig. 4a). Furthermore, the combination index (CI) value for treatment by CDT and PDT proved consistent with a strong synergistic cytotoxic effect (CI < 1) in MDA-MB-231 cells (Fig. S19†).

The synergistic cytotoxic efficacy seen for CA9-BPS-Cu(ii) is thought to reflect the ancillary therapeutic benefit of Cu(i)-mediated production of ROS, a CDT effect that might be enhanced, in part, by copper-catalysed GSH depletion. Support for this latter proposition came from the finding that when the cells were pretreated with *N*-ethylmaleimide (NEM),^59^ a GSH inhibitor, enhanced photocytotoxicity was observed for CA9-BPS-Cu(ii) at low concentrations (<10 μM) (Fig. S20†). This finding is consistent with the elevated levels of oxidative stress expected for cancer cells subjected to GSH depletion.

Contrary to the superior cell death seen for the CA9-BPS-Cu(ii)-treated CA9-high MDA-MB-231 cells, negligible cytotoxicity was observed in CA9-low MCF-7 cells (Fig. S21†). As shown in Fig. S22,† confocal fluorescence imaging with propidium iodide (PI) staining also revealed disparities in the photo-induced cytotoxicity between these two breast cancer cell lines. Moreover, the cytotoxicity of CA9-BPS-Cu(ii) was dramatically suppressed in CA9-knockout MDA-MB-231 cells and in MDA-MB-231 cells pretreated with acetazolamide as a presumed antagonist of CA9 (Fig. 4b and S23†). This finding is consistent with the proposed CA9 specificity of CA9-BPS-Cu(ii), which, per our molecular design, is thought to arise from the conjugated acetazolamide ligand. Based on these results, we considered that CA9-BPS-Cu(ii) would be effective as a photo-mediated cancer treatment for tumours characterized by high levels of CA9.

Hypoxia stabilizes the HIF1-alpha mediated signalling cascade, which confers resistance in cancer cells to conventional therapies, by reprogramming cell metabolism, inhibiting cell death signalling, and maintaining cancer stemness.^60,61^ CA9 expression is positively regulated by HIF1-alpha, thus CA9-targeted therapy constitutes a promising strategy for hypoxic tumour therapy.^62^ In accord with such expectations, we found that our CA9-BPSs give rise to a considerable anti-cancer effect even in hypoxic MDA-MB-231 cells (Fig. 4c). Based on an appreciation that (1) the cytotoxicity of CA9-BPSs is highly dependent on CA9 expression levels (*cf.*Fig. 4a and b) and (2) endogenous CA9 levels are increased under hypoxic conditions in MDA-MB-231 cells (Fig. S24†),^36^ we propose that both the enhanced cellular uptake of our CA9-BPS systems in hypoxic MDA-MB-231 cells and an inhibition of the CA9 signalling cascade acts to mitigate to some extent the therapeutic resistance of hypoxic MDA-MB-231 cancer cells. Interestingly, in our experimental setting, maintaining hypoxic conditions did not upregulate CA9 expression in MCF-7 cells,^36^ thus leading to no noticeable increase in CA9-BPSs cytotoxicity (Fig. S21†).

Acetazolamide is known as a non-specific CA inhibitor. Appreciating this and with a desire to probe the putative correlation between our systems and CAs, including CA12, we performed *in vitro* cytotoxicity studies using CA12-low T47D and CA12-high BT-474 breast cancer cells (Fig. S25 and S26†). As shown in Fig. S26,† the slightly stronger photo-induced cytotoxicity effects of CA9-BPS-Cu(ii) were observed for the CA12-high BT-474 breast cancer cells compared to CA12-low T47D cancer cells, while in both cases the effects were weaker than the case seen for the CA9-high MDA-MB-231 breast cancer cells (Fig. 4a). Therefore, we speculate that our systems might also have a slight effect on CA12. However, the effect on CA9 is clear. In this context, we note that several CA9 isoform-specific inhibitors have now been described and are in clinical trials.^63^ This foundational progress could set the stage for the development of improved multimodal systems based on, *e.g.*, CSC targeting.

### CSC targeting by CA9-BPS-Cu(ii)

**Fig. 5:**
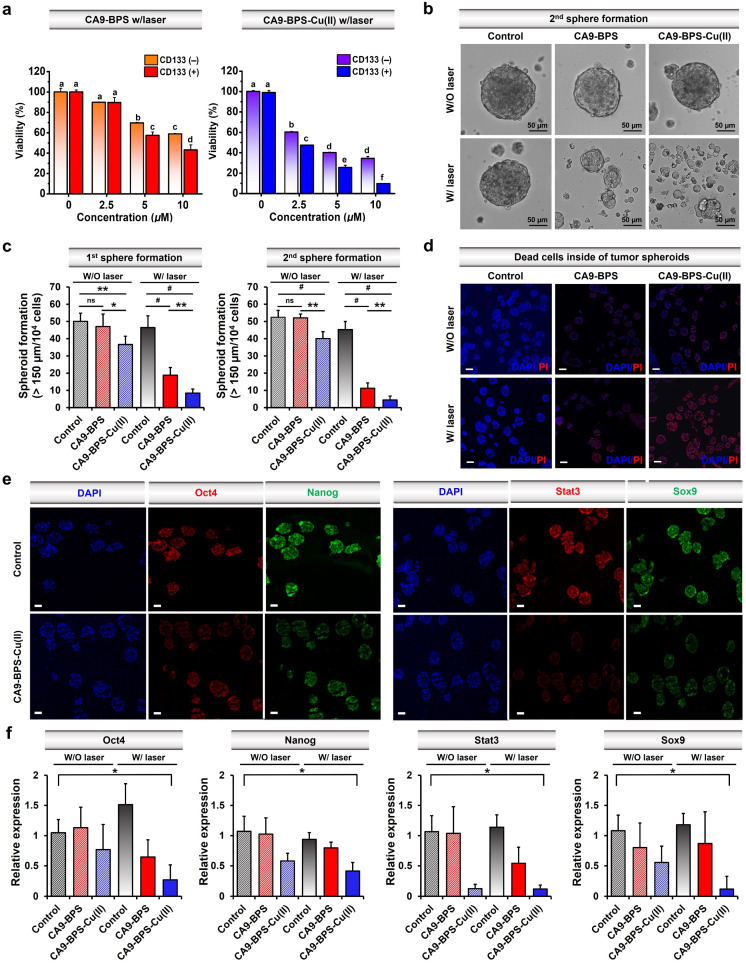
Therapeutic effects of CA9-BPS-Cu(ii) and CA9-BPS on CSCs. (a) Cytotoxicity of CA9-BPS-Cu(ii) and CA9-BPS toward CD133-positive and CD133-negative MDA-MB-231 cells obtained by magnetic-activated cell sorting (MACS). The sorted cells were seeded 1.0 × 10^4^ per each well in a 96-well plate for WST-8 assay. (b) Images showing secondary tumour spheroid formation in MDA-MB-231 cells treated with CA9-BPS-Cu(ii) or CA9-BPS (5 μM, respectively), or with 1% DMSO (as a control) with or without laser irradiation (660 nm; 100 mW cm^−2^; 5 min; 30 J cm^−2^) were recorded for 5 days. Magnification: 20×. Scale bars: 50 μm. (c) Total number of tumour spheroids found in both primary and subsequent secondary sphere-formation assays. (d) DAPI (blue) and PI (red) staining for dead cells inside of tumour spheroids formed by CD133-positive cells. CA9-BPS-Cu(ii) or CA9-BPS (5 μM) was treated to tumour spheroids on the 2nd day and irradiated with a 660 nm lamp for 5 min on the 3rd day. Magnification: 10×. Scale bars: 100 μm. (e) Immunocytochemistry of tumour spheroids. The tumour spheroids were cultured and treated with 5 μM CA9-BPS-Cu(ii) under the same conditions as in (d). Magnification: 10×. Scale bars: 100 μm (f) Gene expression of stemness-related genes by real-time PCR. Data are presented as the mean, while the error bars indicate the standard deviation from the mean (*n* = 3). Statistical significance was determined using the Student's *t*-test or one-way and two-way ANOVA test with a *post hoc* Bonferroni test. Asterisks (*p < 0.05, **p < 0.01, *#p* < 0.001) or different letters signify data that are statistically distinct (*p* < 0.05) in (a, c and f).

Recently, studies have provided support for the proposition that copper- and copper-dependent proteins are promising cancer targets because of their vital roles in cell proliferation, survival, and metastasis, as well as modulators of intracellular redox status.^64–66^ Additionally, significantly elevated CA9 expression levels have been reported in breast cancer CSCs.^41,67,68^ Along with CA9, CD133 is a phylogenetically conserved cell surface marker associated with CSCs.^69^ Therefore, in this study, CD133-positive MDA-MB-231 cells were sorted by magnetic-activated cell sorting (MACS). The cytotoxicity of CA9-BPS-Cu(ii) in CD133-positive MDA-MB-231 cells was significantly increased as compared to CD133-negative cells (Fig. 5a). This higher cytotoxicity might be due to the enhanced expression of CA9 in CD133-positive MDA-MB-231 cells compared to CD133-negative cells (Fig. S27†). It has been proposed that tumour spheroid formation is a characteristic of CSCs.^70^ Primary and secondary sphere-formation assays were conducted with CD133-positive MDA-MB-231 cells using CA9-BPS and CA9-BPS-Cu(ii) to ascertain their effect on the tumour-initiating ability of CSCs as reflected in the formation of tumour spheroids. In fact, the total number of tumour spheroids found in both primary and subsequent secondary sphere-formation assays was reduced upon treatment with CA9-BPS or CA9-BPS-Cu(ii). Importantly, evidence for a synergistic CDT/PDT effect was found in the case the CA9-BPS-Cu(ii) treatment group as compared to the corresponding CA9-BPS treatment group (Fig. 5b and c). Moreover, 4′,6-diamidino-2-phenylindole (DAPI) and propidium iodide (PI) staining for dead cells inside of tumour spheroids provided support for the notion that CA9-BPS-Cu(ii) could penetrate into the target tumour spheroids composed of CD133-positive CSCs, compared to the control and CA9-BPS groups (Fig. 5d).

Next, CD44-positive and CD24-negative cells (a common breast cancer stem cell marker) were obtained from MDA-MB-231 cells using MACS sorting method. Key expression markers were confirmed by immunocytochemistry (Fig. S28†). In order to examine the effect of CA9-BPS-Cu(ii) on tumour evolution and emergence of CSC, an ALDH1 activity assay was performed, since it has been previously shown that CSC expresses high levels of ALDH1 during tumour progression (Fig. S29†).^71^ Treatment with CA9-BPS-Cu(ii) in conjunction with photo-irradiation served to lower the activity of ALDH1 as compared to what was found for the CA9-BPS treated cells.

We thus conclude that the CSC ability was reduced not only in CD133 but also in CD44-positive and CD24-negative cells. Accordingly, we suggest that the synergistic targeting of oxidative stress by CA9-BPS-Cu(ii) might allow for an effective entry into CSC eradication-focused cancer therapy.^64,72^

It was also found that photo-irradiation in the presence of CA9-BPS-Cu(ii) decreased so-called stemness (octamer-binding transcription factor-4; Oct4 and homeobox protein; Nanog), which is one of the criteria for CSCs. The corresponding protein expression was observed by immunocytochemistry of the tumour spheroids (Fig. 5e and f). The ROS generated by CA9-BPS-Cu(ii) under photo-irradiation conditions increased the cytotoxicity of CD133-positive CSCs, presumably due to increasing ROS stress and escaping stemness. Gene and protein expression of Sox9 (SRY-Box Transcription Factor 9), required for the expression of CSCs in breast luminal progenitor^73^ and Stat3 (signal transducer and activator of transcription 3), an important transcriptional factor for normal stem cells and CSCs^74^ was also decreased when CD133-positive CSCs were treated with CA9-BPS-Cu(ii) under conditions of photo-irradiation (Fig. 5e and f). Therefore, the results of our tumour spheroid model, mimicking *in vivo* differentiation effect on CSCs, provide support for our central hypothesis, namely that the combined effect of CDT and PDT embodied in CA9-BPS-Cu(ii) triggers apoptosis in cancer cells and modulates the stemness of CSCs.

### *In vivo* xenograft tumour imaging and photocytotoxic effects

**Fig. 6:**
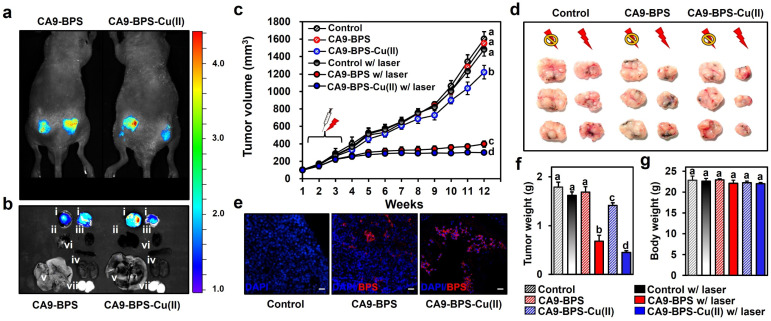
*In vivo* diagnostic and photo-cytotoxic effects of CA9-BPSs. (a) *In vivo* images of nude mice 4 h after tail-vein injection of CA9-BPSs. (b) Fluorescent *ex vivo* images of various organs (i: tumour, ii: lung, iii: heart, iv: spleen, v: liver, vi: kidney, vii: testis) taken from nude mice 6 h after tail vein injection of CA9-BPS-Cu(ii) (right) or CA9-BPS (left). (c) Tumour volumes of the mice in the CA9-BPS-Cu(ii) and CA9-BPS groups with or without PDT treatment. (d) Dissected tumours from each group. (e) Fluorescent images of 6 μm cryo-sectioned tumour tissue stained with DAPI (nuclei, blue) taken from each group after treatment. The red fluorescence is ascribed to the CA9-BPSs under study. Magnification: 100×. Scale bars: 30 μm. (f) Tumour weight and (g) body weight of the mice in the CA9-BPS-Cu(ii) or CA9-BPS groups with or without PDT treatment. Data are presented as the mean, while the error bars indicate the standard deviation from the mean (*n* = 6). Statistical significance was determined using one-way and two-way ANOVA tests with associated *post hoc* Bonferroni tests. Different letters (a–d) in (c and f–g) signify data that are statistically distinct (*p* < 0.05).

To test the *in vivo* anti-tumour efficacy of CA9-BPS-Cu(ii), xenograft mice models having two tumours in both femoral regions were prepared by inoculating MDA-MB-231 cells. CA9 expression within the tumour was established *via* immunohistochemical staining of cryo-sectioned tumour tissue (Fig. S30†). To assess the tumour-targeting efficiency of CA9-BPSs, *in vivo* and *ex vivo* fluorescence of xenograft mice was monitored after tail-vein injections of CA9-BPSs. Bright fluorescence ascribable to CA9-BPS-Cu(ii) was observed at both tumour sites with greater intensities than that observed for other organs, including the heart, liver, spleen, lung, kidney, and testis (Fig. 6a and b). After a total of three injections (tail-vein injections 1 time a week for 3 weeks) of CA9-BPSs and following photo-irradiation (2.0 W cm^−2^, 10 min; 1200 J cm^−2^), a significant reduction in the size of the tumour was observed in the CA9-BPS-Cu(ii) treatment group relative to the control and CA9-BPS treatment groups (Fig. 6c and d). HPF imaging of excised tumours showed that the fluorescence intensity ascribable to HPF in the tumour tissue taken from the CA9-BPS-Cu(ii) treatment group was greater than what was seen for the corresponding tissues for the CA9-BPS treated mice (Fig. S31†). We thus speculate that chemodynamic process could occur upon administration of CA9-BPS-Cu(ii) in tumours and that these effects might be enhanced by PDT-induced ROS production.

Within the tumour tissues, the classic blue fluorescence ascribed to DAPI was minimal in the case of the CA9-BPS-Cu(ii) treatment group as compared to the PBS and CA9-BPS treatment groups (Fig. 6e). Such findings are consistent with apoptosis being increased in the case of the CA9-BPS-Cu(ii) treatment group. Further, quantitative analyses of the tumour weights (Fig. 6f) revealed a superior tumour suppression efficacy of CA9-BPS-Cu(ii), without an obvious effect on the overall body weight (Fig. 6g). A beneficial effect was observed for non-irradiated CA9-BPS-Cu(ii) and irradiated CA9-BPS, although to a lesser extent than with irradiated CA9-BPS-Cu(ii) (Fig. 6c–f). Similar anti-tumour effects were seen for CA9-BPSs when the same photocytotoxic experiments were performed using xenograft mice models having MDA-MB-453 cell lines that express high levels of CA9 (Fig. S32†). In addition to the CA9-high MDA-MB-231 and MDA-MB-453 tumour-bearing xenograft mice studies, the utility of CA9-BPSs was also assessed *in vivo* using CA9-low T47D tumour-bearing xenograft mice. These analyses revealed that neither CA9-BPS-Cu(ii) nor CA9-BPS provided a significant therapeutic effect (Fig. S33†). This finding thus provides support for the design predicate that CA9-BPS and CA9-BPS-Cu(ii) possess a high affinity for CA9 and can be effective in treating tumours that express CA9.

## Conclusions

We successfully prepared a new Cu(ii)-BODIPY PS complex (CA9-BPS-Cu(ii)) containing a CA9-targeting ligand, acetazolamide, and demonstrated its efficacy in promoting a synergistic CDT/PDT effect with CSC targeting to enhance cancer therapy *in vitro* and *in vivo*. Compared to the metal-free system CA9-BPS, CA9-BPS-Cu(ii) exhibited intensified cytotoxicity against MDA-MB-231 cells under 660 nm laser photo-irradiation. This improvement is attributed to the Cu(i)-mediated production of ROS, a CDT effect enhanced by PDT-induced ROS production and, in part, by copper-catalysed glutathione depletion. As inferred from studies with CD133-positive and CD133-negative MDA-MB-231 cells obtained by MACS, the CA9-targeting conferred by the acetazolamide subunit accounts for its CSC targeting ability. Finally, the *in vivo* studies validated its efficacy against tumour growth. Overall, the findings suggest that a higher efficacy of the combination of PDT and CDT, coupled with targeted ROS production, than the individual components. We believe that CA9-BPS-Cu(ii) may have a role to play in controlling tumour regrowth and cancer metastasis and could prove particularly effective in targeting and eradicating CSCs.

## Ethical statement

All animal studies were performed in strict accordance with the Korean Animal Protection Act. guidelines (Act. No. 14651, 2017, https://elaw.klri.re.kr/eng_service/lawView.do?hseq=42743&lang=ENG) for the care and use of laboratory animals and was approved by the Korea University Institutional Animal Care and Use Committee (IACUC, Approved as study No. KUIACUC-2019-0090) of Central Laboratory Animal Research Center (Seoul, Korea).

## Data availability

The experimental detail and datasets supporting this article are available in the ESI.†

## Author contributions

H. S. J., J. L. S., J. H. and J. S. K. conceived the methodology and supervised the project. H. S. J. and S. K. contributed to the project design and, along with S. A. and H. P. carried out the synthetic experimental work. J. H. and M. W. performed the biological experiments and statistical analyses. All authors prepared and edited the manuscript.

## Conflicts of interest

The authors declare no competing interest.

## Supplementary Material

SC-014-D2SC03945A-s001
