# Supplementary material for: Cu(ii)-BODIPY photosensitizer for CAIX overexpressed cancer stem cell therapy
Source: Chem Sci. 2023 Jan 19;14(7):1808–19. doi: 10.1039/d2sc03945a (PMC9930985; doi:10.1039/d2sc03945a)
Supplement: SC-014-D2SC03945A-s001 [file SC-014-D2SC03945A-s001.pdf]

Electronic Supplementary Material (ESI) for Chemical Science.
This journal is © The Royal Society of Chemistry 2023

Supporting Information for:

Cu(II)-BODIPY photosensitizer for CAIX overexpressed cancer stem cell therapy

Hyo Sung Jung,*‡[a] Seyoung Koo,‡[b] Miae Won,‡[b] Seeun An,[a] Haebeen Park,[a] Jonathan L. Sessler,*[c] Jiyou Han,*[a] and Jong Seung Kim*[b]

[a] Department of Biological Sciences, Hyupsung University, Hwasung-si, 18330, Korea.
[b] Department of Chemistry, Korea University, Seoul 02841, Korea.
[c] Department of Chemistry, The University of Texas at Austin, Austin, Texas 78712-1224, United States.
‡These authors contributed equally.

*e-mail: hs0101j@gmail.com; sessler@cm.utexas.edu; hanjiyou12@hanmail.net; jongskim@korea.ac.kr

Table of Contents

1. General information and materials---S3
2. Synthesis of **CA9-BPS-Cu(II)** and **CA9-BPS** --S3
3. Spectroscopic data --S6
4. Fluorescence quantum yield (Φf) calculations---S6
5. Analyses of singlet oxygen generation ---S6
6. Analyses of photothermal performance --S7
7. Copper(II) reduction assay---S7
8. Copper(I) release assay--S7
9. Hydroxyl radical generation assays---S7
10. Western blotting-- - S8
11. Generation of knockout cell lines with CRISPR-Cas9 system---------------------------------- - S9
12. Cytotoxicity analyses-- - S9
13. Combination index (CI) analyses -- S10
14. Co-localization experiments -- S11
15. Intracellular ROS measurements--- S11
16. Intracellular thiol detection -- S12
17. Inductively coupled plasma-mass spectrometry-- S12
18. Real-time PCR --- S13
19. Cancer stem cell-like cell sorting using MACS -- S13
20. Tumor spheroids formation and PI cytotoxicity-- S14
21. Aldefluor assay-- S15
22. CD44-positive/CD24-negative staining --- S15
23. Immunocytochemistry of cryosectioned tumor spheroid and tumor tissues------------------ S16
24. Mouse xenograft model --- S17
25. In vivo diagnostic imaging-- S17
26. In vivo photo-cytotoxic efficacy --- S18
27. Statistical analysis --- S18
28. Supplementary figures -- S19
29. ^{1}H NMR and ^{13}C NMR spectra--- S38
30. ESI MS data --- S45
31. Original western blots images --- S49
32. References-- S51

1. General information and materials

UV-Vis and fluorescence spectra were recorded using a Jasco V-750 spectrophotometer and a Shimadzu RF-5301PC spectrofluorometer, respectively. ESI-MS and MALDI-TOF/TOF-MS spectra were obtained using Shimadzu LCMS-2020 and Bruker Ultraflextreme (Korea Basic Science Institute) instruments, respectively. 1H and 13C NMR spectra were collected on Varian 400 MHz NMR or Bruker 500 MHz NMR spectrometers. Reverse-phase HPLC analyses were performed on a XBridge® C18 (4.6 × 250 mm, 5 μm) column with a Waters Arc HPLC system. All chemicals were purchased from Aldrich, except for acetazolamide (TCI) and O-(1H-Benzotriazol-1-yl)-N,N,N',N'-tetramethyluronium tetrafluoroborate (Alfa Aesar), and were used as received. Column chromatography was performed using silica gel 60 (70-230 mesh) as the stationary phase. Analytical thin layer chromatography was performed using 60 silica gel (pre-coated sheets with 0.25 mm thickness).

2. Synthesis of CA9-BPS-Cu(II) and CA9-BPS

Compounds **10**,[S1] **7**,[S2] and **6**[S3] were synthesized according to reported procedures.

Compound 9: A mixture of **10**[S1] (0.18 g, 0.65 mmol), HATU (0.25 g, 0.65 mmol) and DIPEA (0.11 mL, 0.65 mmol) in DMF (2 mL) was stirred at rt for 20 min. 2-Azidoethylamine (0.11 g, 1.3 mmol) and triethylamine (0.18 mL, 1.3 mmol) were then added at rt and the mixture was stirred for an additional 6 h at rt. After removal of the volatiles under reduced pressure, the crude product was purified over silica gel using CH_2Cl_2/MeOH (v/v, 95:5) as the eluent to yield **9** as a white solid (0.14 g, 62%): ^{1}H NMR (DMSO-*d*6): δ 13.0 (s, 1H), 8.34 (br, 2H), 8.21 (t, *J* = 5.50 Hz, 1H), 3.38-3.28 (m, 4H), 3.24 (t, *J* = 6.95 Hz, 2H), 2.76 (t, *J* = 6.95 Hz, 2H). ^{13}C NMR (DMSO-*d*6): δ 171.8, 171.1, 164.2, 161.2, 50.0, 38.3, 30.2, 29.4. ESI-MS calc. for $C_8H_{12}N_8O_4S_2$ $[M+H]^+$ 349.04, found 349.25.

Compound 5: A mixture of **6**[S3] (0.14 g, 0.76 mmol), propargyl bromide (0.088 mL, 0.99 mmol) and K_2CO_3 (0.5 g, 1.53 mmol) in DMF (4 mL) was heated at 50 °C overnight. After removal of the remaining K_2CO_3 by filtration, the filtrate was evaporated under reduced pressure. The crude product obtained in this way was purified over silica gel using n-hexane/EtOAc (v/v, 4:1) as the eluent to yield compound **5** as a brown oil (0.10 g, 61%): ^{1}H NMR ($CDCl_3$): δ 8.59 (d, *J* = 4.80 Hz, 1H), 7.60 (t, *J* = 8.32 Hz, 1H), 7.33 (d, *J* = 7.99 Hz, 1H), 7.26-7.21 (m, 2H), 7.15 (t, *J* = 7.65 Hz, 1H), 6.85 (d, *J* = 6.84 Hz, 2H), 6.79 (t, *J* = 7.99 Hz, 1H), 4.70 (s, 2H), 4.15 (s, 2H), 2.25 (s, 1H). ^{13}C NMR ($CDCl_3$): δ 159.3, 149.8, 148.3, 137.1, 129.5, 129.4, 122.3, 121.4,

118.6, 114.1, 114.0, 79.9, 72.6, 57.8, 41.2. ESI-MS calc. for $C_{15}H_{14}N_2$ $[M+H]^+$ 223.12, found 223.10.

Compound 4: Compound **5** (0.20 g, 0.90 mmol) was formylated by adding DMF (0.56 mL, 7.2 mmol) and $POCl_3$ (0.35 g, 2.3 mmol) and stirring at rt for 30 min, followed by heating at 90 ^oC for 2 h. After completion of the reaction, the mixture was allowed to cool to rt, ice water was added, and the solution was neutralized to pH 7 by adding 1N aqueous NaOH. The mixture was extracted with EtOAc, washed with water, and dried, followed by purification by column chromatography over silica gel. Yield: 0.18 g, 80%; ^{1}H NMR ($CDCl_3$): δ 9.77 (s, 1H), 8.61 (d, *J* = 8.20 Hz, 1H), 7.75 (d, *J* = 6.90 Hz, 2H), 7.63 (t, *J* = 7.65 Hz, 1H), 7.24-7.19 (m, 2H), 6.88 (d, *J* = 4.50 Hz, 2H), 4.81 (s, 2H), 4.29 (s, 2H), 2.33 (s, 1H). ^{13}C NMR ($CDCl_3$): δ 190.7, 157.5, 152.6, 150.1, 137.2, 132.3, 132.2, 127.2, 122.7, 121.1, 112.7, 112.6, 78.7, 73.3, 57.4, 41.4. ESI-MS calc. for $C_{16}H_{14}N_2O$ $[M+H]^+$ 251.11, found 251.00.

Compound 3: A mixture of 2,4-dimethylpyrrole (0.23 g, 2.4 mmol), **4** (0.28 g, 1.1 mmol) and trifluoroacetic acid (2 drop) in CH_2Cl_2 was stirred for 6 h at rt. *p*-Chloranil (0.27 g, 1.1 mmol) was then added and the mixture was stirred at rt for 1 h. Triethylamine (3 mL) and boron trifluoride diethyl etherate (3 mL) were then added sequentially and after stirring for 30 min. at rt, the crude product was extracted with CH_2Cl_2, washed with water and then brine. The CH_2Cl_2 layer was dried over anhydrous $MgSO_4$. After removal of the volatiles under reduced pressure, the crude product was purified over silica gel using n-hexane/EtOAc (v/v, 4:1) as the eluent to yield **3** as an orange solid (0.23 g, 45%): ^{1}H NMR ($CDCl_3$): δ 8.60 (d, *J* = 4.84 Hz, 1H), 7.63 (t, *J* = 8.01 Hz, 1H), 7.29 (d, *J* = 7.73 Hz, 1H), 7.19 (t, *J* = 7.46 Hz, 1H), 7.06 (d, *J* = 8.39 Hz, 2H), 6.92 (d, *J* = 8.56 Hz, 2H), 5.97 (s, 2H), 4.76 (s, 2H), 4.22 (s, 2H), 2.54 (s, 6H), 2.29 (s, 1H), 1.46 (s, 6H). ^{13}C NMR ($CDCl_3$): δ 158.4, 155.1, 155.0, 149.8, 148.6, 143.3, 142.7, 142.6, 136.8, 132.1, 128.9, 124.4, 124.3, 122.3, 121.4, 121.1, 121.0, 114.3, 79.2, 72.8, 57.4, 41.2, 14.8, 14.7, 14.6, 14.5.. ESI-MS calc. for $C_{28}H_{27}BF_2N_4$ $[M+H]^+$ 469.23, found 469.15.

Compound 2: To a solution of **3** (0.16 g, 0.35 mmol) in ethanol (5 mL) was added solution of *N*-bromosuccinimide (NBS) (0.12 mg, 0.70 mmol) in ethanol (5 mL). The mixture was then stirred for 1 h at rt. After the extraction and washing with water, the organic layer was dried with $MgSO_4$. After filtration and removal of the volatiles, the crude product was purified over silica gel using n-hexane/EtOAc (v/v, 4:1) as the eluent. This gave **2** as a pink-orange solid (0.22 g, 91%): ^{1}H NMR ($CDCl_3$): δ 8.61 (d, *J* = 8.60 Hz, 1H), 7.64 (t, *J* = 8.70 Hz, 1H), 7.29 (d, *J* = 7.73 Hz, 1H), 7.20 (t, *J* = 7.46 Hz, 1H), 7.03 (d, *J* = 8.39 Hz, 2H), 6.93 (d, *J* = 8.56 Hz, 2H), 4.78 (s, 2H), 4.26 (s, 2H), 2.57 (s, 6H), 2.30 (s, 1H), 1.46 (s, 6H). ^{13}C NMR ($CDCl_3$): δ

158.1, 153.4, 153.3, 149.8, 148.9, 143.2, 140.7, 140.5, 136.8, 131.0, 128.8, 123.4, 123.3, 122.4, 114.4, 114.2, 111.5, 111.4, 79.0, 72.9, 57.4, 41.2, 14.0, 13.9, 13.7, 13.6. ESI-MS calc. for $C_{28}H_{25}BBr_2F_2N_4$ $[M+H]^+$ 625.05, found 625.05.

Compound 1: A mixture of **2** (0.44 g, 0.71 mmol), **7**[S2] (0.46 g, 1.71 mmol), glacial acetic acid (1.2 mL), piperidine (1.2 mL) and a small amount of $Mg(ClO_4)_2$ in toluene (60 mL) was heated at reflux overnight. The water formed during the reaction was removed with a Dean−Stark trap. After removal of the volatiles under reduced pressure, the crude product was purified over silica gel using CH_2Cl_2/MeOH (v/v, 95:5) as the eluent to yield **1** as a dark green solid (0.29 g, 44%): ^{1}H NMR ($CDCl_3$): δ 8.62 (d, *J* = 8.61 Hz, 1H), 8.08 (d, *J* = 16.33 Hz, 2H), 7.65 (t, *J* = 8.70 Hz, 1H), 7.63-7.58 (m, 6H), 7.30 (d, *J* = 7.62 Hz, 1H), 7.20 (t, *J* = 7.25 Hz, 1H), 7.07 (d, *J* = 8.70 Hz, 2H), 6.97-6.93 (m, 6H), 4.78 (s, 2H), 4.25 (s, 2H), 4.20-4.17 (m, 4H), 3.90-3.87 (m, 4H), 3.77-3.74 (m, 4H), 3.72-3.69 (m, 4H), 3.68-3.65 (m, 4H), 3.57-3.55 (m, 4H), 3.38 (s, 6H), 2.31 (s, 1H), 1.49 (s, 6H). ^{13}C NMR ($CDCl_3$): δ 159.9, 158.1, 149.8, 148.9, 148.1, 141.1, 139.8, 138.5, 136.8, 132.6, 130.0, 129.3, 129.2, 124.0, 122.3, 121.3, 116.2, 115.0, 114.3, 109.9, 79.0, 72.8, 72.0, 70.9, 70.7, 70.6, 69.7, 67.6, 59.1, 57.4, 41.2, 14.0. ESI-MS calc. for $C_{56}H_{61}BBr_2F_2N_4O_8$ $[M+H]^+$ 1125.29, found 1125.05.

CA9-BPS: A mixture of **1** (0.16 g, 0.14 mmol), **9** (0.049 mg, 0.14 mmol) and sodium ascorbate (10 mol %) in DMF:MeOH (2:1 mL) was stirred for 15 min at rt. Then 5 mol % of $CuSO_4$ in 0.5 mL water was added to the reaction mixture, which was stirred for 6 h. After removal of the solvents under reduced pressure, the crude mixture was purified over silica gel using CH_2Cl_2/MeOH (v/v, 95:5) as the eluent to yield **CA9-BPS** as a dark green solid (0.12 g, 58%): ^{1}H NMR (DMSO-d_6): δ 13.04 (br, 1H), 8.57 (d, *J* = 4.18 Hz, 1H), 8.31 (br, 2H), 8.16 (t, *J* = 7.17 Hz, 1H), 8.08 (s, 1H), 8.02 (d, *J* = 16.60 Hz, 2H), 7.74 (t, *J* = 8.64 Hz, 1H), 7.59 (d, *J* = 8.24 Hz, 4H), 7.45 (d, *J* = 16.60 Hz, 2H), 7.27 (t, *J* = 7.76 Hz, 1H), 7.21 (d, *J* = 7.88 Hz, 1H), 7.13 (d, *J* = 8.51 Hz, 2H), 7.06 (d, *J* = 8.24 Hz, 4H), 7.01 (d, *J* = 8.51 Hz, 2H), 4.81 (d, *J* = 11.36 Hz, 4H), 4.41 (t, *J* = 7.75 Hz, 2H), 4.20-4.14 (m, 4H), 3.80-3.74 (m, 4H), 3.62-3.57 (m, 4H), 3.57-3.53 (m, 4H), 3.53-3.50 (m, 4H), 3.48 (t, *J* = 7.75 Hz, 2H), 3.45-3.41 (m, 4H), 3.24 (s, 6H), 2.74 (t, *J* = 7.54 Hz, 2H), 2.47 (t, *J* = 7.54 Hz, 2H), 1.9 (residual ethyl acetate), 1.46 (s, 6H), 0.86 and 1.25 (residual n-hexane). ^{13}C NMR (DMSO-d_6): δ 172.3, 171.7, 167.5, 165.5, 164.6, 161.8, 160.4, 158.7, 149.8, 149.7, 147.5, 144.2, 141.7, 141.5, 138.7, 137.2, 134.2, 132.6, 132.2, 132.1, 130.0, 129.6, 129.4, 129.3, 129.1, 124.1, 122.7, 121.6, 115.8, 115.6, 113.7, 109.8, 71.8, 70.4, 70.3, 70.1, 69.3, 68.3, 67.9, 67.6, 58.5, 56.1, 49.2, 46.6, 38.7, 36.8, 33.5, 32.0, 31.7, 31.1, 30.7, 30.4, 29.8, 29.5, 28.8, 26.2, 23.9, 22.9, 22.5, 19.8, 14.6, 14.4, 14.2, 11.4. ESI-MS

calc. for $C_{64}H_{73}BBr_2F_2N_{12}O_{12}S_2$ $[M+H]^+$ 1475.33, found 1475.20.

CA9-BPS-Cu(II): To stirred solution of **CA9-BPS** (0.15 g, 0.10 mmol) in 5 mL of acetonitrile, $Cu(ClO_4)_2{\cdot}6H_2O$ (1 equiv.) was slowly added, and the reaction mixture was stirred at rt for 1 h. After removal of the solvents under reduced pressure, the residue was purified using HPLC (C18, 5 μm, 4.6 × 250 mm as the stationary phase; buffer A (deionized water containing 0.1% TFA) or buffer B (acetonitrile) were used as the mobile phases. **CA9-BPS-Cu(II)** was eluted at 50-70% of buffer B for 5 min, then 70-85% for 3 min, then 85-100% for 4 min, then 100% for 8 min, then 100-50% for 10 min. The solution was evaporated, which gave a dark green powder (0.24 mg, 80%). The HPLC chromatogram of **CA9-BPS-Cu(II)** is shown in Fig. S5. HPLC retention time: 7.85 min, ESI-MS calc. for $C_{64}H_{73}BBr_2CuF_2N_{12}O_{12}S_2$ $[M+H]^+$ 1536.26, found 1536.75.

3. Spectroscopic data

For UV/Vis and fluorescence spectra measurements, a dimethyl sulfoxide (DMSO) stock solution of CA9-BPS-Cu(II) and CA9-BPS was diluted with aqueous buffer (10 mM PBS, pH 7.4, 5% DMSO) or ethanol, to give a 5 μM solution. For fluorescence spectral measurements, samples were excited at 660 nm using excitation and emission slit widths of 20 nm.

4. Fluorescence quantum yield (Φ_f) calculations

Fluorescence quantum yields were determined by comparing the fluorescence intensity of the sample (*PS*: photosensitizer) with that of a fluorescence standard (*R*: reference) using the following equation:

$$\Phi_f(PS) = \Phi_f(R)\left(\frac{A_{PS}}{A_R}\right)\left(\frac{OD_R}{OD_{PS}}\right)\left(\frac{n_{PS}^2}{n_R^2}\right) \quad \text{----- Eqn. S1}$$

Φ_f is the quantum yield, A is the integrated area under the corrected fluorescence spectra, OD is the optical density, and n is the refractive index. The subscript PS and R refer to the sample and reference, respectively. Zinc phthalocyanine, for which the fluorescence quantum yield (Φ_f) in toluene containing 1.0 % pyridine is 0.30, was used as the reference.[S4]

5. Analyses of singlet oxygen generation

The generation of singlet oxygen was measured by mixing 80 μM 1,3-diphenylisobenzofuran (DPBF) and 1 μM PS in acetonitrile with adjusting the absorbance of DPBF and PS to around 1.0 and 0.1, respectively. In the case of studying the effect of thiol, Na_2S (1eq) was pre-

incubated with **CA9-BPS-Cu(II)**. After measuring the UV-Vis spectrum in the dark, the samples were exposed to monochromatic light at 660 nm (Xenon Lamp Monochromatic Light Source, slit width 15/1.5 nm) for 3 minutes. After each irradiation, UV-Vis spectra were collected. The absorbance of DPBF at 412 nm was plotted versus time and the slope was determined.

6. Analyses of photothermal performance

The photothermal performance of **CA9-BPS-Cu(II)** (0.5 mM) and **CA9-BPS** (0.5 mM) in cell lysate (MDA-MB-231; 20 μg/mL)-PBS solutions (10 mM, pH 7.4) was assessed by subjecting to continuous photo-irradiation (660 nm laser; 2 W/cm^2; 10 min; 1200 J/cm^2). IR thermal images were recorded every 1 min using an infrared thermal imaging camera (FLIR, E60, USA). The temperature change (ΔT) was then plotted versus time to generate the photothermal heating curves presented in the main text.

7. Copper(II) reduction assay

Copper(II) reduction was monitored using bathocuproinedisulfonic acid disodium salt (BCDS) according to a reported procedure.[S5] For these analyses, **CA9-BPS-Cu(II)** (30 μM) was mixed with 10 equiv. of glutathione (GSH), cysteine (Cys), ascorbic acid (AA) and 2 equiv. of BCDS in 1) MeOH/10 mM PBS (v/v, 1/1) at pH 7.4, 2) 0.1% DMSO, RPMI media (10% FBS, without phenol red). Before collecting the absorption spectrum, each sample was slowly filtered using a syringe filter (Whatman™, 0.45 μm PTFE).

8. Copper(I) release assay

Intracellular Copper(I) release was monitored using BioTracker GREEN Copper Dye (GORYO Chemical #GC902, Sapporo, Japan) according to a reported procedure.[S6] For fluorescence imaging, MDA-MB-231 cells were seeded and allowed to grow to 2.0 × 10^5 per dish in 35-mm glass bottom confocal dishes (SPL). Cells were incubated with medium containing **CA9-BPS** (20 μM), **CA9-BPS-Cu(II)** (20 μM) and $CuCl_2$ (20 μM) for 12 h. The cells were then washed three times with PBS. 5 μM BioTracker GREEN Copper Dye (Ex/Em 480/510 nm) was added and the cells were incubated for 30 min. The cells were imaged with a FLUOVIEW FV3000 confocal laser scanning microscope (Olympus, Shinjuku, Tokyo, Japan).

9. Hydroxyl radical generation assays

The generation of hydroxyl radical was measured using terephthalic acid (TPA) according to a reported procedure.[S7] For these analyses, a 2.5 mM TPA solution was made up in a 2 mM aqueous NaOH medium. Then, **CA9-BPS** (20 μM) or **CA9-BPS-Cu(II)** (20 μM) was mixed with this TPA solution (20 μM), along with GSH (5 mM) and H_2O_2 (20 mM) in 0.1 M PBS (0.15 % DMSO, pH 7.4). After 1 h, each sample was excited at 312 nm and the fluorescence intensity at 440 nm was recorded.

10. Western blotting

To measure endogenous CA9, CA2 and CA12 expression, normal cell lines (human mammary epithelial MCF10A) as well as breast cancer cell lines [TNBC (MDA-MB-231, Hs578T), including ER-positive (MCF7, T47D, ZR-75-1) and HER2-positive (SK-BR-3, BT-474, MDA-MB-453) cells] were analyzed. Briefly, cells (2 × 10^6) were seeded in 100 mm culture dishes and cultured for 48 h. To investigate apoptosis-related signaling pathways, MDA-MB-231 (2 × 10^6) cells were seeded in 100 mm culture dishes under normoxic (O_2, 21%) and hypoxic (O_2, 3%) conditions. After incubation for 24 h, the cells were treated with 1% DMSO (as a control), 10 μM **CA9-BPS-Cu(II)**, or 10 μM **CA9-BPS**. After allowing to incubate for an additional 4 h, the cells were irradiated with a 660 nm lamp (100 mW/cm^2, 1 min; 6 J/cm^2) for 1 h, before western blotting was performed. The cells were then solubilized in RIPA lysis buffer [30 mM NaCl, 0.5% Triton X-100, 50 mM Tris-HCl (pH 7.4)] containing protease inhibitor cocktail. The supernatant was collected after centrifugation (13,000 rpm, 4 °C, 60 min) and protein concentrations were measured with a Bradford protein assay kit (Bio-Rad Laboratories). Equal quantities of protein (30 μg) were subjected to SDS-PAGE and electro transferred onto a nitrocellulose membrane (Merk Millipore, Burlington, MA, USA). The membranes were incubated overnight at 4 °C with primary antibodies (CA9: Santa Cruz Biotechnology sc-365900, CA2: Santa Cruz Biotechnology sc-48351, CA12: Santa Cruz Biotechnology sc-374313, b-actin: Santa Cruz Biotechnology sc-47778, CD133: Abcam #19898, Bax: Santa Cruz Biotechnology sc-47778, Bcl-2: Santa Cruz Biotechnology sc-65392, Cleaved caspase 3: Cell Signaling Technology #9661), diluted in 5% BSA. The resulting membranes were washed with Tris-buffered saline 0.1% tween-20 (TBS-T) and then incubated with anti-mouse horseradish peroxidase (HRP)-conjugated secondary antibodies (Santa Cruz Biotechnology; sc-2357) and anti-rabbit horseradish peroxidase (HRP)-conjugated secondary antibodies (GTX213110-01; GeneTex, Irvine, CA, USA) for 2 h at room temperature. To detect

immunoreactive protein bands, an enhanced chemiluminescence reagent (Luminate; Merk Millipore) was used according to the manufacturer's instructions.

11. Generation of knockout cell lines with CRISPR-Cas9 system

The CRISPR-Cas9 system was prepared as follows: The CA9-guide-RNA targeting of the CA9 gene were designed #1 (Exon1, human CA9) and #2 (Exon3, human, CA9) from Bioneer company, respectively. The complementary oligonucleotides for guide RNAs (gRNAs): #1) 5′-CCACCCGGAGAGGAGGATCTACC-3′ and 5′-GGTGGGCCTCTCCTCCTAGATGG-3′, #2 5′-CCAGTCCCCGGTGGATATCCGCC-3′ and 5′-GGTCAGGGGCCACCTATAGGCGG -3′). To assess the completely response, MDA-MB-231 cells were transfected with CA9 gRNA #1 and #2 using Lipofectamine CRISPRMAX Cas9 Transfection Reagent (Invitrogen) according to manufacturer's instructions. Briefly, prior to treatment, Lipofectamine CRISPRMAX Cas9 Transfection Reagent/Cas9 nuclease-gRNA complex mixtures were prepared in reduced serum medium, Opti-MEM (Invitrogen), at the recommended ratio of 10 μL Lipofectamine CRISPRMAX Cas9 Transfection Reagent per 10 μM gRNA. Cells were then treated for 4 hours before being replaced with complete medium for the desired duration. One days after transfection, cells were diluted 96 well plate for single colony formation. After 10 days, colonies were isolated and analyzed by western blotting for transfection efficiency.

12. Cytotoxicity analyses

To determine the cytotoxicity, a Cellomax Cell Viability Kit (Precaregene, Hanam, Gyeonggi-do, Korea) was used according to the manufacturer's instructions in the presence of the three test agents under study. Briefly, CA9-endogenous and CA9 knockout cells (MDA-MB-231), CA9-negative cells (MCF7, T47D and BJ) cells, and CA9-negative/CA12-positive cells (BT-474) were seeded 1.0×10^4 per each well in a 96-well plate. Twenty-four hours after seeding, the culture medium was exchanged with fresh medium (80 μL) containing 0, 2.5, 5, 10, 30, 50 and 80 μM of **CA9-BPS-Cu(II)** or **CA9-BPS**. In the case of experiments involving pre-treatment with *N*-ethylmaleimide (NEM) or acetazolamide, CA9-positive cells (MDA-MB-231) were pre-treated with NEM (1 mM) for 30 min or acetazolamide (0, 5, 10 and 30 μM) for 12 h before the culture medium was exchanged with fresh medium (80 μL) containing **CA9-BPS-Cu(II)**. Cells were incubated at 37 °C for an additional 24 h and then washed with PBS twice. The cells were then irradiated using a 660 nm LED lamp (100 mW/cm^2, 5 min; 30 J/cm^2; DavinchK, Seoul, Korea). Cells were incubated at 37 °C for an additional 12 h and then washed

with PBS twice. The cells were then irradiated using a 660 nm LED lamp (100 mW/cm^2, 5 min; 30 J/cm^2; DavinchK, Seoul, Korea).

To establish 3% hypoxic and 21% normoxic conditions for examining cytotoxicity, CA9-endogenous and CA9 knockout cells MDA-MB-231 cells (3 × 10^3) or MCF-7 (5 × 10^3) cells were seeded onto 96-well plates (SPL Life Sciences, Gyeonggi-do, South Korea) at a low density. After 12 h, the cells were treated with 250 μM DFO (Deferoxamine, Sigma) in a standard 5% CO_2 incubator for 12 h and then plate was transferred to a hypoxia live cell chamber (Live cell Instrument, Gyeonggi-do, South Korea) containing the appropriate humidified gas mixture (3% O_2, 5% CO_2, and balance of 92% N_2) at 37°C. The O_2 content of the hypoxia live cell chamber was confirmed using a Fyrite gas analyzer (Bacharach, New Kensington, PA, USA). All cell-seeded plates were prepared in triplicate to allow comparisons between normoxic and hypoxic effects (21% vs. 3% O_2). All culture media and phosphate-buffered saline (PBS) were pre-incubated overnight in a standard 5% CO_2 incubator so as to be pre-gassed after purging with pure nitrogen to remove dissolved O_2 before being subject to further incubation in a 3% oxygen environment until the dissolved O_2 levels equilibrated to 3%. Cells were incubated at 37 °C for an additional 6 h and then washed with PBS twice. The cells were then irradiated using a 660 nm LED lamp (100 mW/cm^2, 5 min; 30 J/cm^2; DavinchK, Seoul, Korea). The absorbance of the wells was detected at 450 nm by a Hidex Sense microplate reader (Hidex, Cranbourne, Victoria, AU). Cell viability assays were performed in triplicate and the cytotoxicity was recorded as a percentage calculated for the treated cells relative to the control group.

13. Combination index (CI) analyses

To assess the synergy of the chemodynamic therapy (CDT) and photodynamic therapy (PDT), the so-called combination index (CI) used to quantify synergism or antagonism for two drugs was employed as follows:[S8]

$$Cl = \frac{(D)_1}{(Dx)_1} + \frac{(D)_2}{(Dx)_2} \text{ ----- } \textbf{Eqn. S2}$$

where CI < 1, = 1, and > 1 indicate synergism, additive effect, and antagonism, respectively. In the denominators, $(D_x)_1$ represents D_1 "alone" that inhibits a system x%, and $(D_x)_2$ is for D_2 "alone" that inhibits a system x%. In the numerators, $(D)_1$ and $(D)_2$ "in combination" also inhibit x%. CI was calculated for every dose of two drug pairs. Fraction affected (F_a) is the fractional inhibition of a phenotype by a compound treatment(s). F_a of a group was calculated

as F_a = percent inhibition of cell viability/100. Fraction affected-combination index plot was drawn with every group treated with more than one compound.

14. Co-localization experiments

To access the CA9 uptake ability, CA9-endogenous and CA9 knockout MDA-MB-231 cells (1 × 10^5) were seeded onto 35-mm glass bottom confocal dishes (SPL) under 3% hypoxic and 21% normoxic conditions. Cells were incubated with medium containing **CA9-BPS** (5 μM) and **CA9-BPS-Cu(II)** (5 μM) for 1 h. The cells were then washed three times with PBS. 10 μM Dil (Ex/Em 550/570 nm, cell membrane tracer) was added and the cells were incubated for 30 min. The cells were imaged with a FLUOVIEW FV3000 confocal laser scanning microscope (Olympus, Shinjuku, Tokyo, Japan).

The cells were then washed three times with PBS. To examine the intracellular localization of **CA9-BPS-Cu(II)**, MDA-MB-231 cells were seeded and allowed to grow to 1.0 × 10^5 per dish in 35-mm glass bottom confocal dishes (SPL). Cells were incubated with medium containing **CA9-BPS-Cu(II)** (5 μM) for 24 h. The cells were then washed three times with PBS. Mito-tracker Green FM® (Thermo Fisher Scientific, Inc., New Hampshire, USA, Ex/Em 490/516 nm), ER-tracker Green® (Thermo Fisher Scientific, Inc., Ex/Em 504/511 nm), or Lyso-tracker Green DND-26® (Thermo Fisher Scientific, Inc., Ex/Em 504/511 nm), 50 μL of each, were then added and the cells were incubated for 30 min. The intracellular localization of **CA9-BPS-Cu(II)** was inferred by imaging the overlapping fluorescence signature produced by the test agent and the tracking dye in question using a FLUOVIEW FV3000 confocal laser scanning microscope (Olympus, Shinjuku, Tokyo, Japan). Excitation and emission wavelengths of 639 nm and 650-750 nm, respectively, were used for these latter studies. In order to quantify colocalization of **CA9-BPS-Cu(II)**, the Pearson Correlation Coefficient (PCC) analysis was performed using CellSens (Olympus) program automatically analyzed from the original images.

15. Intracellular ROS measurements

Cells were seeded on a confocal dish (2.0 × 10^5 per dish) and cultured for a day. The cells were then washed with PBS twice before fresh culture medium containing **CA9-BPS-Cu(II)** or **CA9-BPS** (5 μM) was added. After incubating for 24 h, the cells were washed with PBS twice and incubated with either 10 μM DCFH-DA (Invitrogen, Grand Island, NY, USA), 10 μM HPF (Invitrogen), or 10 μM SOSG (Invitrogen) for 30 min. The cells were seeded on a confocal dish (2.0 × 10^6 per dish) and cultured for a day. The cells were then washed with PBS twice

before fresh culture medium containing **CA9-BPS-Cu(II)** or **CA9-BPS** (5 μM) was added. The cells were incubated with either 10 μM DCFH-DA (Sigma) or 10 μM HPF (Thermo Fisher Scientific, Inc.) for 1 h. The cells were washed with PBS and irradiated with a 660 nm laser (100 mW/cm^2, 5 min; 30 J/cm^2). Immediately after termination of the irradiation, images were recorded using a confocal microscope equipped with 485 excitation and 530 emission filters. In order to examine any potential depletion of intracellular GSH levels induced by **CA9-BPS-Cu(II)**, the MDA-MB-231 cells (CA9-positive) and BJ cells (CA9-negative) were pretreated with 100 μM L-buthionine sulfoximine (Sigma) for 24 h. After the incubation, the cells were washed with PBS three times and **CA9-BPS-Cu(II)** or **CA9-BPS** (5 μM, respectively) was added into the culture medium along with 10 μM HPF (Thermo Fisher Scientific, Inc.). After a 1 h incubation period, cells were washed with PBS and irradiated with a 660 nm laser (100 mW/cm^2, 5 min; 30 J/cm^2). Images were recorded immediately using a FLUOVIEW FV3000 confocal laser scanning microscope (Olympus) equipped with 485 excitation and 530 emission filters.

16. Intracellular thiol detection

Cells were seeded on a confocal dish (2.0 × 10^6 per dish), and incubated for a day. The cells were then washed with PBS two times. Additional culture medium containing either **CA9-BPS-Cu(II)** or **CA9-BPS** (5 μM) was then added. After a 24 h incubation period, the cells were irradiated with a 660 nm laser for 5 min. Six hour later, the cells were washed twice with thiol-free DPBS without Ca^{2+} or Mg^{2+}. ThiolTracker Violet® (20 μM) in thiol-free DPBS without Ca^{2+} or Mg^{2+} was then added into the culture dish, which was allowed to sit for 30 min. Cell images were taken using a FLUOVIEW FV3000 confocal laser scanning microscope (Olympus) equipped with 405 excitation and 525 emission filters. A blue pseudo color was used for the ThiolTracker®.

17. Inductively coupled plasma-mass spectrometry

MDA-MB-231 cells (3.0 × 10^6) were seeded in a 100 mm culture dish. After the cells had grown to >80% confluency, each of 100 mm culture dishes was assigned to the control, $CuCl_2$, **CA9-BPS-Cu(II)**, or **CA9-BPS** groups. The cells were washed with PBS and fresh culture medium containing 20 μM of each compound under consideration was added to the culture dishes, which were allowed to incubate for 24 h. The cells were then washed with PBS three times and the cell lysates were collected. Conc. HNO_3 solution was added to the cell pellets

obtained in this way. Then, each sample was subjected to copper concentration analysis using an inductively coupled plasma mass spectrometer system (ICP-MS, Elan DRC II), with the measurements in question being made at the Korea Basic Science Institute.

18. Real-time PCR

To examine the potential mode of action of **CA9-BPS-Cu(II)** and **CA9-BPS**, tumor spheroids of CD133-positive MDA-MB-231 cells were collected after 24 h incubation and irradiation with a 660 nm lamp (100 mW/cm^2, 5 min; 30 J/cm^2). The total RNA from these cells and tumor spheroids was isolated using 200 μL of the TRIzol Reagent (Invitrogen). Traditional reverse transcription studies (Promega, Madison, WI) and real-time PCR (Agilent Technologies, Santa Clara, CA) were carried out according to the manufacturer's instructions. Takara Ex Tag DNA polymerase (Takara, Tokyo, Japan) and SYBR® Green Master Mix (Bio-Rad, Hercules, CA) were utilized for the PCR amplification of different genes using a program of 94 °C for 5 minutes, 35 cycles of 94 °C for 30 seconds, 57 °C-64 °C for 30 seconds, 72 °C for 30 seconds, and extension at 72 °C for 10 minutes. The results were normalized to a GAPDH control. The primers and PCR conditions used are shown below.

Name of Gene	Forward primer	Reverse primer	Annealing Tm (°C)(bp)
**GAPDH**	TTCAGTGGTGGACCTGACCT	CACCACCCTGTTGCTGTAGC	60(256)
**Oct3/4**	TGGGCTCGAGAAGGATGTG	GCATAGTCGCTGCTTGATCG	57(78)
**Nanog**	TGAGCTGGTTGCCTCATGTTAT	GAAGGAAAAGTATCAAGAAATT GGGATA	57(68)
**Sox9**	AGTACCCGCACTTGCACAAC	ACTTGTAATCCGGGTGGTCCTT	64(144)
**Stat3**	ACCTGCAGCAATACCATTGAC	AAGGTGAGGGACTCAAACTGC	62(122)

19. Cancer stem cell-like cell sorting using MACS

MDA-MB-231 cells were cultured with RPMI medium. Sub-populations enriched in CD133-positive and CD44-positive or CD24 negative cells were obtained by means of the MACS immunomagnetic separation system. Briefly, we first prepared PBS, 0.5% bovine serum albumin (BSA) and 2 mM ethylenediaminetetraacetic acid (EDTA) by diluting MACS stock solution with washing buffer. Cells (1×10^8) were suspended in this MACS buffer solution.

They were then incubated with a mouse anti-human CD133 antibody, a mouse anti-human CD44 antibody or a mouse anti-human CD24 antibody conjugated with MicroBeads (Miltenyi Biotech Cat#130-100-857, #130-095-194 and #130-095-951) in the dark at 4 °C for 15 min. The cell suspension was then added to a separation column and eluted. The column was rinsed twice with buffer and separated from the magnetic field, inserted into a fresh tube, before 1~2 mL PBS was administered along the needle core to remove the CD133-positive and CD44-positive or CD24-negative cells. The cells obtained in this way were cultured in neurobasal medium (Invitrogen; Thermo Fisher Scientific, Inc.) containing 1X B27 (Invitrogen; Thermo Fisher Scientific, Inc.), 2 mM L-glutamine, 30 units/mL penicillin-streptomycin (Sigma-Aldrich, St. Louis, MO, USA), 20 ng/mL basic fibroblast growth factor (bFGF; Miltenyi Biotec), and 20 ng/mL epidermal growth factor (EGF; Provitro Biosciences, Mt. Vernon, WA, USA). Both CD133-negative and CD133-positive cells and CD44-positive or CD24-negative cells were collected separately and cultured for further analysis.

20. Tumor spheroids formation and PI cytotoxicity

In order to quantify tumor spheroid formation, briefly, MDA-MB-231 cells (seeding density at the first day of tumor spheroid formation was 1×10^4 cells/ml) were plated in ultralow-attach 24 well plates (CORNING, Corning, NY, USA) and cultured in tumor spheroid media containing RPMI1640 (Gibco), supplemented with B27 (1:50, Invitrogen, Carlsbad, CA, USA), 10 ng/mL basic fibroblast growth factor (bFGF, Invitrogen), 20 ng/mL human epidermal growth factor (EGF, Invitrogen), 1% antibiotic agent, and 15 μg/mL gentamycin for second day. The tumor spheroids were then washed twice with PBS. The tumor spheroid treated with either 10 μM **CA9-BPS-Cu(II)** 10 μM **CA9-BPS** or control (1% DMSO) fresh CSC culture medium, allowed to incubate for 1 days. Then one dish was irradiated with a 660 nm lamp (100 mW/cm^2, 5 min; 30 J/cm^2), and the other dish was not irradiated. A week later, the number of tumor spheroids was counted. The number of tumor spheroids were counted based on spheroid sizes larger than 150 μm in diameter under an Olympus X53 inverted microscope. For second tumor spheroid formation, primary tumor spheroid dissociated with Trypsin/EDTA. And cells (1×10^4 cells/ml) were replated each group in ultralow-attach 24 well plates and cultured in tumor spheroid media for 5 days. The number of tumor spheroids were counted based on spheroid sizes larger than 150 μm in diameter under an Olympus X53 inverted microscope.

To examine cytotoxicity, the counted tumor spheroids (1 x 10^4 cell seeded dishes) of each treatment group were incubated with 1.67 μg/ml propidium iodide (PI, Invitrogen; Thermo

Fisher Scientific, Inc.) for 120 min. After PI incubation, the tumor spheroids were collected in a microtube and fixed in cold 4% paraformaldehyde. The tumor spheroids were then incubated in 20% sucrose overnight at 4 °C for cryo-protection. The fixed the tumor spheroids were embedded in optimum cutting temperature (OCT) compound (Tissue-Tek, Sakura Finetek, Tokyo, Japan) and cut into 6 μm thick sections using a cryostat (Leica CM1950, Leica Biosystems, Wetzlar, Germany). The cryosectioned spheroids were air-dried and counter-stained with 1 μg/ml 4',5-diamidino-2-phenylindole (DAPI; Roche, Indianapolis, IN, USA) and mounted for confocal microscopy (LSM 510 Meta, Carl Zeiss, Oberkochen, Germany).

21. Aldefluor assay

CD44-positive or CD24-negative MDA-MB-231 cells were cultured in neurobasal medium (Invitrogen; Thermo Fisher Scientific, Inc.) containing 1 × B27 (Invitrogen; Thermo Fisher Scientific, Inc.), 2 mM L-glutamine, 30 units/mL penicillin-streptomycin (Sigma-Aldrich, St. Louis, MO, USA), 20 ng/mL basic fibroblast growth factor (bFGF; Miltenyi Biotec), and 20 ng/mL epidermal growth factor (EGF; Provitro Biosciences, Mt. Vernon, WA, USA). When a cell confluency of >80% was reached, the cells (8×10^5) were seeded into 60 mm dishes (Corning, Inc.). After incubation for 24 h, the cells were treated with 1% DMSO (as a control), 10 μM **CA9-BPS-Cu(II)**, or 10 μM **CA9-BPS**. After allowing to incubate for an additional 4 h, the cells were irradiated with a 660 nm lamp (100 mW/cm^2, 5 min; 30 J/cm^2), before an Aldefluor assay kit (Stemcell Technology, Vancouver, BC) was used to assess ALDH1 activity according to the manufacturer's protocol. Briefly, cells were incubated for 45 min at 37° C in the Aldefluor assay buffer containing the ALDH protein substrate BODIPY-amin oacetaldehyde (BAAA, 1 μM per 0.5×10^6 cells). As a specific inhibitor of ALDH1, 7. 5 μM diethylamino-benzaldehyde (DEAB) was used to define the Aldefluor-positive population. Aldefluor-positive stained cells (>10,000 cells) were analyzed using a flow cytometer equipped with a BD LSRFortessa X-20™ cell analyzer (Becton Dickinson, BD, Oxford, UK).

22. CD44-positive/CD24-negative staining

CD44-postive or CD24-negative staining was used to identify Brest cancer stem cell-like cells. Cells were incubated for 12 h at 4 °C with FITC-conjugated anti-CD24 and PE-conjugated

anti-CD44 antibodies (BD Biosciences;BD55542 and BD 561858). Fluorescent images were obtained using a Zeiss LSM510 laser scanning confocal microscope (Carl Zeiss).

23. Immunocytochemistry of cryosectioned tumor spheroid and tumor tissues

Tumor spheroids formed from CD133-positive MDA-MB-231 cells were fixed in cold 4% paraformaldehyde. The tumor spheroids were then incubated in 20% sucrose overnight at 4 ℃ to achieve cryo-protection. The fixed the tumor spheroids were embedded in optimum cutting temperature (OCT) compound (Tissue-Tek, Sakura Finetek, Tokyo, Japan). On the other hand, dissected tumor tissues obtained from MDA-MB-231 cells inoculated xenograft mice (7 weeks after inoculating with cells) were snap-shot frozen and embedded in OCT compound (Tissue-Tek). Both the tumor spheroids and the tumor tissues were cut into 6 μm thick sections using a cryostat (Leica CM1950, Leica Biosystems, Wetzlar, Germany). To allow for immunocytochemical studies, the cryo-sectioned tumor spheroids and tumor tissue were incubated with 10% donkey serum in PBS containing 0.1% BSA with 0.3% Triton X-100. The cryo-sectioned tumor spheroids and tumor tissues were probed with primary antibodies overnight at 4 °C per Table S2. The cryo-sectioned tumor spheroids and tumor tissues were then washed three times with PBS and incubated with secondary antibodies (Invitrogen) conjugated to either Alexa Flour 488 or 594 for 1.5 h at room temperature. Finally, the cryo-sectioned tumor spheroids and tumor tissues were washed with PBS (0.1% BSA) and 1 μg/ml 4’,5-diamidino-2-phenylindole (DAPI; Roche, Indianapolis, IN, USA) were added into the spheroids and tissues to achieve nuclear counterstaining. Fluorescent images were obtained using a Zeiss LSM510 laser scanning confocal microscope (Carl Zeiss).

Table S1. Primary antibody list and dilution factors used for immunocytochemistry.

Name of Protein	Source	Company & Category No.	Dilution factor
**Oct3/4**	Goat anti-human	R&D (AF1759)	1:100
**Nanog**	Mouse anti-human	Santa Cruz (sc-293121)	1:200
**Sox9**	Goat anti-human	R&D (AF3075)	1:50
**Stat3**	Mouse anti-human	Santa Cruz (sc-8019)	1:200
**CA9**	Mouse anti-human	Santa Cruz (sc-365900)	1:200

24. Mouse xenograft model

To examine the efficacy of **CA9-BPS-Cu(II)** or **CA9-BPS** *in vivo*, male BALB/c nude mice (6- to 8-weeks old) were obtained from Central Labs (Seoul, Republic of Korea). The mice were randomly assigned to 6 groups: control w/o or w/ laser, **CA9-BPS-Cu(II)** w/o or w/ laser, and **CA9-BPS** w/o or w/ laser groups (n = 3 per group). Upon arrival, the animals were allowed to acclimatize for two days within the animal facility and maintained according to the Korean University Guidelines for the Care and Use of Laboratory Animals. These guidelines are based on the protocols recommended by the National Institutes of Health, USA. All procedures involving animals were approved by the Korea University Institutional Animal Care and Use Committee (IACUC, Approved as study No. KUIACUC-2019-0090). Approximately 6.0 x 10^6 either MDA-MB-231, MDA-MB-453 or T47D cells were mixed 1:1 with matrigel (BD, San Jose, California, USA) and injected subcutaneously into the right and left femoral region (two sites per mouse; tumor n=6 per treatment group).

25. In vivo diagnostic imaging

Xenografted mice were treated with either **CA9-BPS** or **CA9-BPS-Cu(II)** via tail vein injection (500 µM). After 4 h, the *in vivo* images referred to in the main text were taken using a Maestro™ *In-Vivo* Fluorescence Imaging System (Maestro, CRi Inc., Woburn, MA, USA). Excitation was affected at 639 nm and the emission was monitored over the 650 to 750 nm spectral region. HPF (100 µl of 5 mM per tumor, Invitrogen) was directly injected into tumors, 30 min before photoirradiation with a 660 nm laser (2.0 W/cm^2, 10 min; 1200 J/cm^2). The mice were quickly terminated with CO_2 gas and the tumors were dissected to allow *ex vivo* images of ROS (hydroxyl radicals) generation to be taken using a Maestro™ *In-Vivo* Fluorescence Imaging System (Maestro, CRi Inc., Woburn, MA, USA). Excitation was affected at 500 nm and the emission monitored over the 500 to 700 nm spectral region. For investigating organ distribution of **CA9-BPS-Cu(II),** *ex vivo* images were taken using Maestro™ *In-Vivo* Fluorescence Imaging System (excitation: 639 nm, emission: 650-750 nm). For further analysis, the tumor tissues were snap-frozen after embedded in OCT compound (Tissue-Tek, Sakura Finetek, Tokyo, Japan). Then the tissues were cut into 6 µm thick sections using a cryostat (Leica CM1950, Leica Biosystems, Wetzlar, Germany). DAPI (Roche, Indianapolis, IN, USA) was stained to visualize nuclei of cells for 5 min. The mounted slides allowed fluorescent images to be taken using a Zeiss LSM510 laser scanning confocal microscope (Carl Zeiss, Oberkochen, Germany).

26. In vivo photo-cytotoxic efficacy

Three weeks after the two-site inoculation noted above, the mice (tumor n = 6 per treatment group) were treated with medium containing **CA9-BPS-Cu(II)**, **CA9-BPS** (800 μM, respectively), or PBS (control) via tail vein injection (1 time a week for 3 weeks). 1 h post injection, tumors were subject to 660 nm laser irradiation (2.0 W/cm^2, 10 min; 1200 J/cm^2). Tumor volumes were measured and recorded weekly using a caliper. Tumor volumes were calculated using the expression, tumor volume (mm^3) = 1/2(length x $width^2$). At the end of the experiment, mice were terminated with CO_2 gas and the tumor weights and body weights were measured. No data were excluded from the analyses.

27. Statistical analysis

For all quantitative data, where statistical analysis was performed, statistically meaningful sample sizes were chosen to allow significant differences between negative and positive controls/treatments with or without irradiation to be observed. All *in vitro* studies were conducted in triplicate as three independent experiments. The results are presented along with the standard error of mean. Animal-based studies were performed using n = 3 animals and n = s6 tumors per group. Raw data was normalized using Excel, prior to further analysis. The SAS software (version 8.2, Cary, NC, USA) with one-way and two-way analysis of variance (ANOVA) was applied to determine statistical significance. When the ANOVAs results indicate a significant difference, a two-tailed Student's t-test and a post-hoc Bonferroni test were performed using the SAS software or the Origin software package (version 9.4, Northampton, MA, USA). Significant P-values ($P < 0.05$) are reported as asterisks or different letters in the figures and captions.

28. Supplementary figures

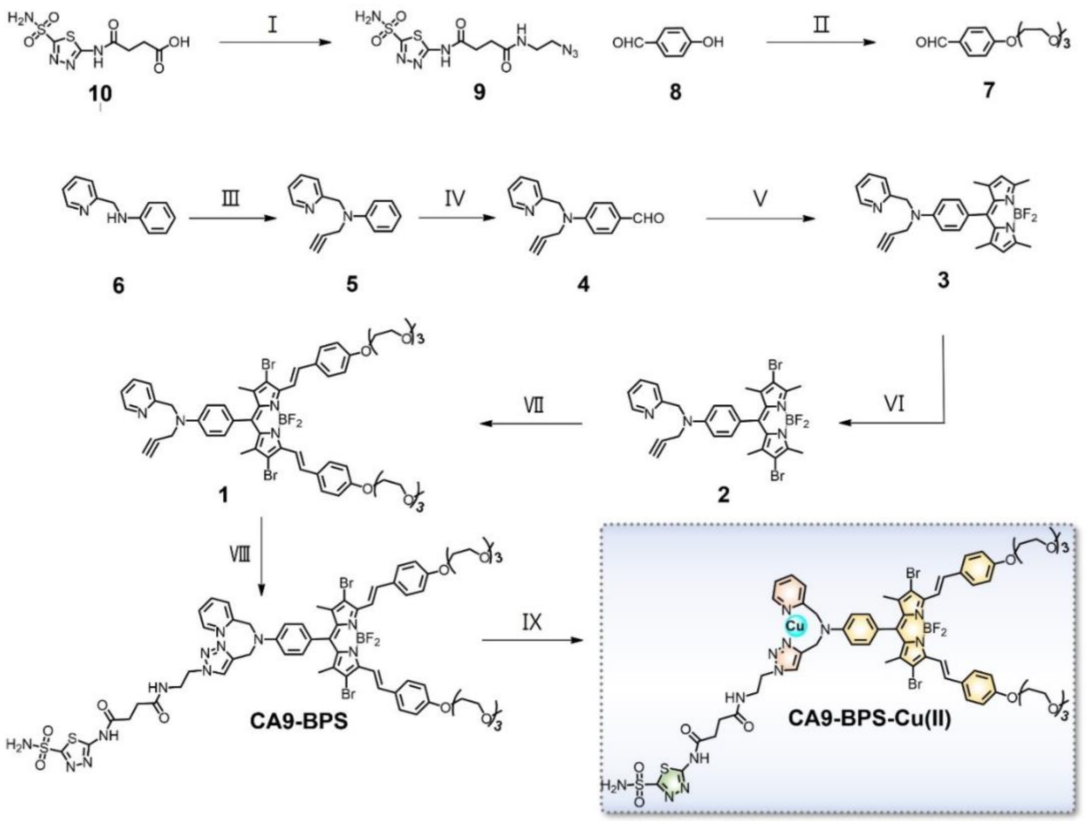

Figure S1. Synthesis of **CA9-BPS-Cu(II)** and **CA9-BPS**. Reagents and conditions: (I) 2-Azidoethylamine, HATU, TEA, DIPEA, DMF, room temperature (rt), 6 h, 62%; (II) $CH_3(OCH_2CH_2)_3OTs$, K_2CO_3, DMF, reflux, 12 h, 90%; (III) propargyl bromide, K_2CO_3, DMF, 50 °C, 12 h, 61%; (IV) DMF, $POCl_3$, 90 °C, 2 h, 80%; (V) 2,4-dimethylpyrrole, trifluoroacetic acid (TFA), CH_2Cl_2, rt, 6 h; *p*-chloranil, $BF_3{\cdot}O(C_2H_5)_2$, TEA, 1.5 h, 45%; (VI) NBS, EtOH, rt, 1 h, 91%; (VII) **7**, $Mg(ClO_4)_2$, glacial acetic acid, piperidine, toluene, reflux, 24 h, 44%; (VIII) **9**, $CuSO_4{\cdot}5H_2O$, sodium ascorbate, DMF/MeOH/H_2O, 6 h, 58%; (IX) $Cu(ClO_4)_2{\cdot}6H_2O$, acetonitrile, rt, 1 h, 80%.

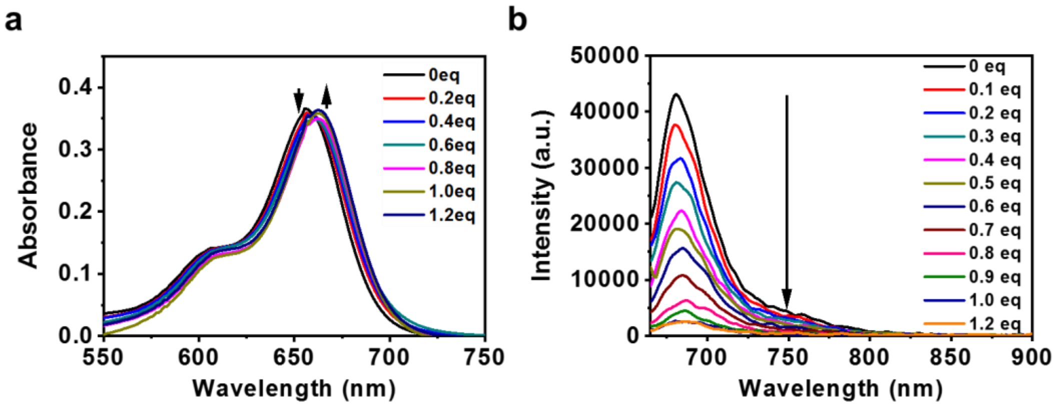

Figure S2. (a) Absorption and (b) fluorescence spectra of **CA9-BPS** (5.0 μM) in ethanol solution recorded upon the addition of various concentrations of Cu(II) (0 – 1.2 equiv.). Excitation at 660 nm. (slit = 2.5/2.5).

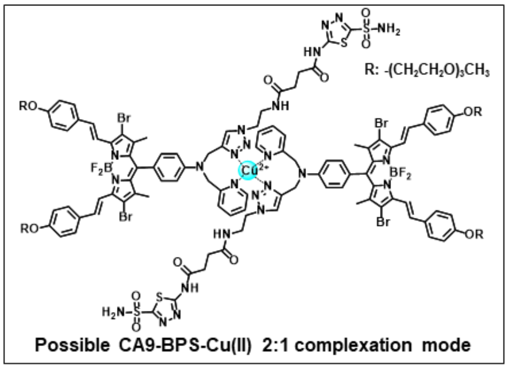

Figure S3. Chemical structure of **CA9-BPS-Cu(II)** showing the proposed 2:1 binding stoichiometry.

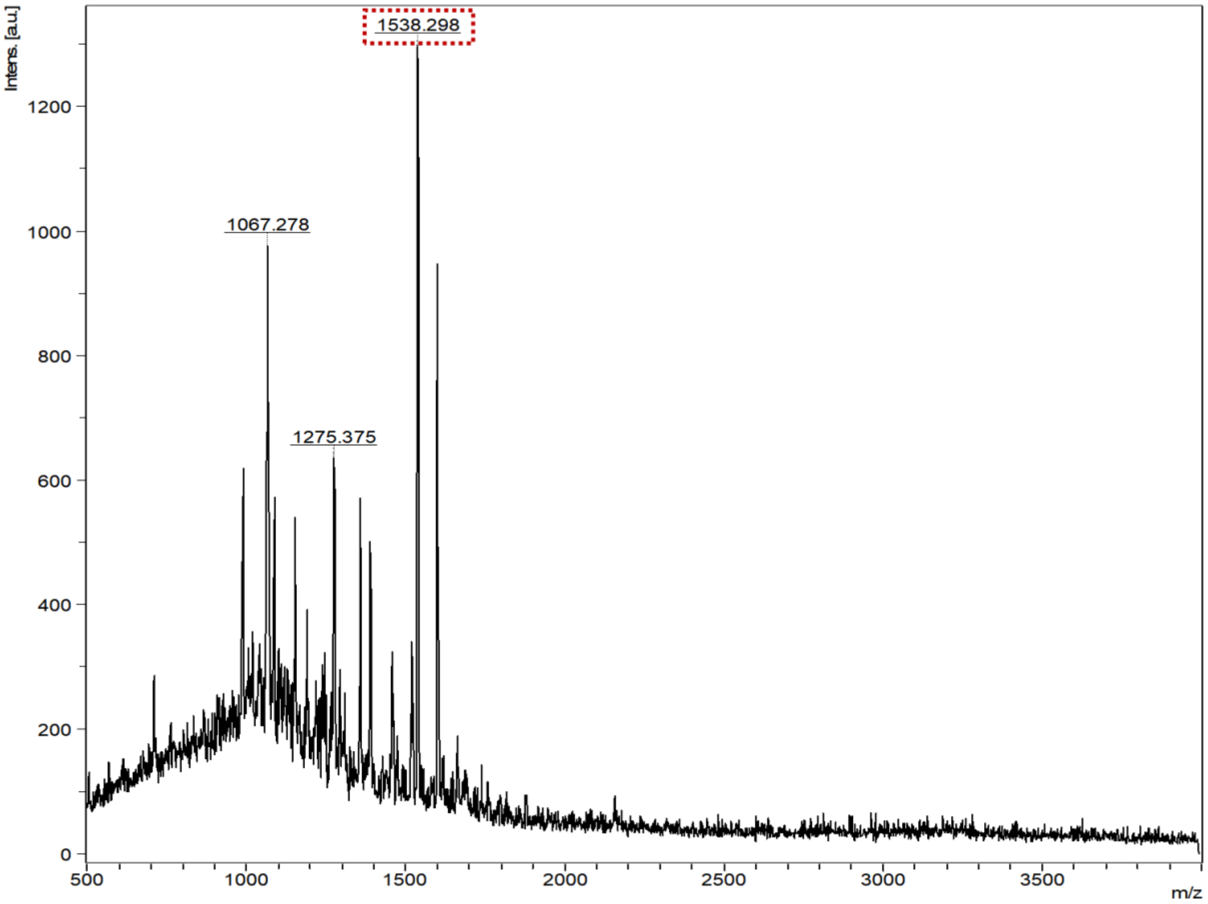

Figure S4. MALDI-TOF/TOF-MS of **CA9-BPS** recorded after treatment with $Cu(ClO_4)_2 \cdot 6H_2O$.

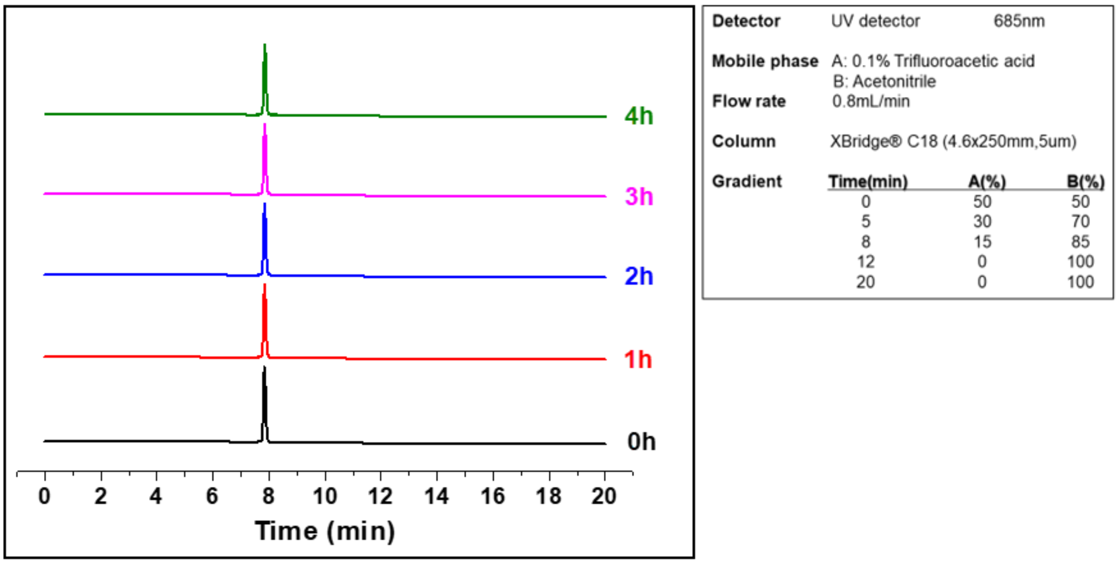

**Detector**	UV detector	685nm	
**Mobile phase**	A: 0.1% Trifluoroacetic acid<br>B: Acetonitrile		
**Flow rate**	0.8mL/min		
**Column**	XBridge® C18 (4.6x250mm,5um)		
**Gradient**	**Time(min)**	**A(%)**	**B(%)**
	0	50	50
	5	30	70
	8	15	85
	12	0	100
	20	0	100

Figure S5. HPLC chromatogram of **CA9-BPS-Cu(II)** in PBS buffered solution (pH 7.4, 10 mM) containing 5% DMSO over a period of 4 hours.

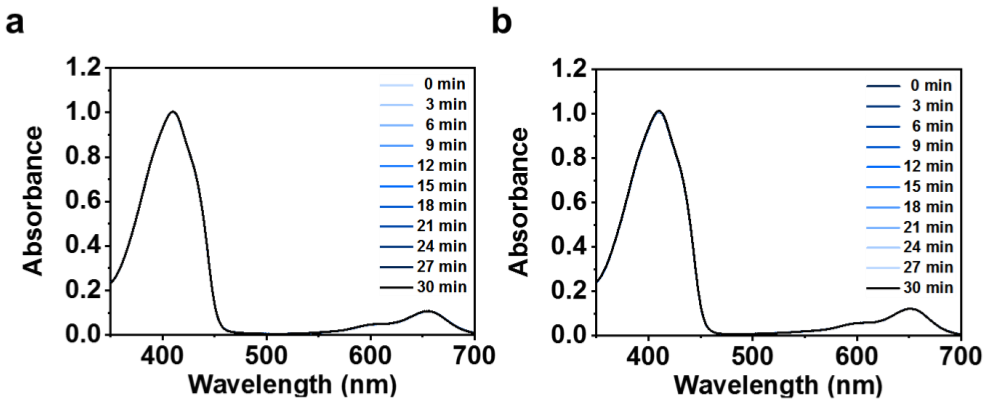

Figure S6. Singlet oxygen generation by **CA9-BPS-Cu(II)** or **CA9-BPS**. Time-dependent absorption spectral changes seen for a 80 μM solution of DPBF containing 1 μM of (a) **CA9-BPS-Cu(II)** or (b) **CA9-BPS** without irradiation.

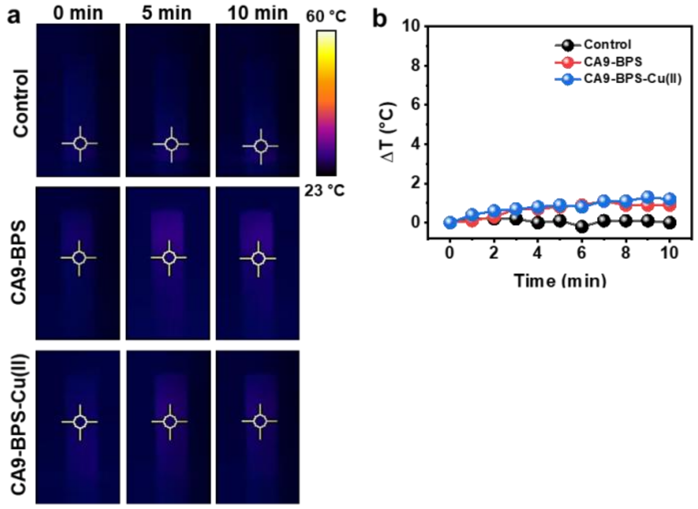

Figure S7. (a) IR thermal images and (b) photothermal heating curves for cell lysate (MDA-MB-231; 20 μg/mL)-PBS solutions containing **CA9-BPS** (0.5 mM) or **CA9-BPS-Cu(II)** (0.5 mM) subject to laser irradiation (2.0 W/cm^2, 10 min; 1200 J/cm^2).

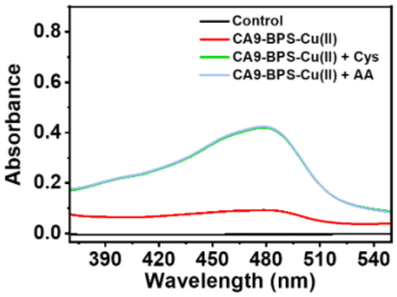

Figure S8. Absorption spectral changes seen for bathocuproinedisulfonic acid disodium salt (BCDS), upon treatment with **CA9-BPS-Cu(II)** in the presence and absence of cysteine (Cys) or ascorbic acid (AA) in MeOH/10 mM PBS (v/v, 1/1) at pH 7.4.

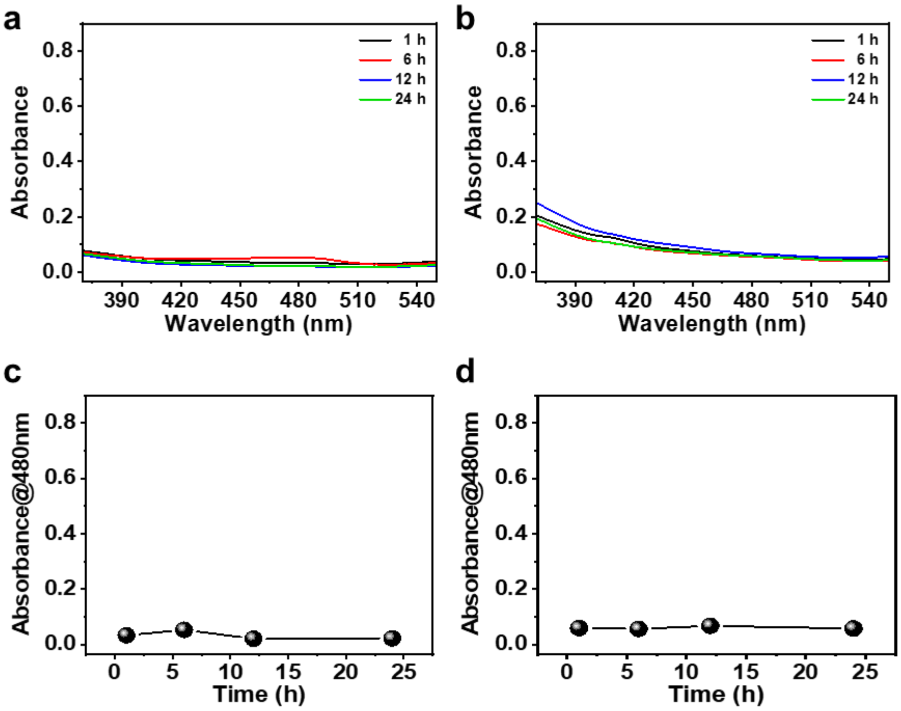

Figure S9. Absorption spectral changes seen for bathocuproinedisulfonic acid disodium salt (BCDS), upon treatment with **CA9-BPS-Cu(II)** preincubated for 1, 6, 12, and 24 h in (a) MeOH/10 mM PBS (v/v, 1/1) at pH 7.4 or (b) RPMI cell culture media (10% FBS, without phenol red). Plot of the absorption change at 480 nm as a function of time (c) seen for (a) or (d) seen for (b).

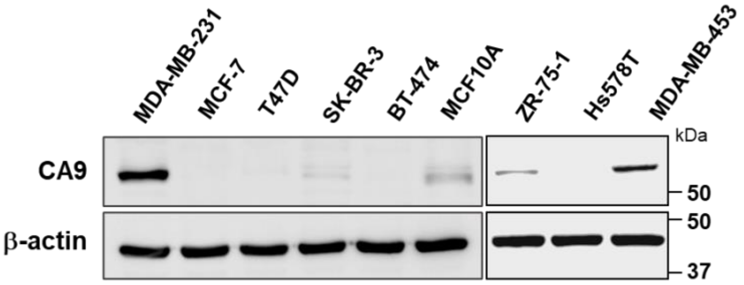

Figure S10. Endogenous expression of CA9 in various breast cancer cell lines (MDA-MB-231, MCF-7, T47D, SK-BR-3, BT-474, ZR-75-1, Hs578T, and MDA-MB-453) and non-malignant breast epithelial cells (MCF10A).

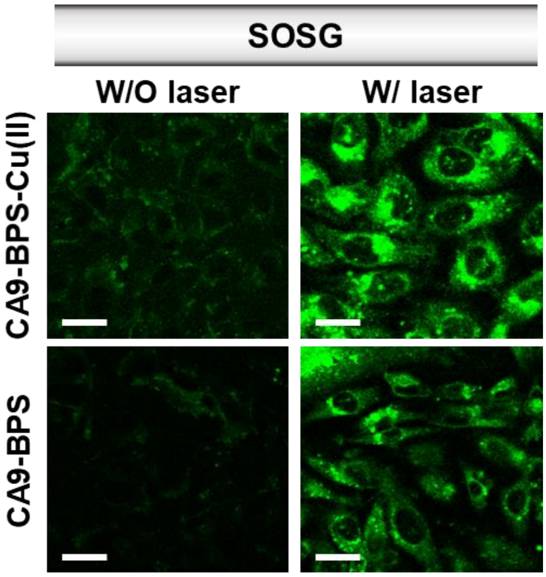

Figure S11. Confocal fluorescence microscopic images of MDA-MB-231 cells incubated with **CA9-BPS**s (5 μM) and 10 μM of Singlet Oxygen Sensor Green (SOSG). The fluorescent images were taken immediately after 660 nm photo-irradiation (100 mW/cm^2, 5 min; 30 J/cm^2). Magnification 200x. Scale bar: 10 μm.

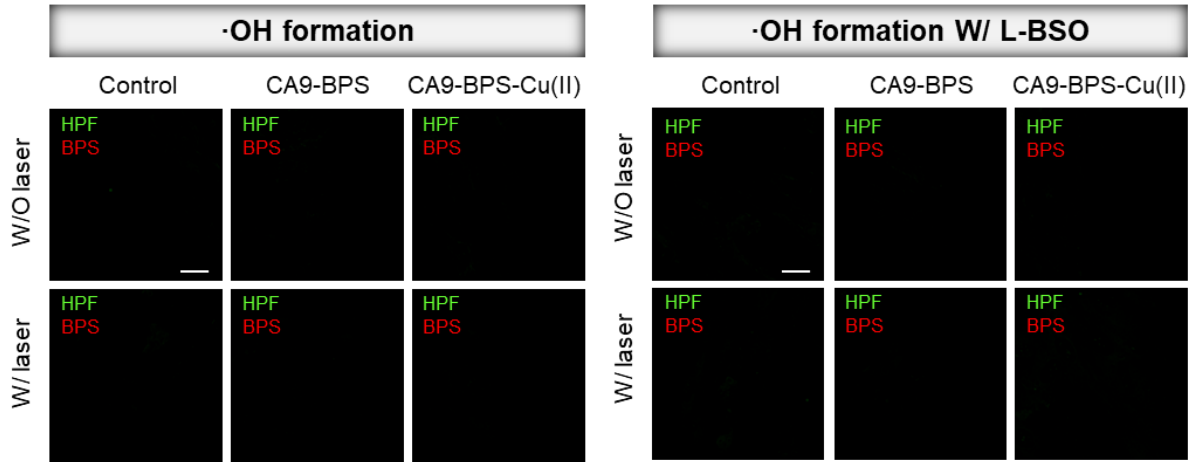

Figure S12. Confocal fluorescence microscopic images of BJ cells incubated with the two **CA9-BPS**s considered in the present study (5 µM, respectively) and hydroxyphenyl fluorescein (HPF; 10 µM). The images were recorded with or without 660 nm light irradiation (100 mW/cm^2, 5 min; 30 J/cm^2). Cells were subject to 100 µM L-buthionine sulfoximine (L-BSO) pretreatment 24 h before the **CA9-BPS**s were administered. Magnification: 120x. Scale bars: 20 µm.

Comp.	Control	$CuCl_2$	CA9-BPS	CA9-BPS-Cu(II)
Cu conc. (ppm)	< 0.1	< 0.1	< 0.1	38.6

Figure S13. Intracellular copper accumulation: MDA-MB-231 cells were incubated with 20 µM of the indicated compounds for 24 h and washed with PBS and the copper concentrations in the cell lysates were measured using ICP-MS.

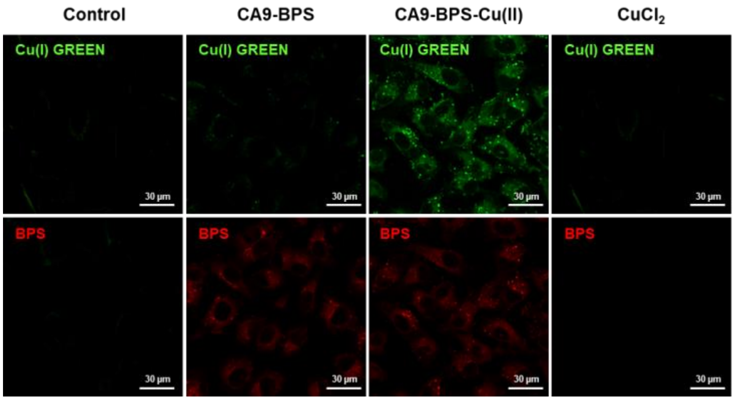

Figure S14. Confocal fluorescence microscopic images of MDA-MB-231 cells incubated with **CA9-BPSs** (20 μM), $CuCl_2$ (20 μM), and 5 μM copperGREEN dye (Cu(I) tracer) for 30 min. Scale bar = 30 μm.

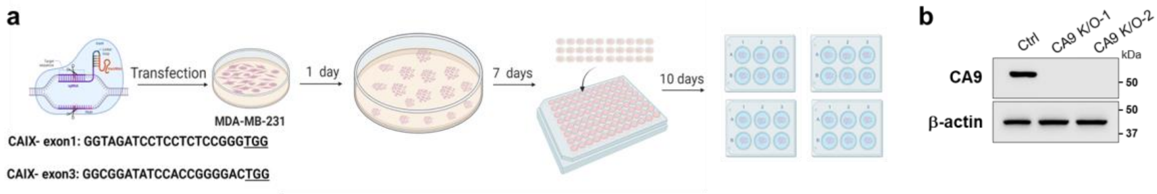

Figure S15. (a) Schematic illustration of CRISPR-Cas9 knockout process. (b) Western blot analysis of CA9 expression in CA9 CRISPR-Cas9 knockout MDA-MB-231 cells.

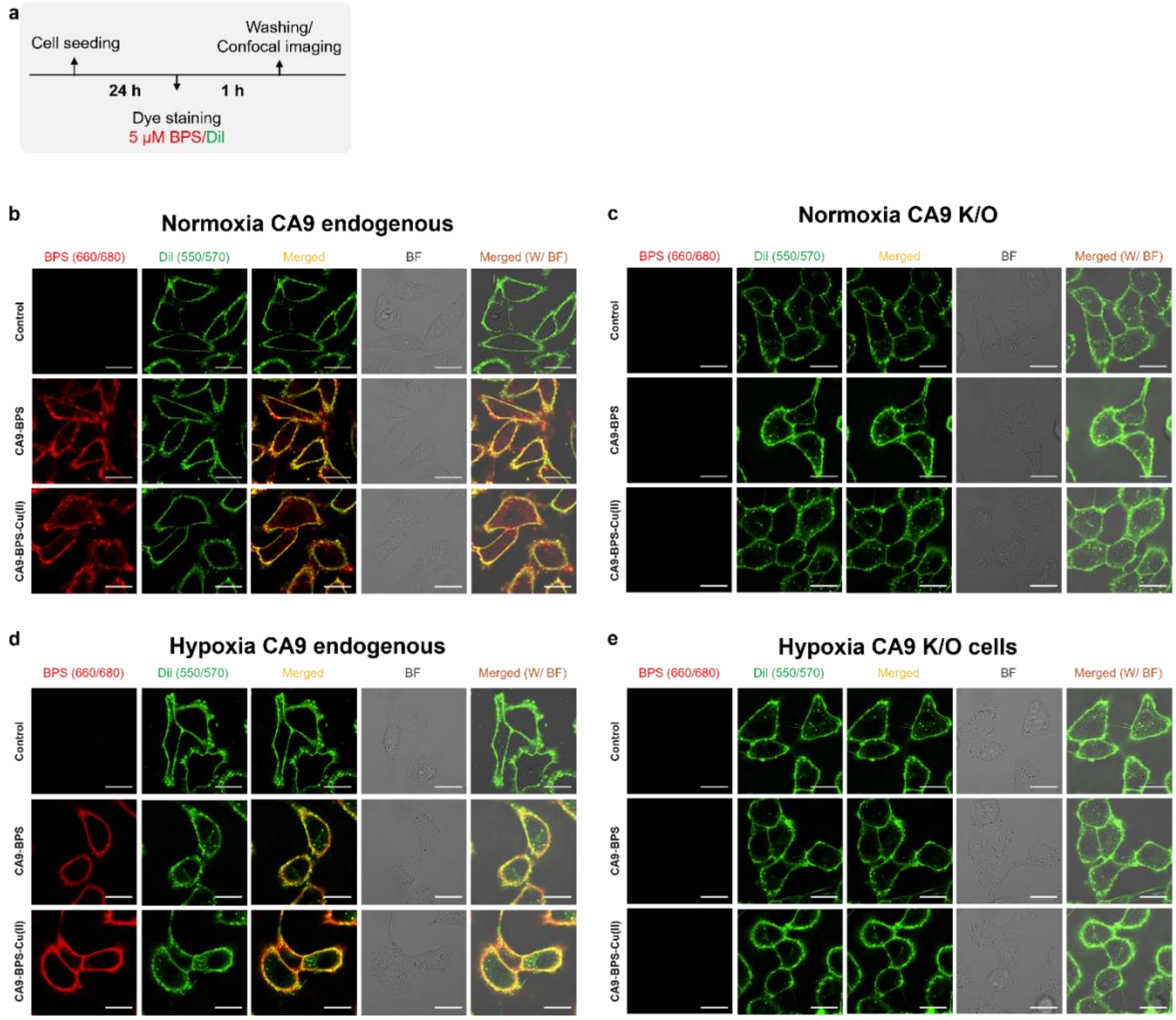

Figure S16. (a) Schematic diagram of confocal fluorescence microscopic imaging procedure. Confocal fluorescence microscopic images of (b) normoxic MDA-MB-231, (c) normoxic CA9 knockout MDA-MB-231, (d) hypoxic MDA-MB-231, and (e) hypoxic CA9 knockout MDA-MB-231 cells incubated with 5 μM **CA9-BPS**s (red) and 5 μM Dil (green; membrane staining dye) for 1h. Magnification: 120x. Scale bars: 20 μm.

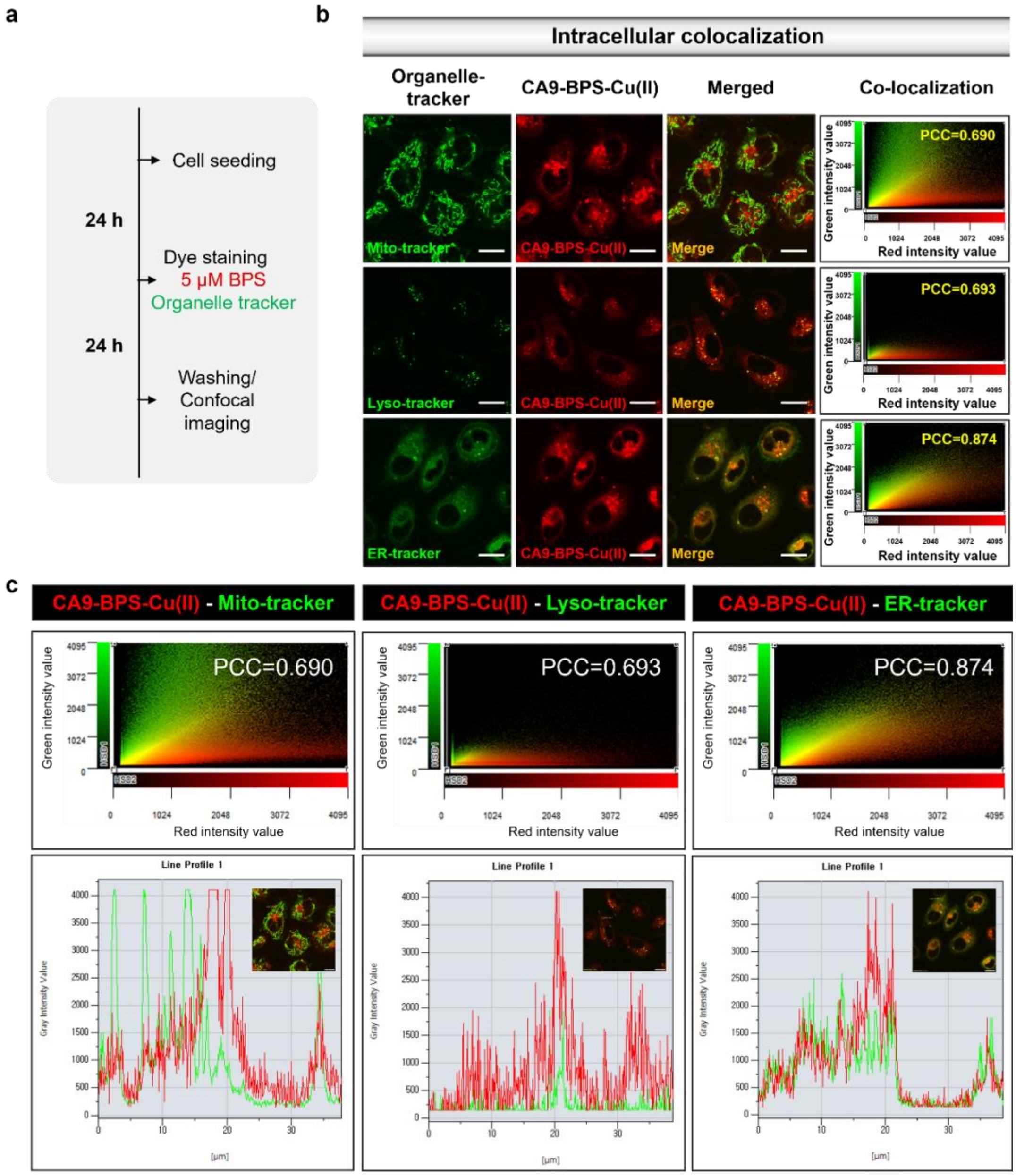

Figure S17. (a) Schematic diagram of confocal fluorescence microscopic imaging procedure. (b) Intracellular co-localization studies of **CA9-BPS-Cu(II)**: MDA-MB-231 cells were incubated with 5 μM of **CA9-BPS-Cu(II)** for 24 h. After being washed with PBS, Mito-, ER-, and Lyso-Trackers® (green) were added and the cells were incubated for an additional 30 min before the fluorescent images were recorded. Magnification: 142x. Scale bars: 20 μm. (c) Scatter plot and colocalization analysis of the fluorescence signal from **CA9-BPS-Cu(II)** (red) and Mito-, Lyso-, and ER-Trackers® (green).

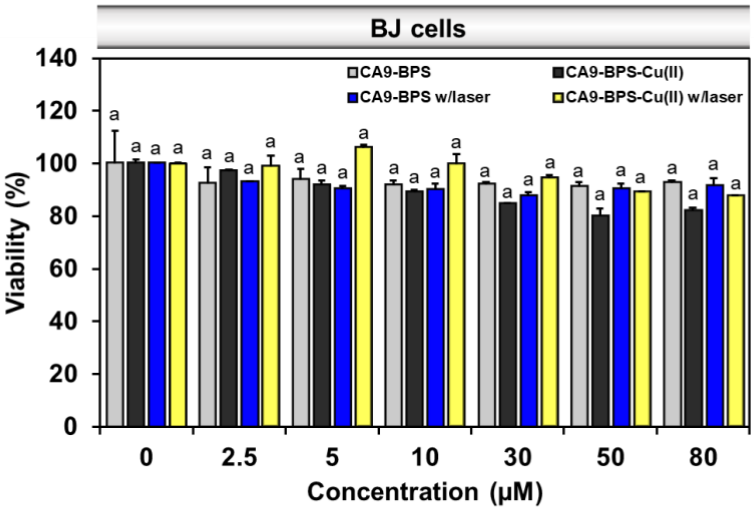

Figure S18. *In vitro* cytotoxicity of **CA9-BPS-Cu(II)** or **CA9-BPS** as tested in the BJ cells (human normal fibroblasts). Cells were irradiated with a 660 nm light source (100 mW/cm^2, 5 min; 30 J/cm^2). MTT assays were performed 24 h after irradiation. Data are presented as the mean, while the error bars indicate the standard deviation from the mean (n = 3). Statistical significance was determined using a two-way ANOVA test with a post-hoc Bonferroni test. Where present, different letters signify datasets that are statistically distinct ($p < 0.05$).

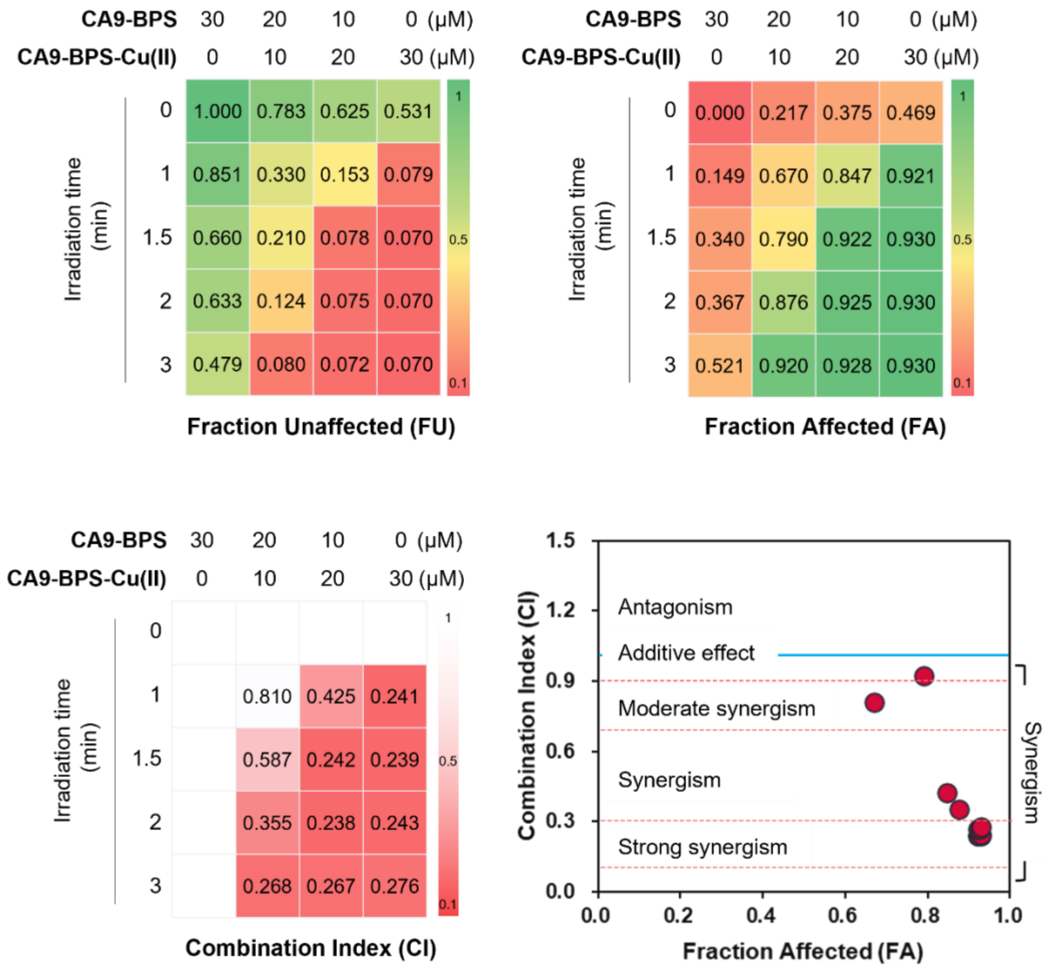

Figure S19. Plot of combination index for PDT and CDT effects using **CA9-BPS-Cu(II)**. PDT and CDT-induced cytotoxicity was investigated upon treatment with an increasing dose of each modality as a function of light energy density and copper concentration, respectively. Fraction Unaffected (FU): percent of cell viability/100. Fraction Affected (FA): 1-FU. The combination index (CI) was calculated using Eqn. S2.

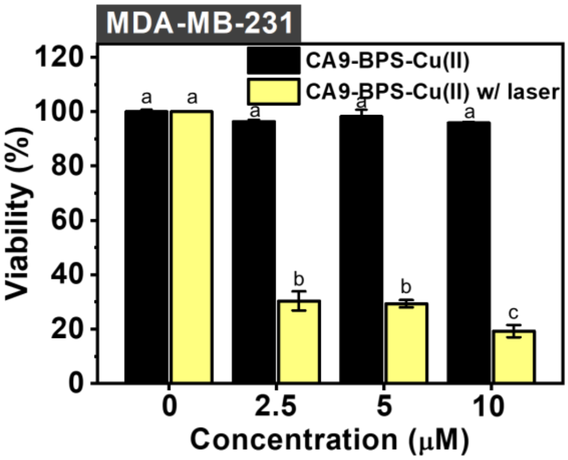

Figure S20. Cytotoxicity of **CA9-BPS-Cu(II)** in MDA-MB-231 cells determined in the absence and after irradiating at 660 nm (100 mW/cm^2, 5 min; 30 J/cm^2). The cells were pre-treated with NEM (1 mM) for 30 min. Data are presented as the mean, while the error bars indicate the standard deviation from the mean (n = 3). Statistical significance was determined using a two-way ANOVA test with a post-hoc Bonferroni test. Where present, different letters (a,b,c) signify datasets that are statistically distinct ($p < 0.05$).

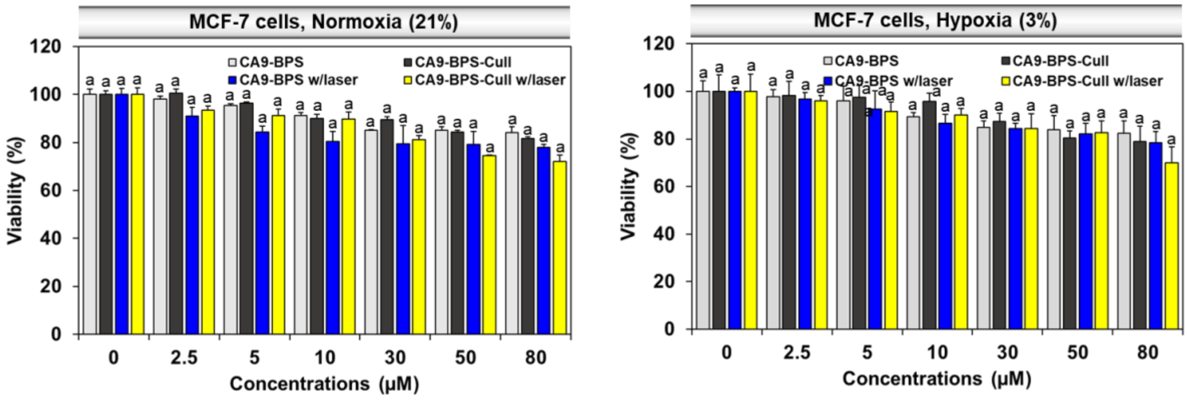

Figure S21. Cytotoxicity of **CA9-BPS-Cu(II)** and **CA9-BPS** as tested under normoxia (21%) and hypoxia (3%) in MCF-7 cells. Data are presented as the mean, while the error bars indicate the standard deviation from the mean (n = 3). Statistical significance was determined using a one-way ANOVA test with a post-hoc Bonferroni test. The identical letter (a) is used to signify datasets that are not statistically distinct ($p > 0.05$).

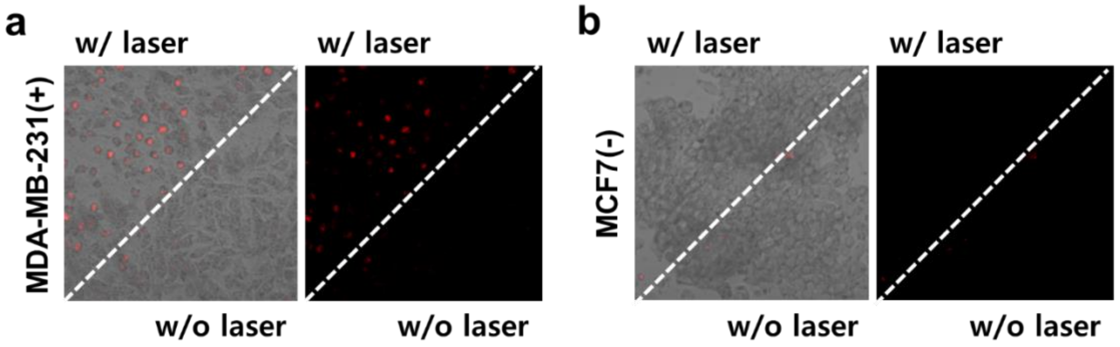

Figure S22. Confocal fluorescence images of (a) MDA-MB-231 or (b) MCF-7 cells recorded after incubation with **CA9-BPS-Cu(II)** (5 μM) for 24 h followed by irradiating with 660 nm laser light (2 W/cm^2, 5 min; 600 J/cm^2). Dead cells appear red as the result of propidium iodide (PI) staining. Magnification 100x.

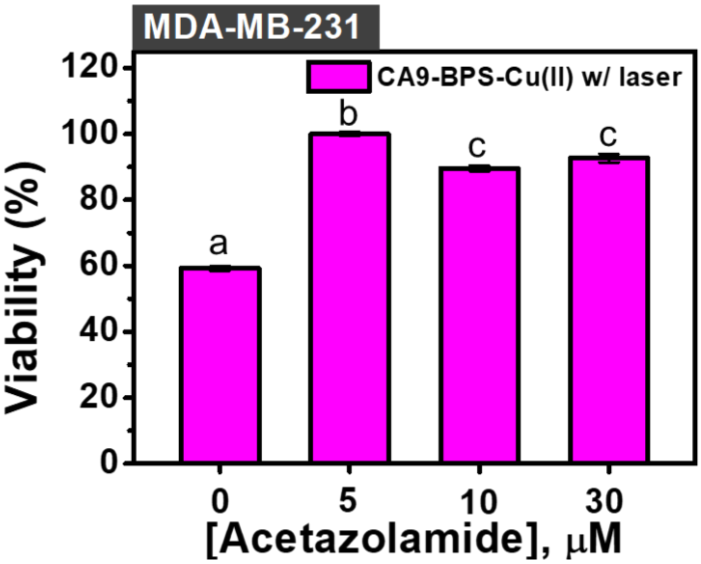

Figure S23. Cytotoxicity of **CA9-BPS-Cu(II)** (5 μM) determined in MDA-MB-231 cells following pre-treatment with different concentrations of acetazolamide (0, 5, 10, and 30 μM, respectively) prior to irradiation with 660 nm light (100 mW/cm^2, 5 min; 30 J/cm^2). As a control, MDA-MB-231 cells were treated with 0 μM of acetazolamide and 1% DMSO instead of **CA9-BPS-Cu(II)**. Data are presented as the mean, while the error bars indicate the standard deviation from the mean (n = 3). Statistical significance was determined using a one-way ANOVA test with a post-hoc Bonferroni test. Different letters (a,b,c), where present, signify data that are statistically distinct ($p < 0.05$).

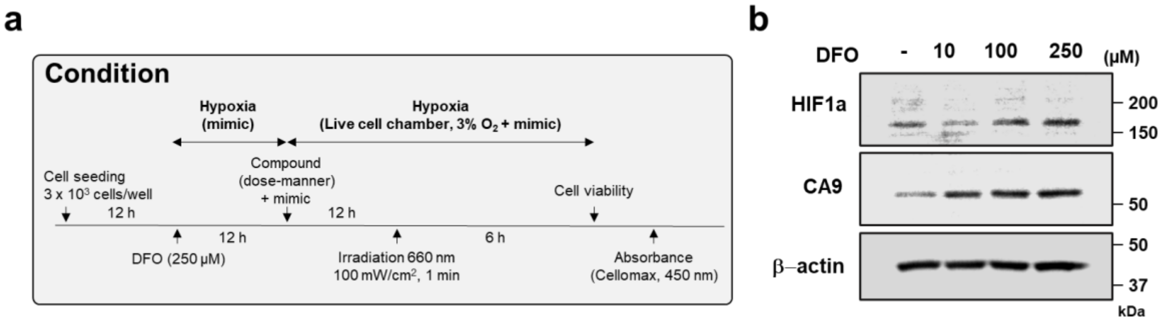

Figure S24. (a) Experimental design for evaluating the cytotoxicity of **CA9-BPS**s of this study under hypoxic conditions. (b) Expression of HIF-1alpha and CA9 after treating MDA-MB-231 cells with DFO (0 μM, 10 μM, 100 μM and 250 μM) for 12 h.

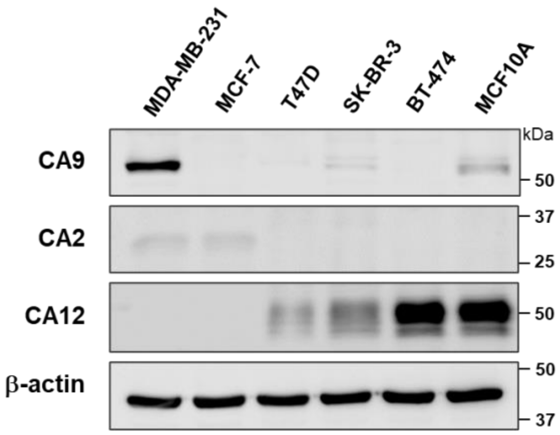

Figure S25. Endogenous expression of CA9, CA2, and CA12 in various breast cancer cells (MDA-MB-231, MCF7, T47D, SK-BR-3, BT474) and normal breast cells (MCF10A).

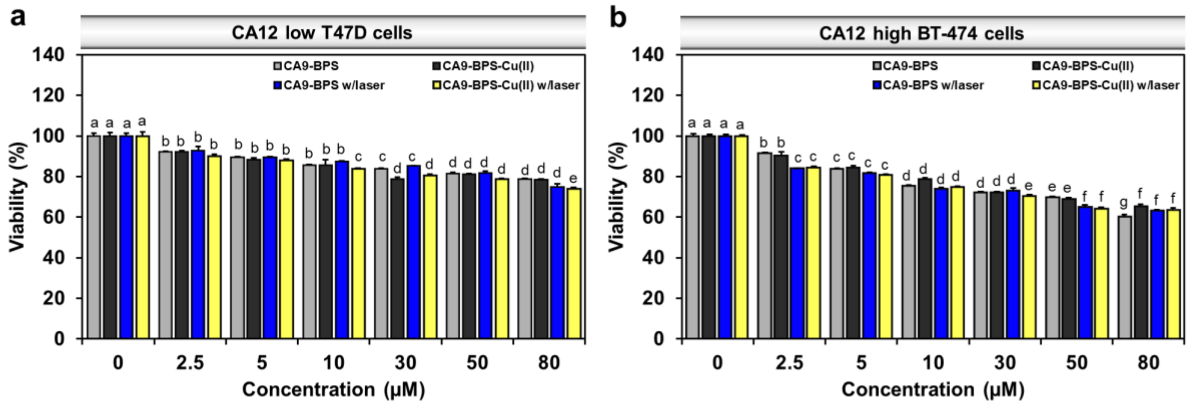

Figure S26. *In vitro* cytotoxicity of **CA9-BPS-Cu(II)** and **CA9-BPS** as tested in (a) CA12-low T47D and (b) CA12-high BT-474 breast cancer cells. The cells were irradiated with a 660 nm LED lamp (100 mW/cm^2, 5 min; 30 J/cm^2). MTT assays were performed 24 h after irradiation. Data are presented as the mean, while the error bars indicate the standard errors from the mean (n = 3). Statistical significance was determined using a two-way ANOVA test with a post-hoc Bonferroni test. Different letters signify data that are statistically distinct ($p < 0.05$).

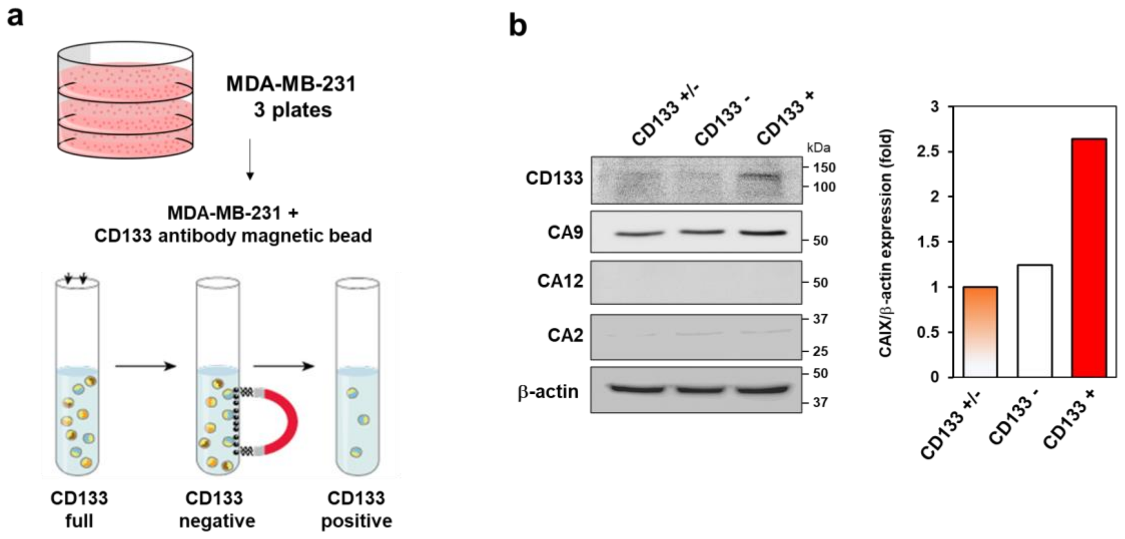

Figure S27. (a) Graphical representation of magnetic-activated cell sorting (MACS) of MDA-MB-231 cells based on the CD133 levels. (b) Western blot analysis of CA9, CA12, and CA2 expression of MACS unsorted cells as well as the MACS sorted cells: CD133-negative and CD133-positive. The expression of CA9 was quantified by normalization to the b-actin expression.

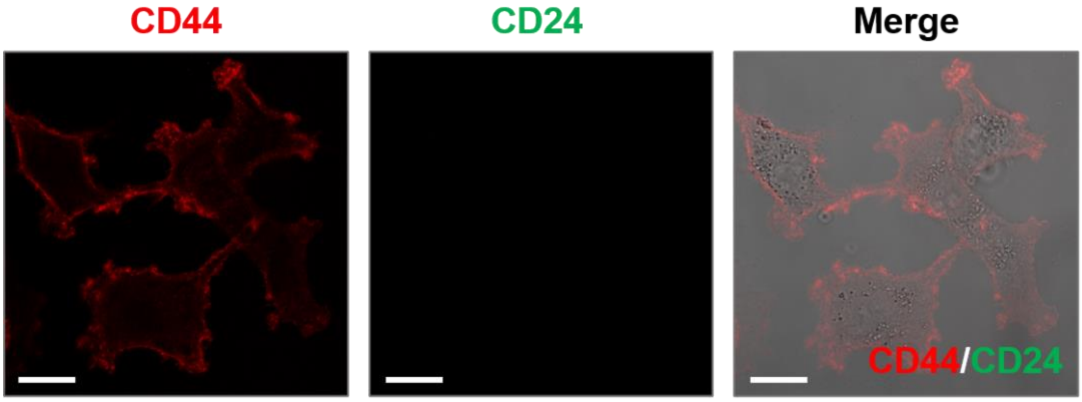

Figure S28. Immunofluorescence images showing the expression of CD44 and CD24 in CD44-positive and CD24-negative MDA-MB-231 cells sorted by MACS. Cells were labeled with anti-CD44-PE (phycoerythrin) and anti-CD24–FITC (fluorescein). Scale bar: 20 μm.

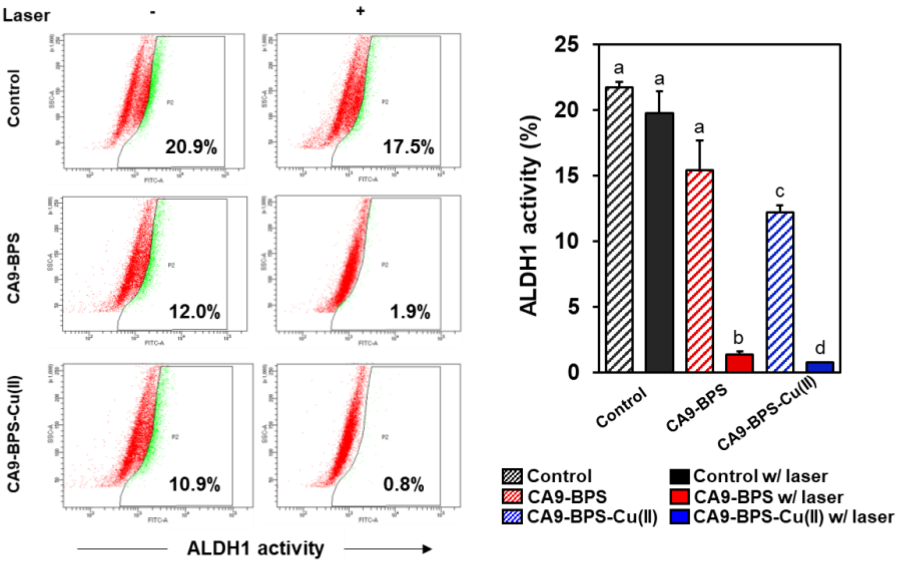

Figure S29. FACS analysis of ALDH1 activity in CD44-positive/CD24-negative MDA-MB-231 cells treated with **CA9-BPS-Cu(II)** and **CA9-BPS**. The cells were irradiated with a 660 nm LED lamp (100 mW/cm^2, 5 min; 30 J/cm^2) and the aldefluor assay was performed 1 h after irradiation. Data are presented as the mean, while the error bars indicate the standard deviation from the mean (n = 3). Statistical significance was determined using a two-way ANOVA test with a post-hoc Bonferroni test. Different letters signify data that are statistically distinct ($p < 0.05$).

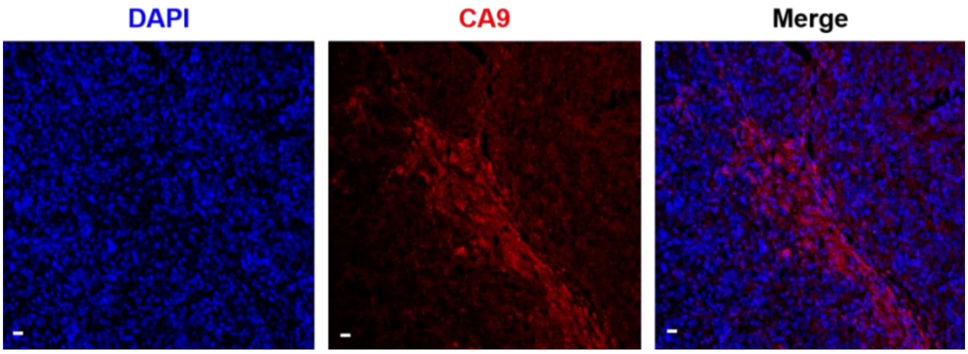

Figure S30. CA9 expression in the xenograft tumor tissue. Immunohistochemical (IHC) staining with anti-CA9 antibody. DAPI (blue as nuclei count staining) and anti-CA9 (red). The tumor tissues were dissected from mice inoculated with MDA-MB-231 cells. Scale bar: 100 μm.

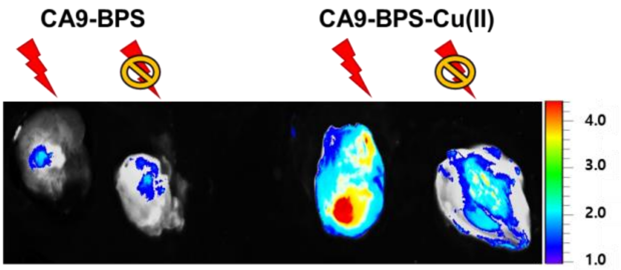

Figure S31. Generation of hydroxyl radicals in tumor tissues from mice treated with **CA9-BPS** or **CA9-BPS-Cu(II)**. Fluorescent intensity of HPF in *ex vivo* tumor tissues was detected from xenografted mice administrated either **CA9-BPS** or **CA9-BPS-Cu(II)** via tail vein injection (500 μM). HPF (100 μl of a 5 mM solution per tumor) was directly injected into the tumors, were irradiated with a 660 nm laser (2.0 W/cm^2, 10 min; 1200 J/cm^2). The mice were terminated with CO_2 gas and the tumors were dissected and subject to fluorescent imaging.

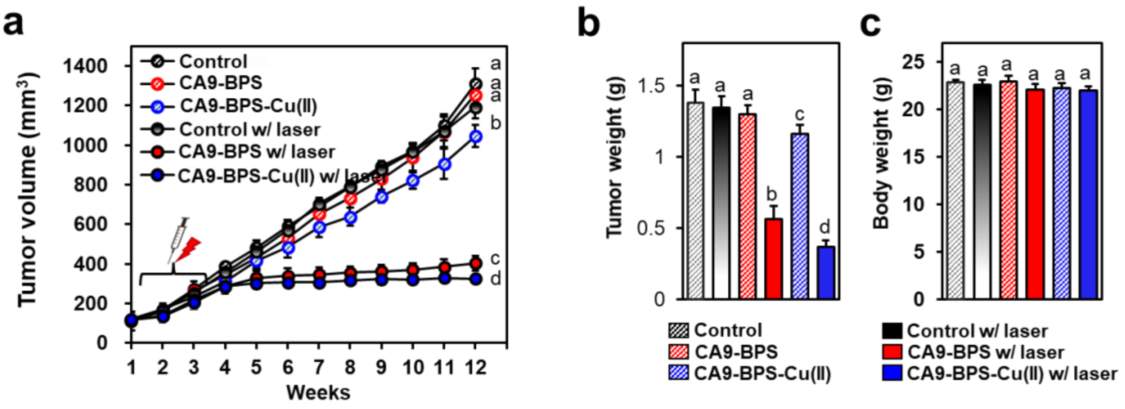

Figure S32. *In vivo* photo-cytotoxic effects of **CA9-BPS**s in xenograft mice models based on the MDA-MB-453 cell lines. (a) Tumor volume, (b) tumor weight, and (c) body weight of the mice in the **CA9-BPS-Cu(II)** or **CA9-BPS** groups with or without PDT treatment (2.0 W/cm^2, 10 min; 1200 J/cm^2). Data are presented as the mean, while the error bars indicate the standard deviation from the mean (n = 6). Statistical significance was determined using one-way and two-way ANOVA tests with associated post-hoc Bonferroni tests. Different letters signify data that are statistically distinct ($p < 0.05$).

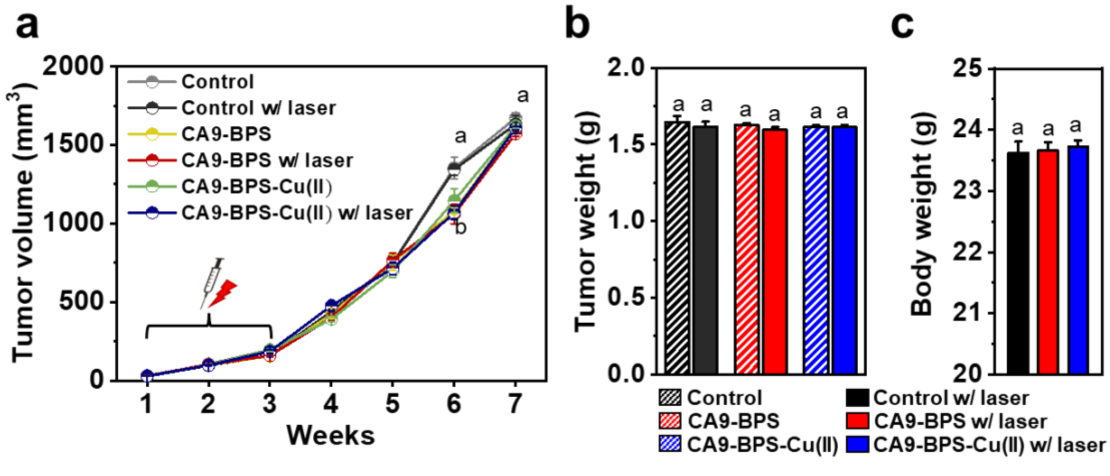

Figure S33. *In vivo* photo-cytotoxic effects of **CA9-BPS**s in xenograft mice models having T47D cell lines. (a) Tumor volume, (b) tumor weight, and **(c)** body weight of the mice in the **CA9-BPS-Cu(II)** or **CA9-BPS** groups with or without PDT treatment (2.0 W/cm^2, 10 min; 1200 J/cm^2). Data are presented as the mean, while the error bars indicate the standard deviation from the mean (n = 6). Statistical significance was determined using one-way and two-way ANOVA tests with associated post-hoc Bonferroni tests. Different letters signify data that are statistically distinct ($p < 0.05$).

29. ^{1}H NMR and ^{13}C NMR spectra

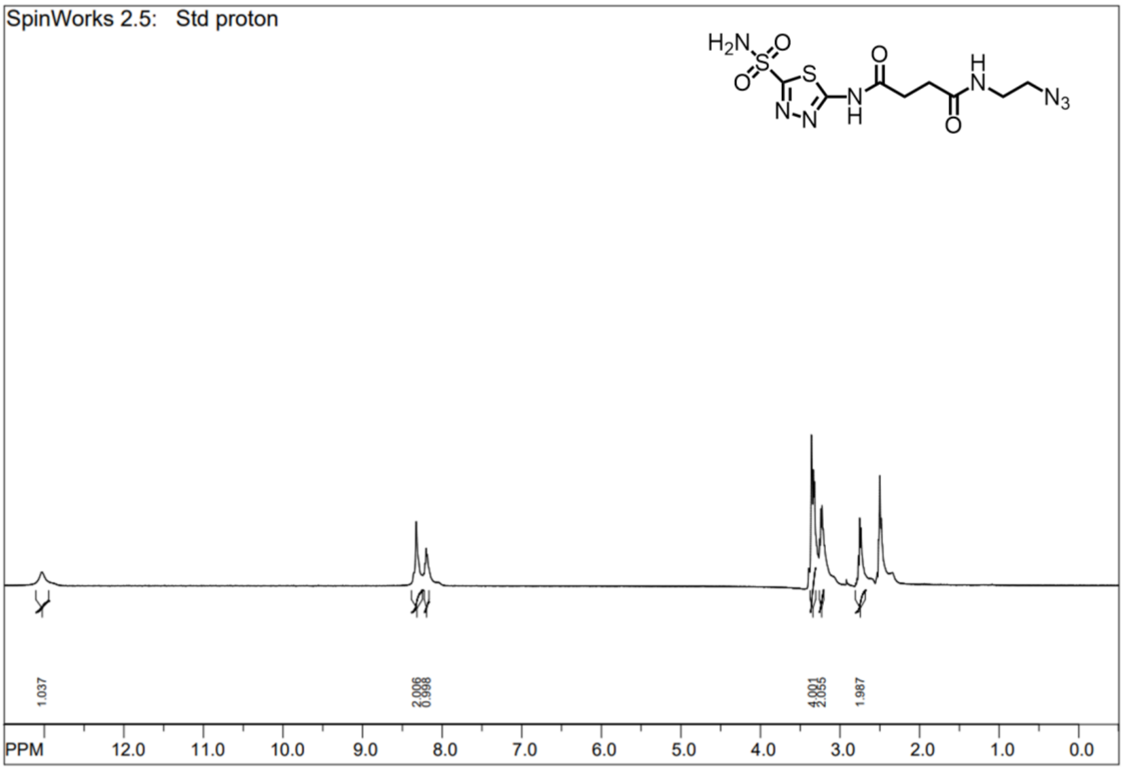

Figure S34. ^{1}H NMR spectrum (400 MHz) of **9** recorded in DMSO-d_6.

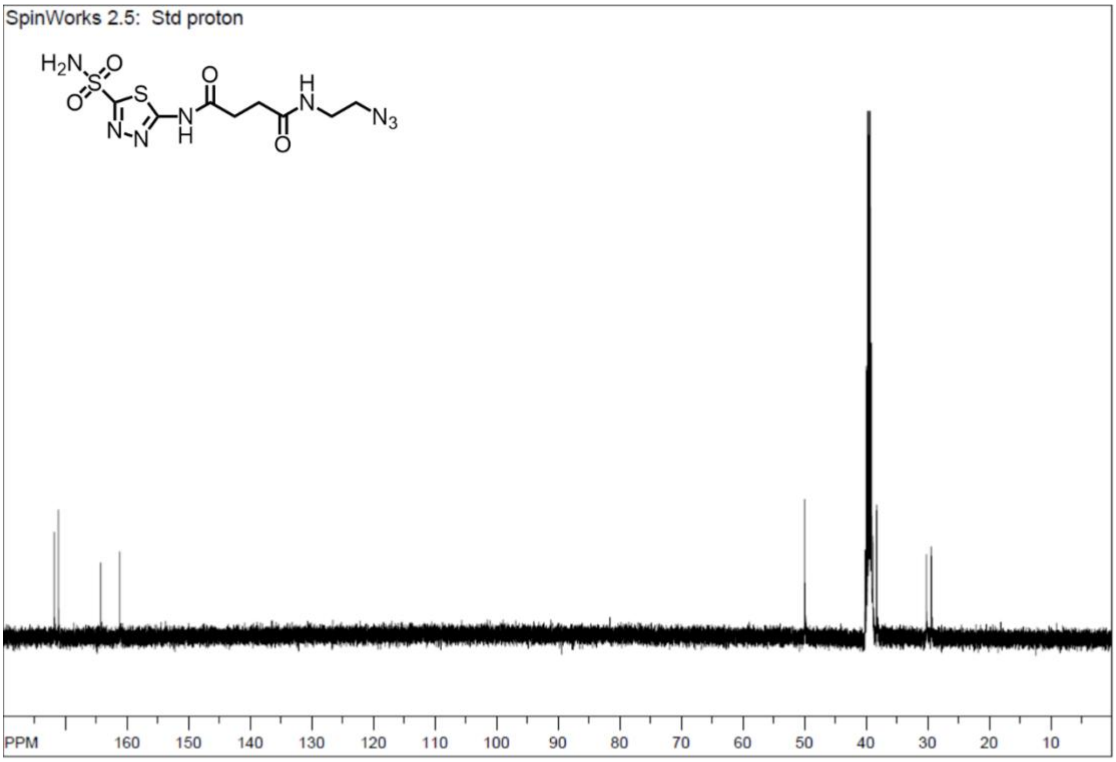

Figure S35. ^{13}C NMR spectrum (100 MHz) of **9** recorded in DMSO-d_6.

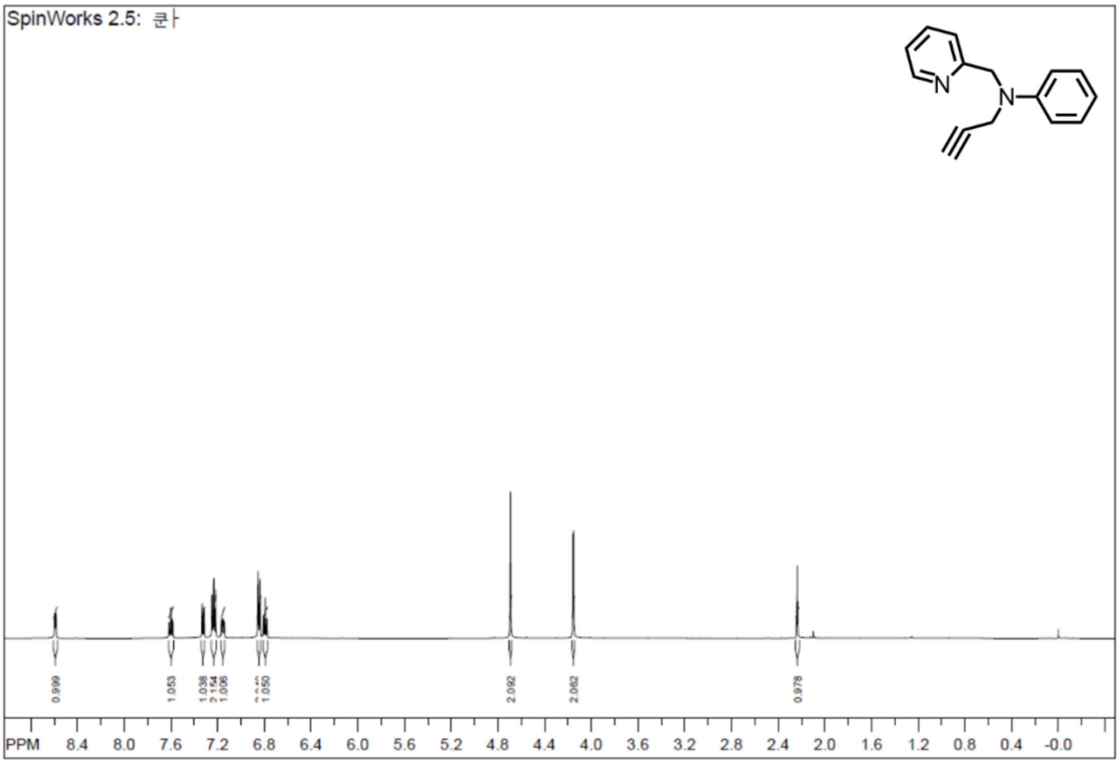

Figure S36. ^{1}H NMR spectrum (400 MHz) of **5** recorded in $CDCl_3$.

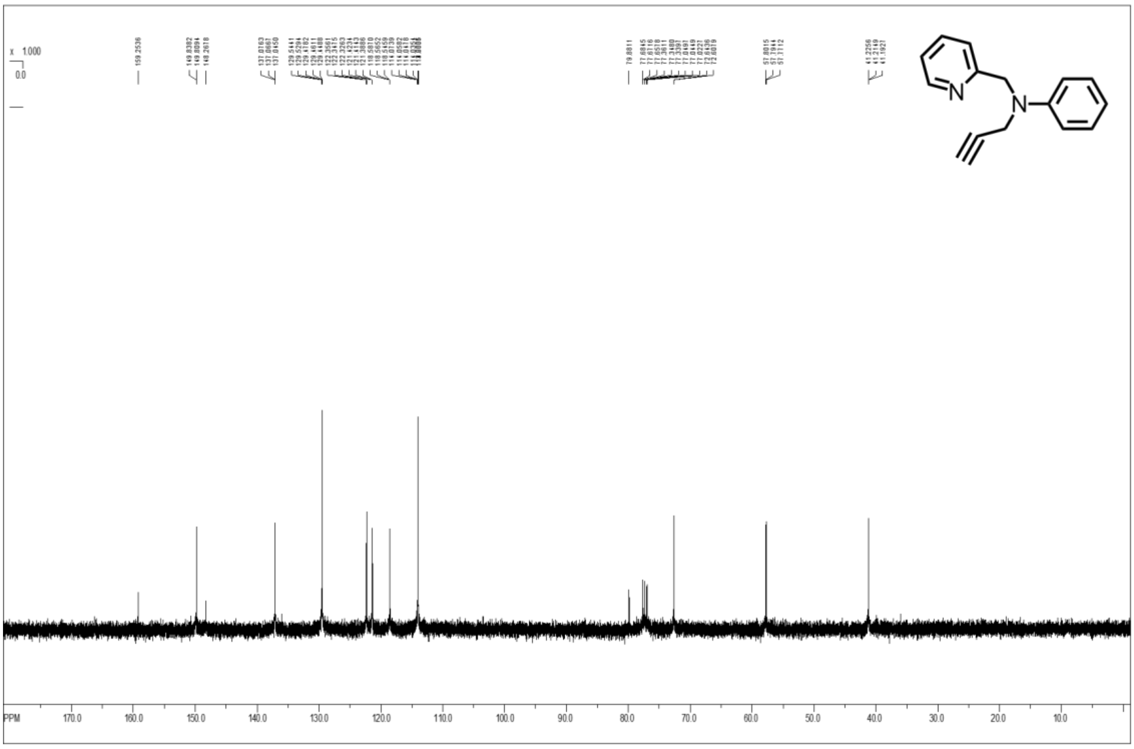

Figure S37. ^{13}C NMR spectrum (100 MHz) of **5** recorded in $CDCl_3$.

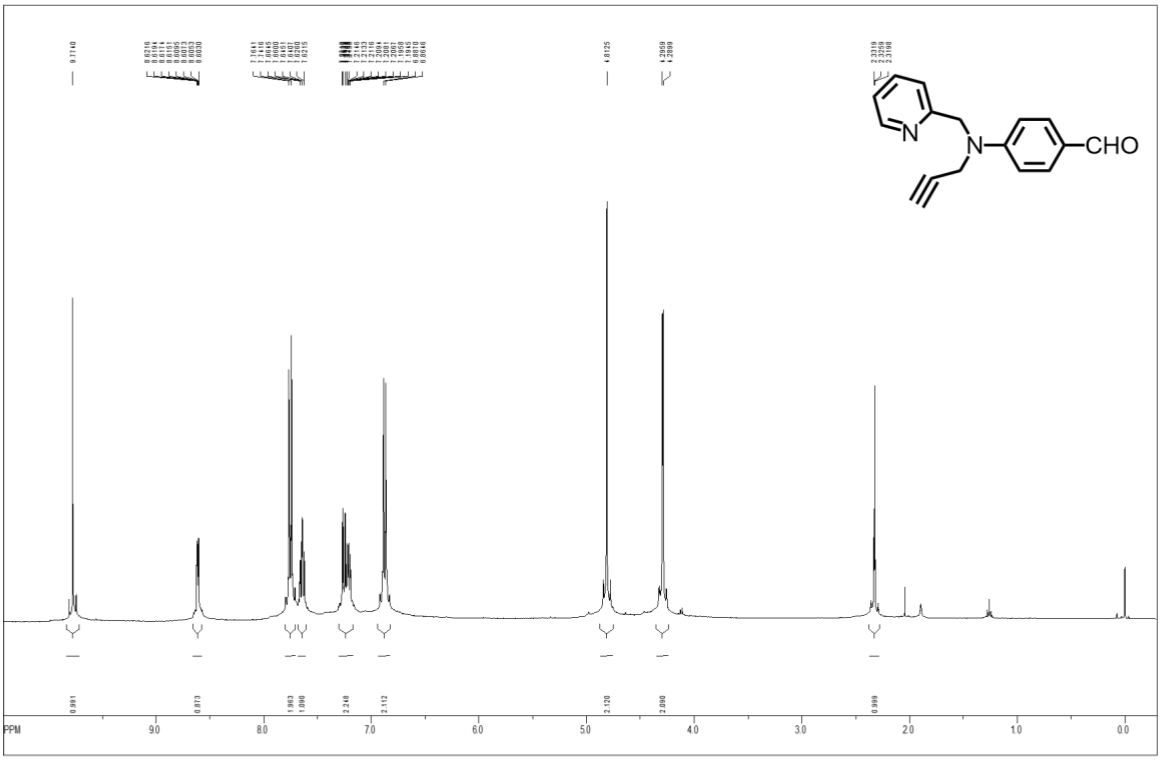

Figure S38. ^{1}H NMR spectrum (400 MHz) of **4** recorded in $CDCl_3$.

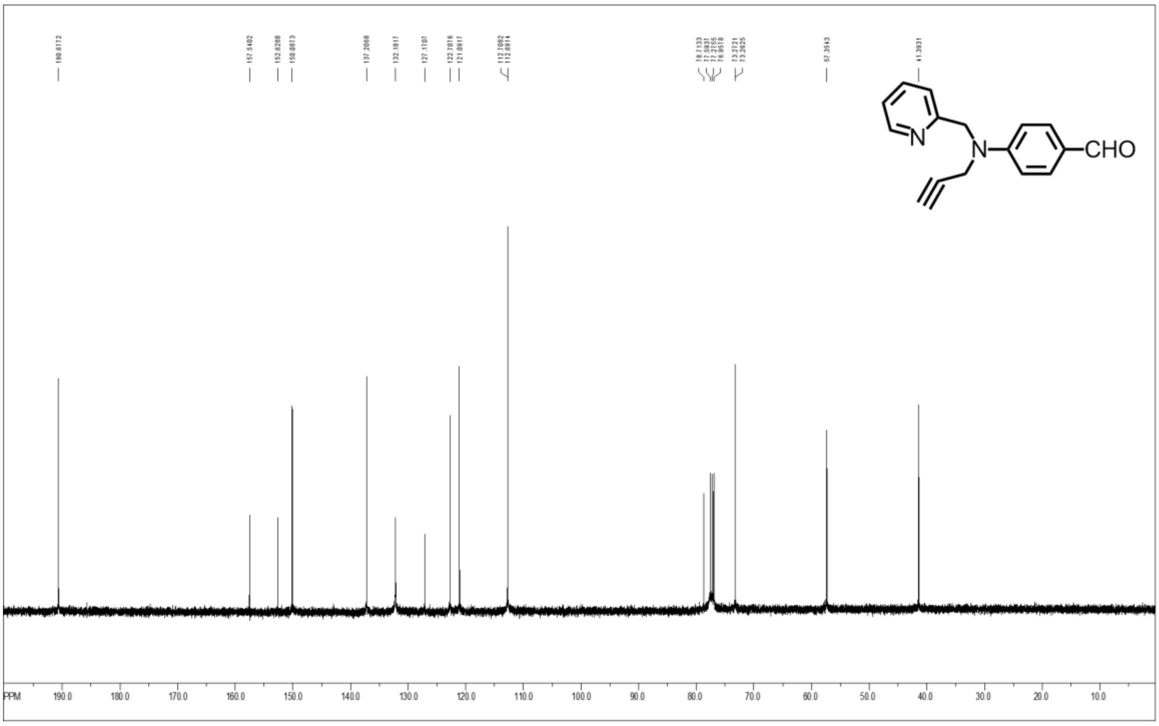

Figure S39. ^{13}C NMR spectrum (100 MHz) of **4** recorded in $CDCl_3$.

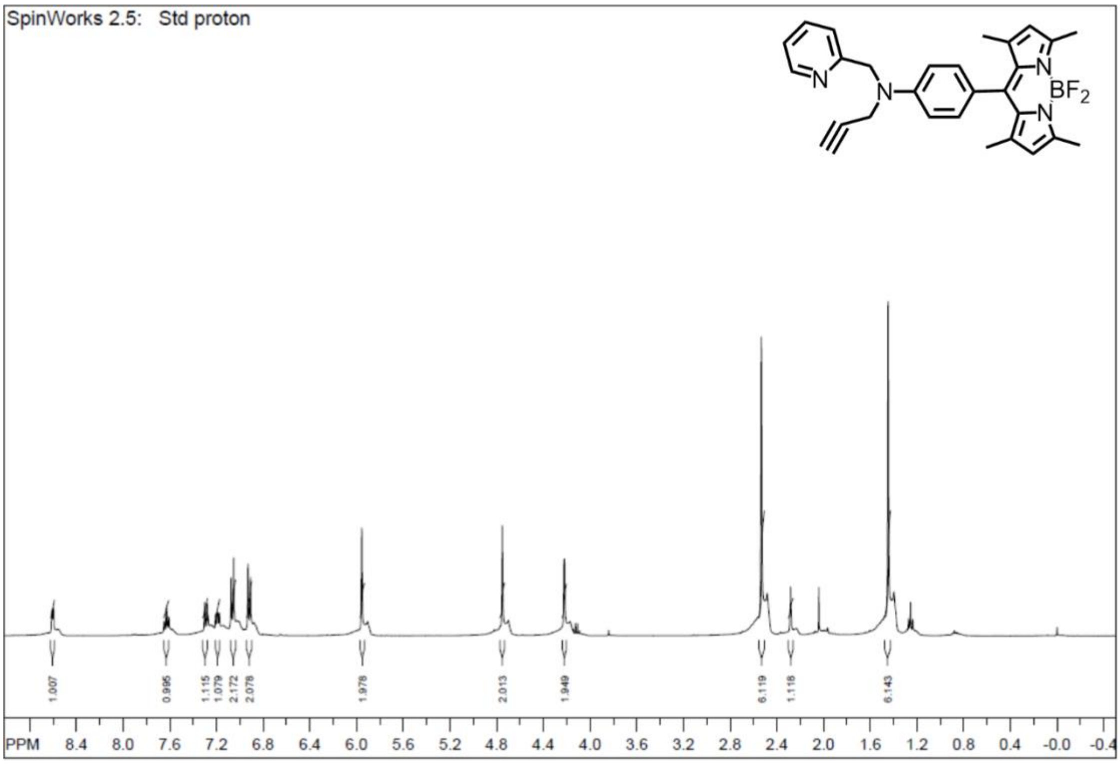

Figure S40. ^{1}H NMR spectrum (400 MHz) of **3** recorded in $CDCl_3$.

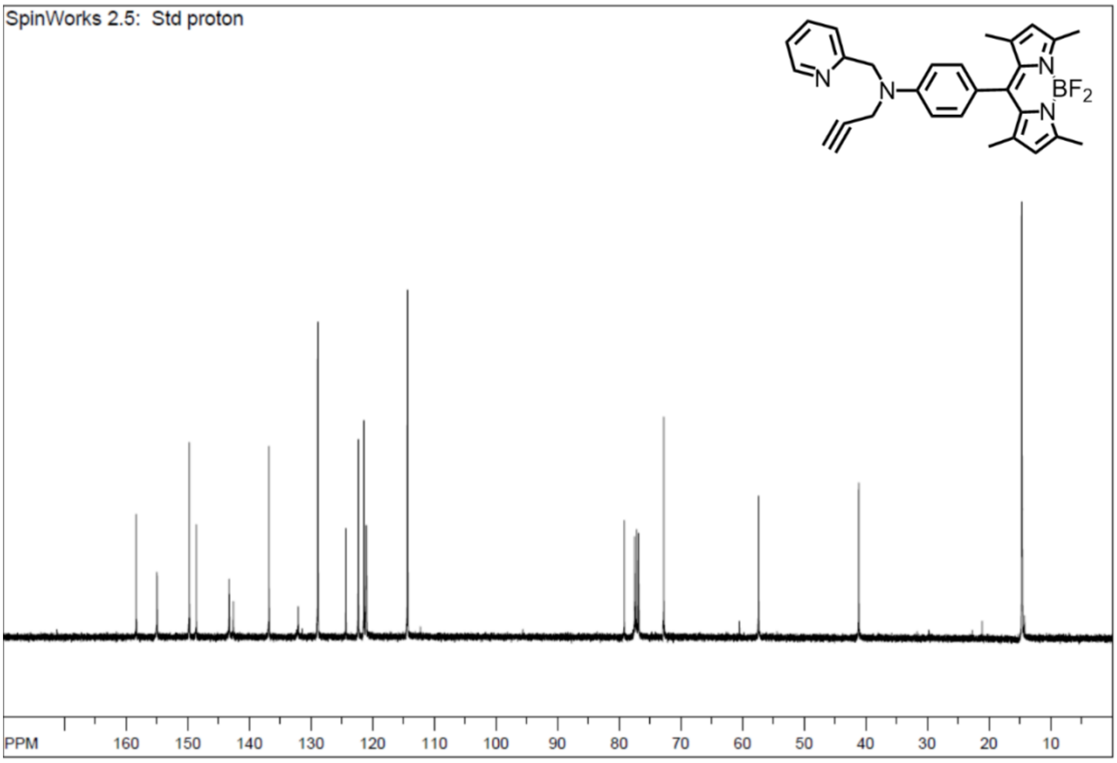

Figure S41. ^{13}C NMR spectrum (100 MHz) of **3** recorded in $CDCl_3$.

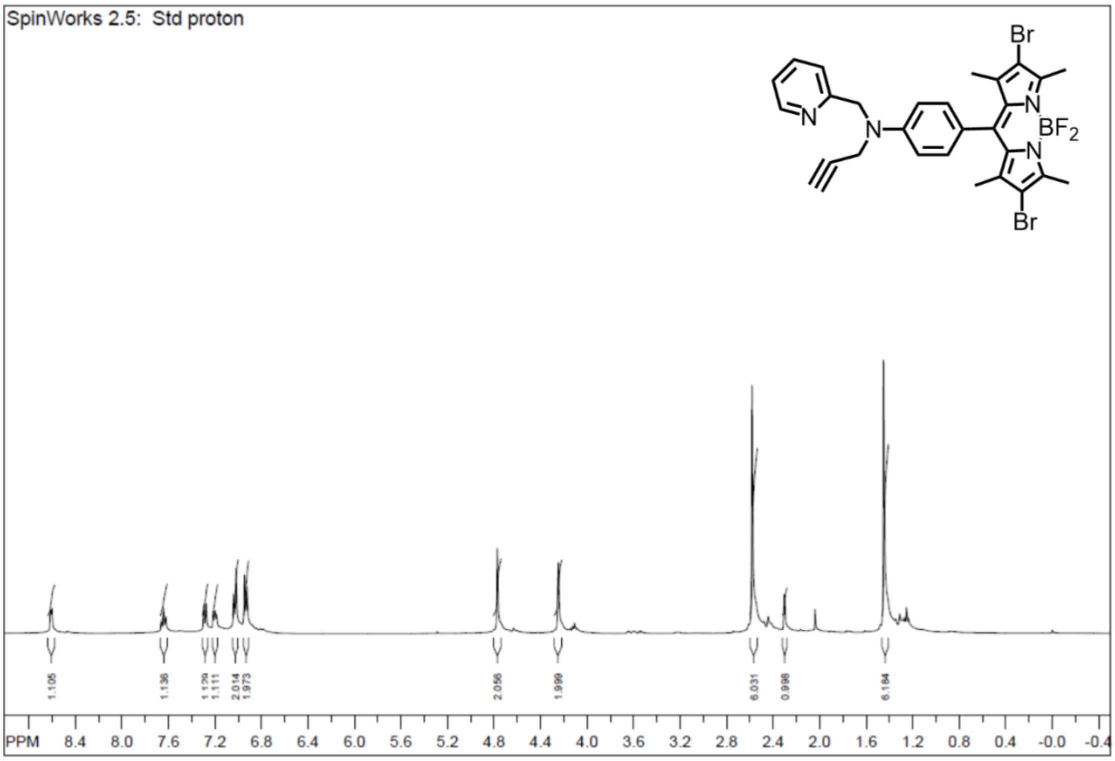

Figure S42. ^{1}H NMR spectrum (400 MHz) of **2** recorded in $CDCl_3$.

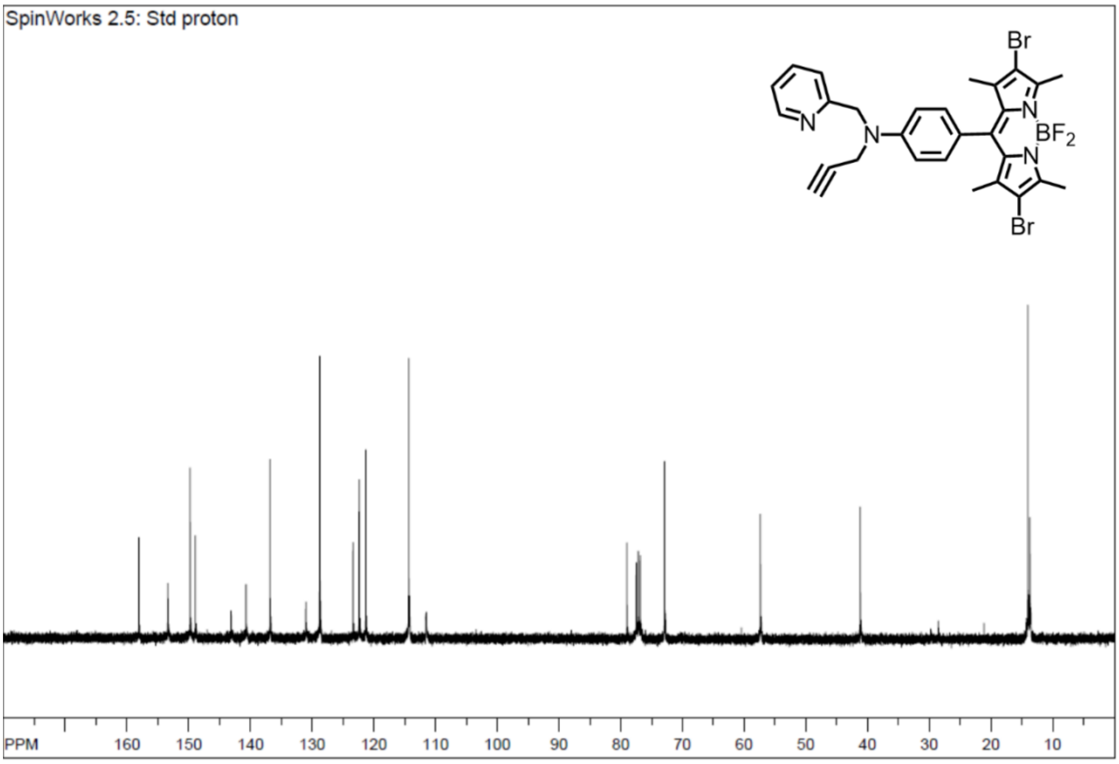

Figure S43. ^{13}C NMR spectrum (100 MHz) of **2** recorded in $CDCl_3$.

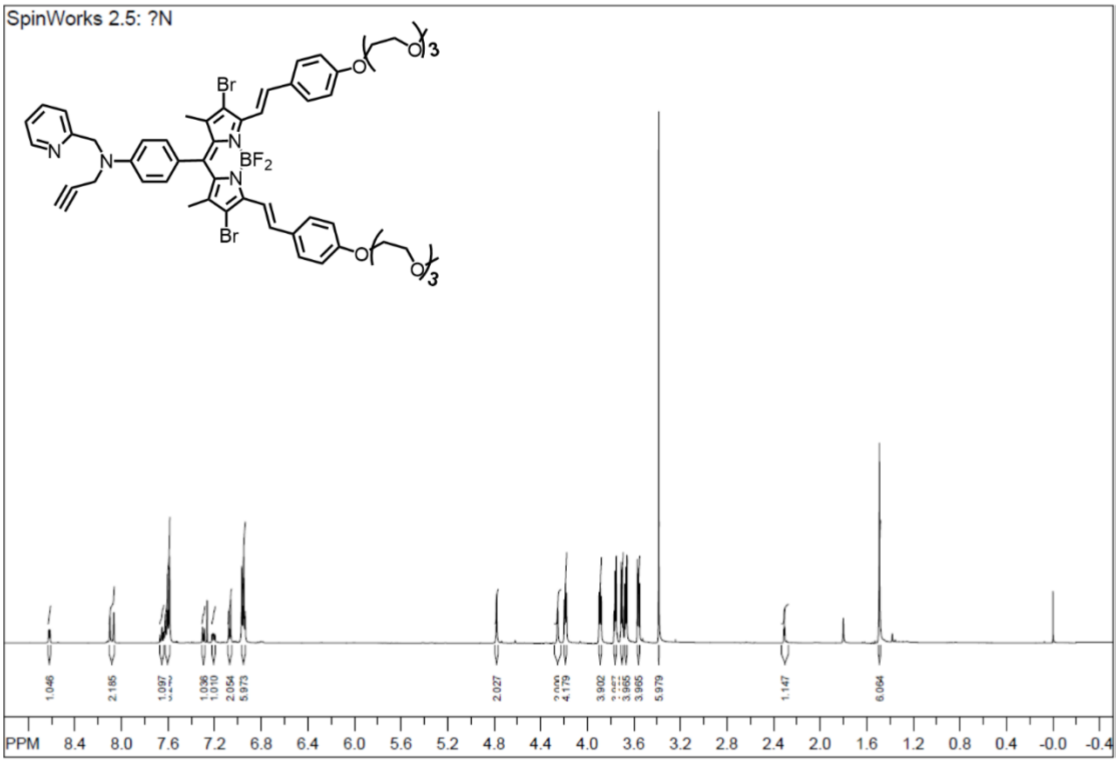

Figure S44. ^{1}H NMR spectrum (400 MHz) of **1** recorded in $CDCl_3$.

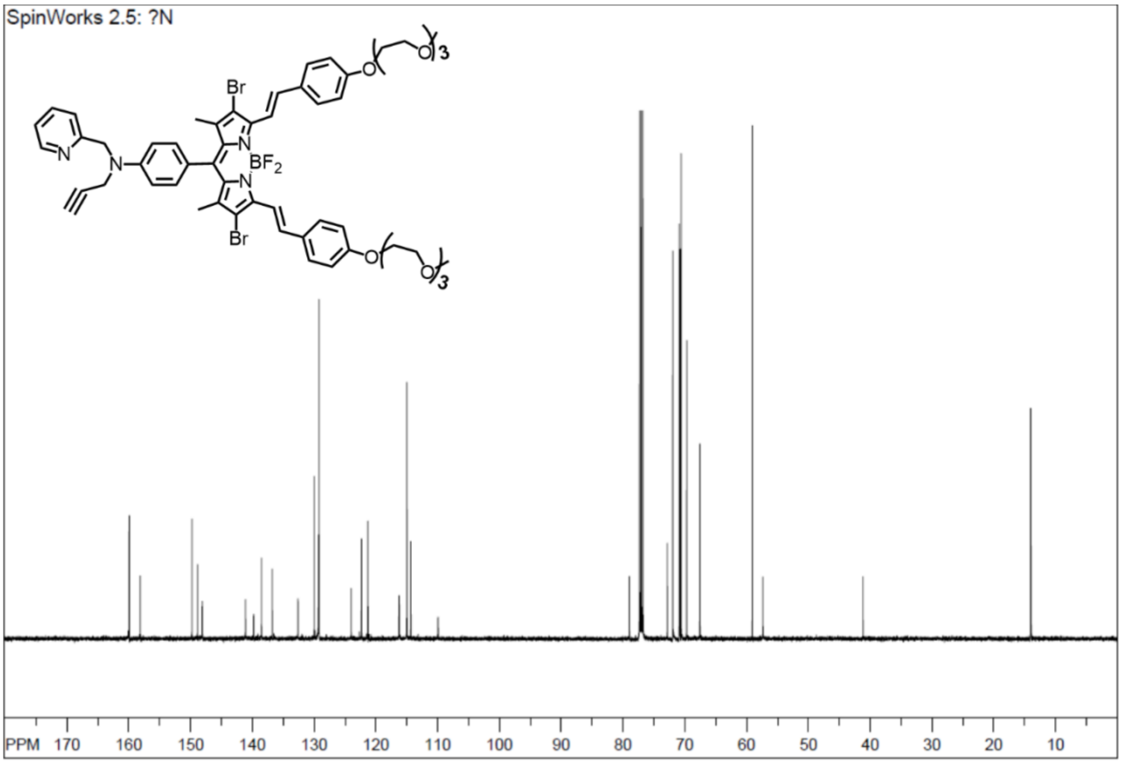

Figure S45. ^{13}C NMR spectrum (100 MHz) of **1** recorded in $CDCl_3$.

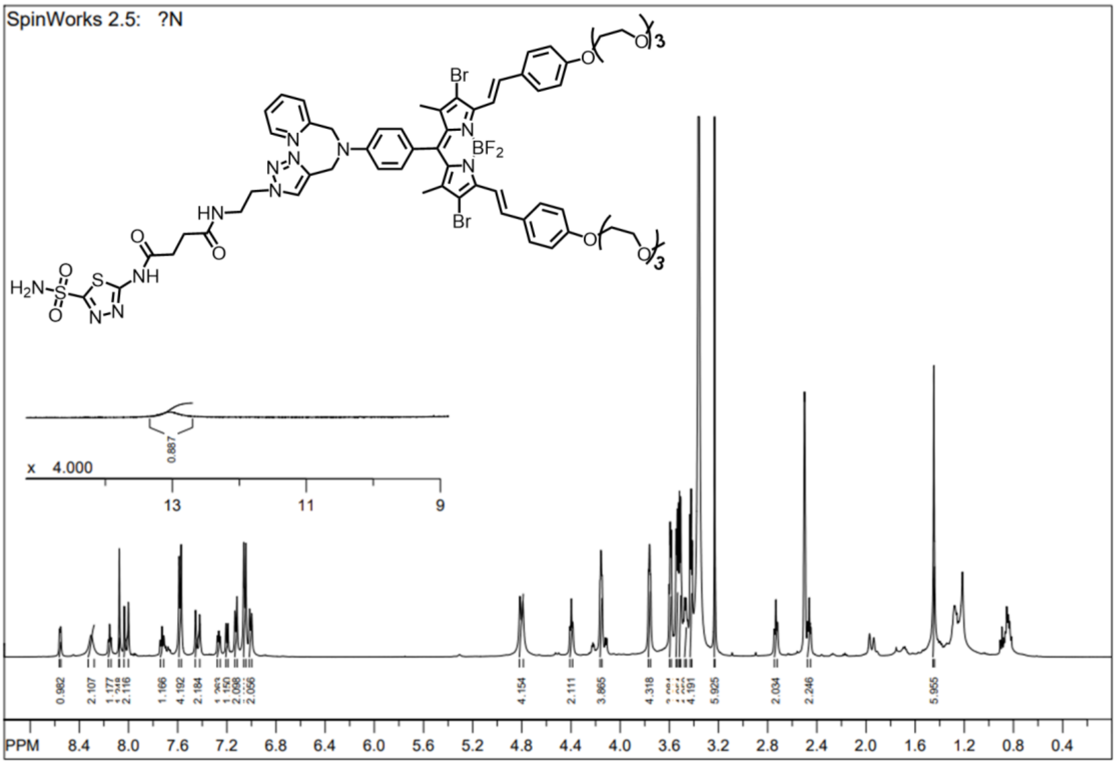

Figure S46. ^{1}H NMR spectrum (500 MHz) of **CA9-BPS** recorded in DMSO-*d*6.

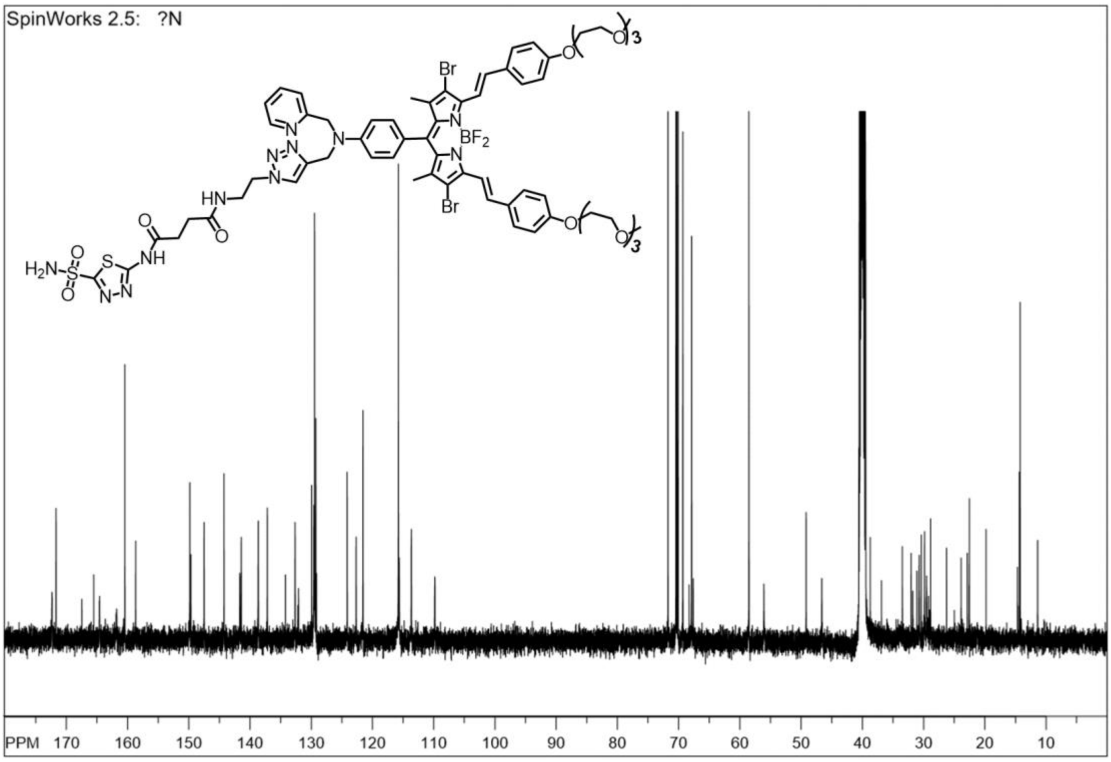

Figure S47. ^{13}C NMR spectrum (125 MHz) of **CA9-BPS** recorded in DMSO-*d*6.

30. ESI MS data

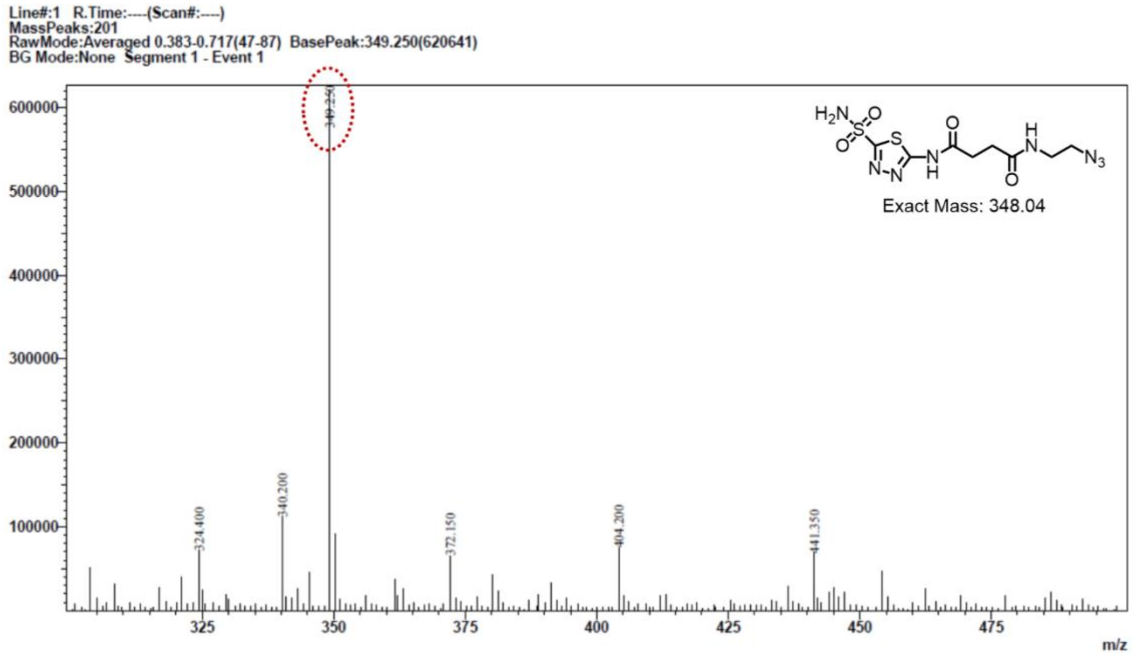

Figure S48. ESI-MS spectrum of **9**.

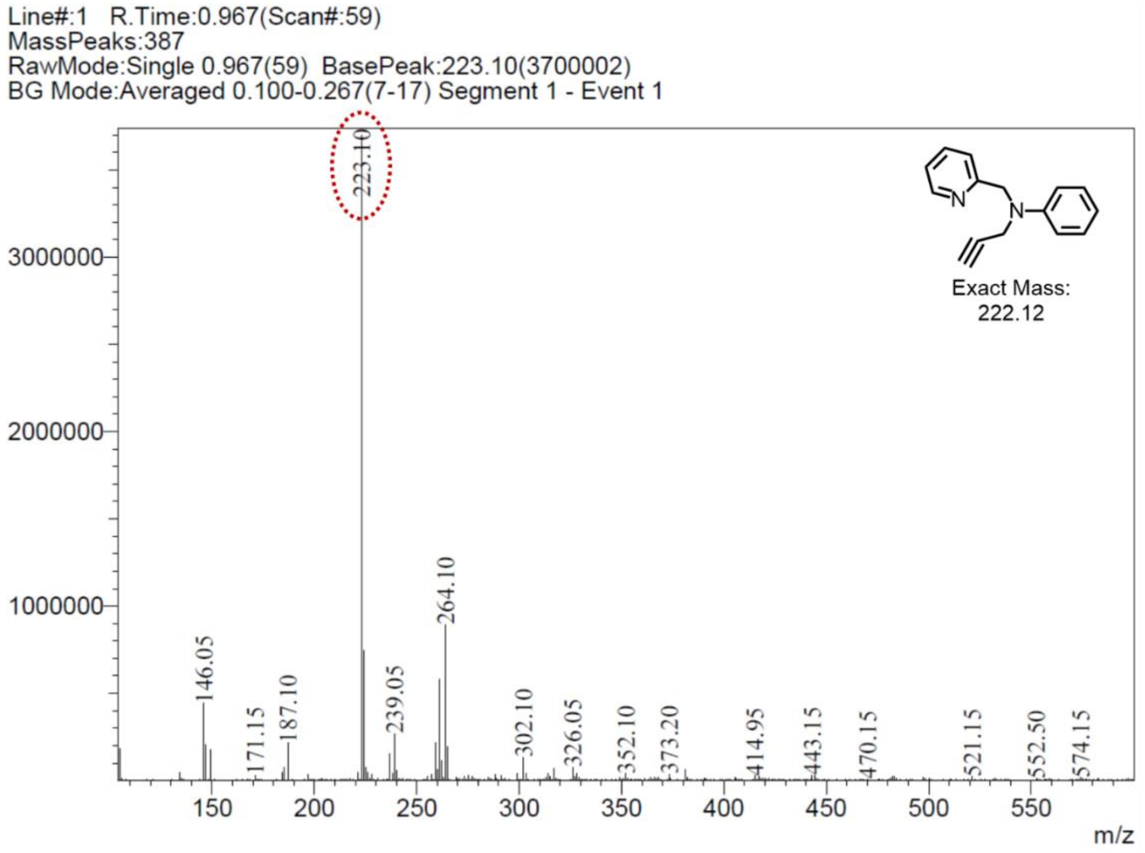

Figure S49. ESI-MS spectrum of **5**.

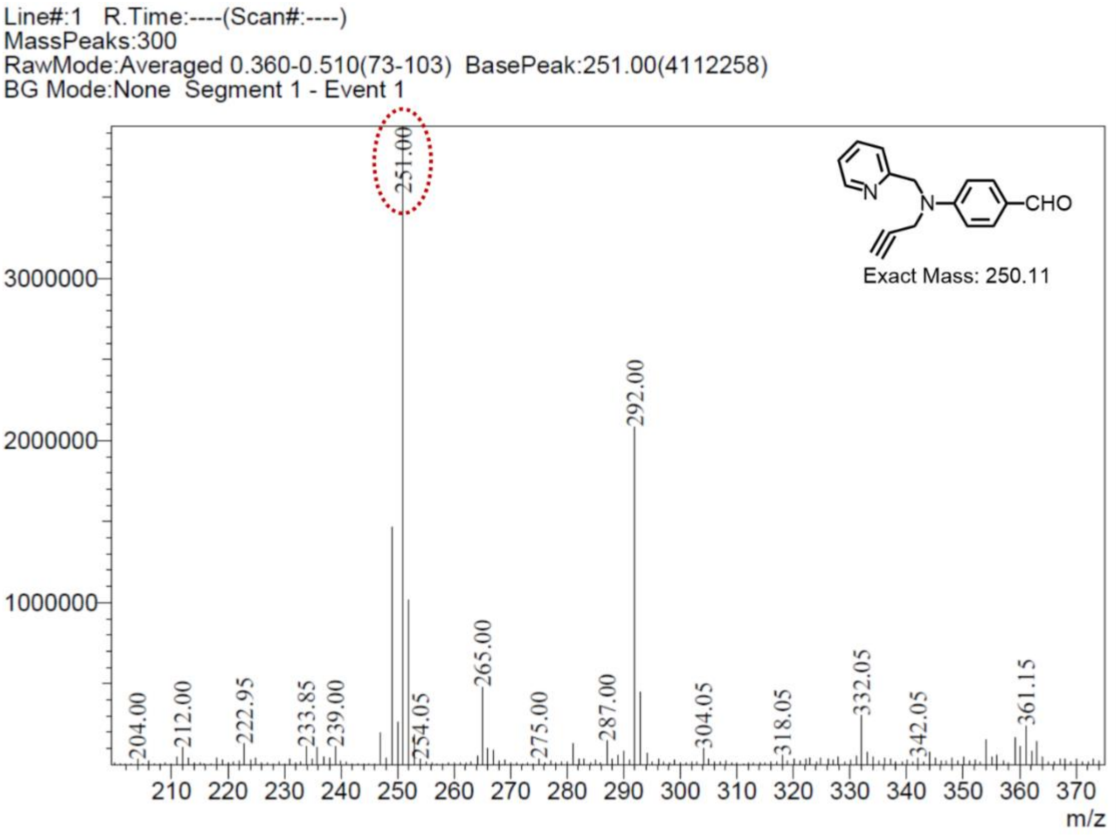

Figure S50. ESI-MS spectrum of **4**.

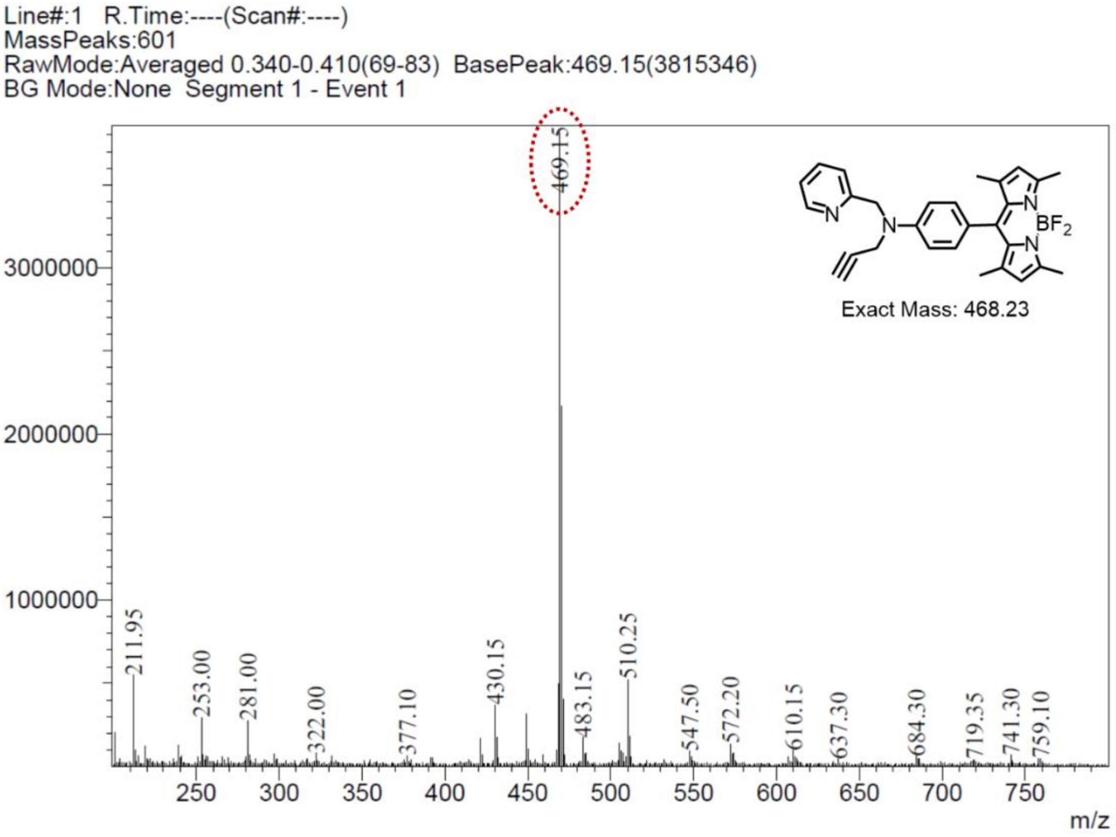

Figure S51. ESI-MS spectrum of **3**.

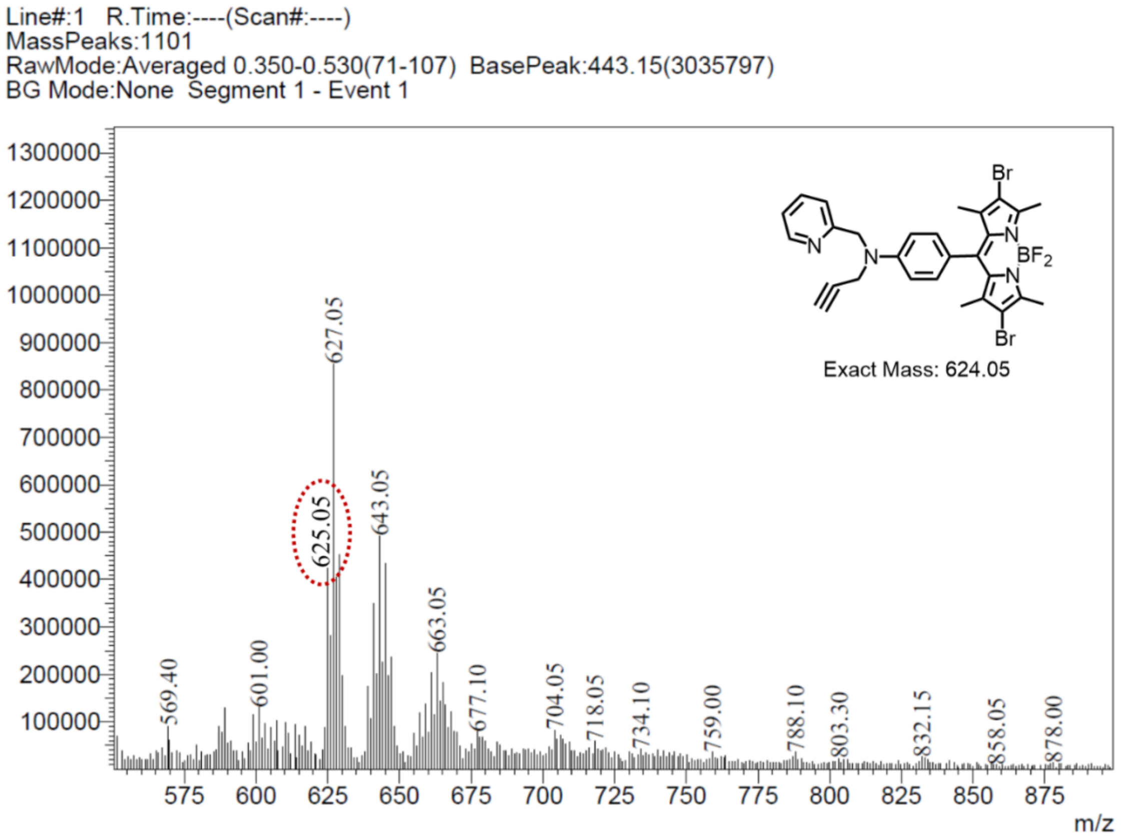

Figure S52. ESI-MS spectrum of **2**.

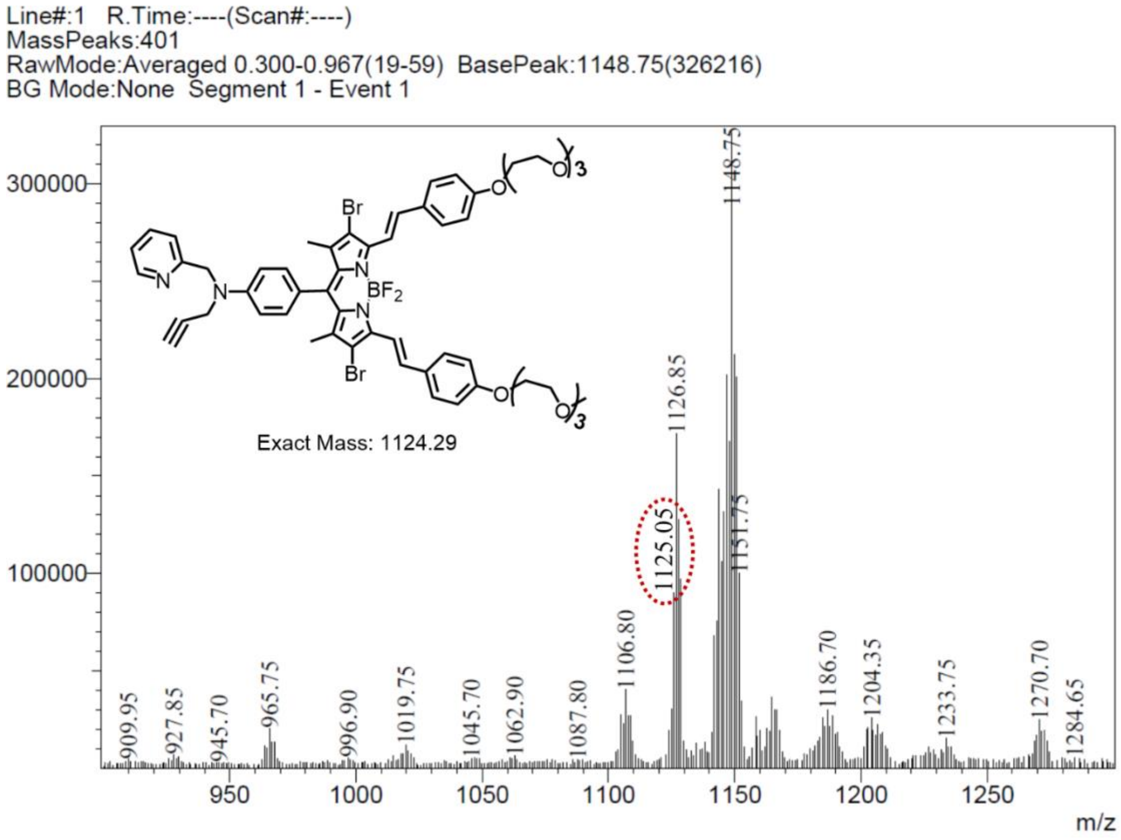

Figure S53. ESI-MS spectrum of **1**.

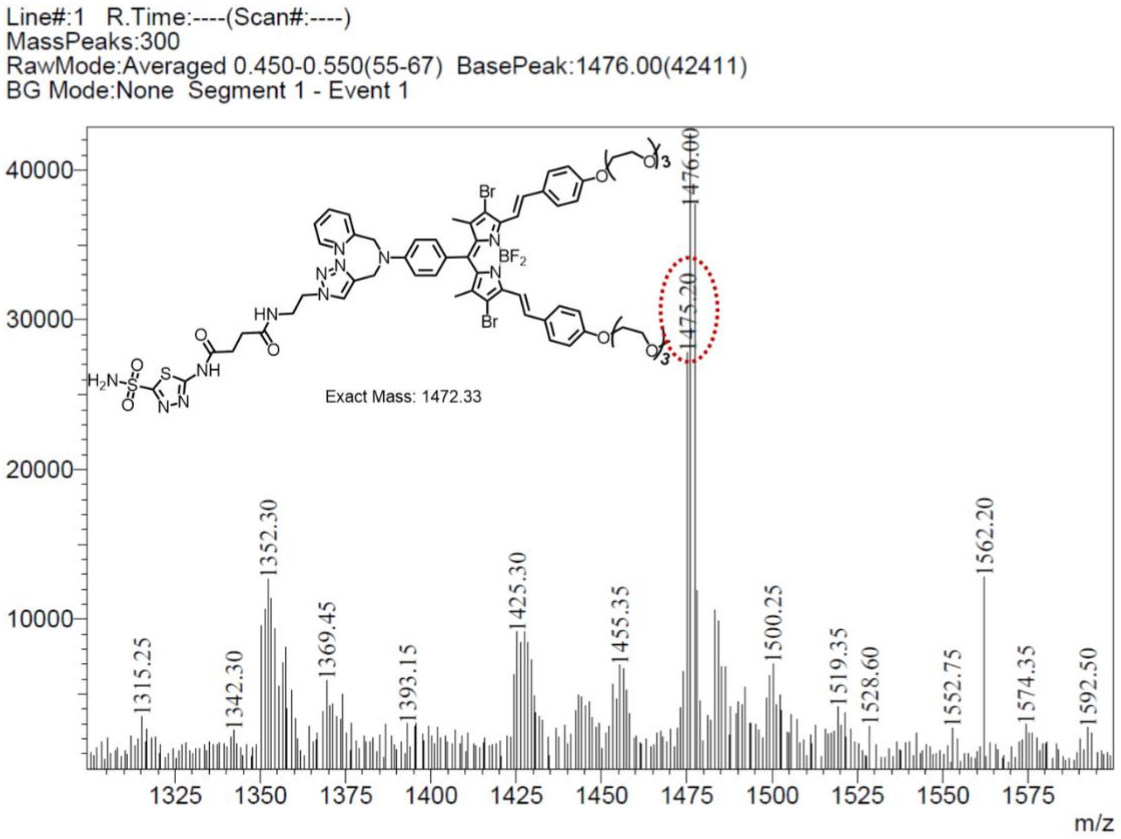

Figure S54. ESI-MS spectrum of **CA9-BPS**.

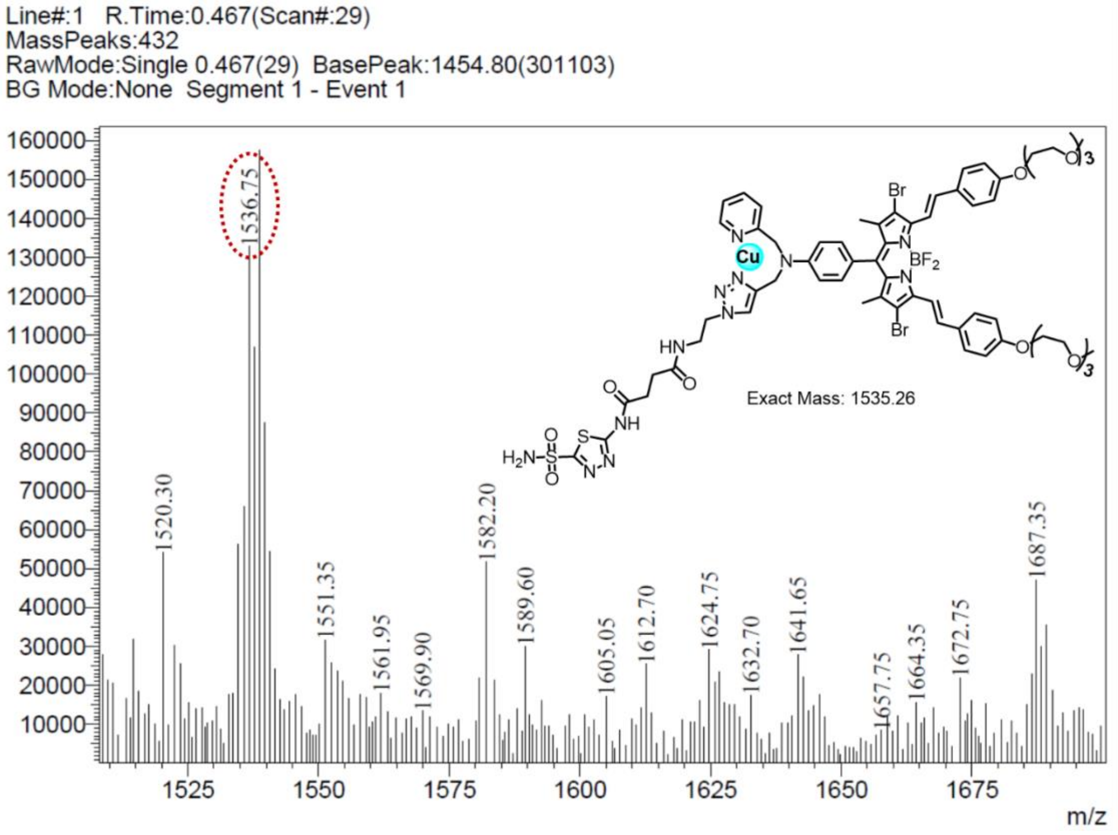

Figure S55. ESI-MS spectrum of **CA9-BPS-Cu(II)**.

31. Original western blot images

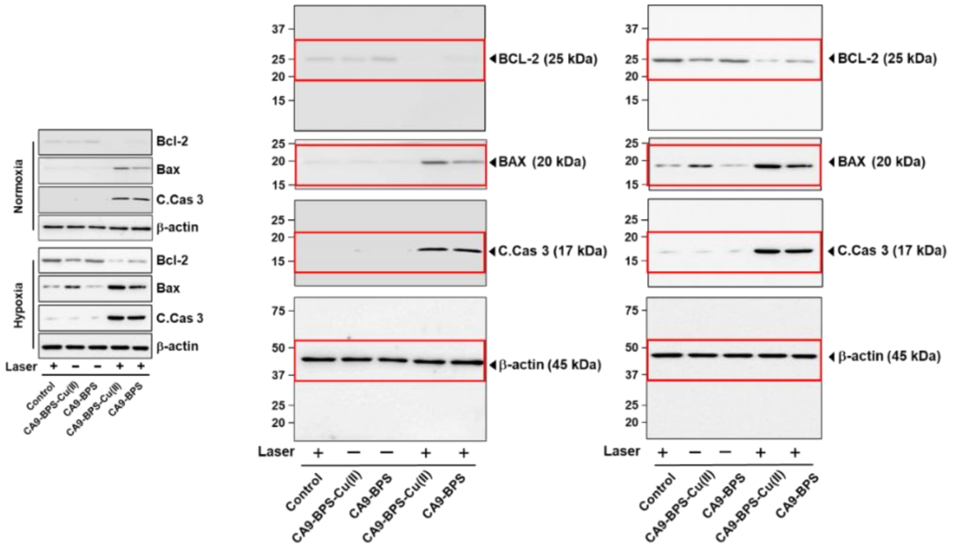

Figure S56. Original western blot images of cell death signaling proteins by **CA9-BPS** and **CA9-BPS-Cu(II)** in MDA-MB-231 cells for Fig. 3g.

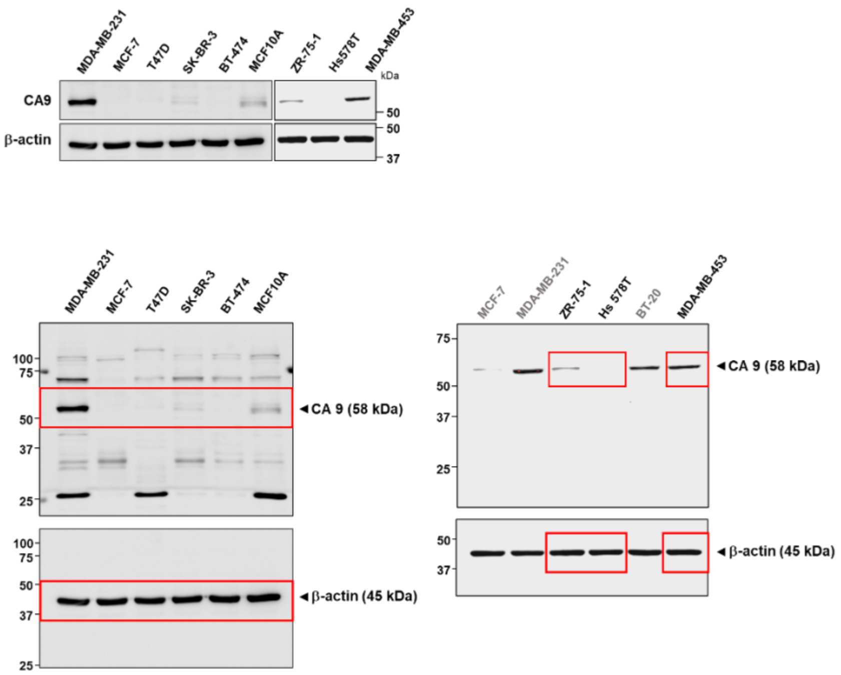

Figure S57. Original western blot images of CA9 expression in various breast cancer cells for Fig. S10.

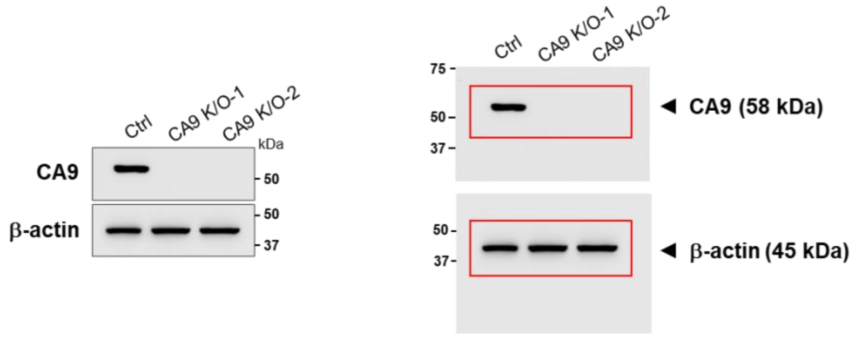

Figure S58. Original western blot images of CA9 expression in CA9 knockout MDA-MB-231 cells for Fig. S15.

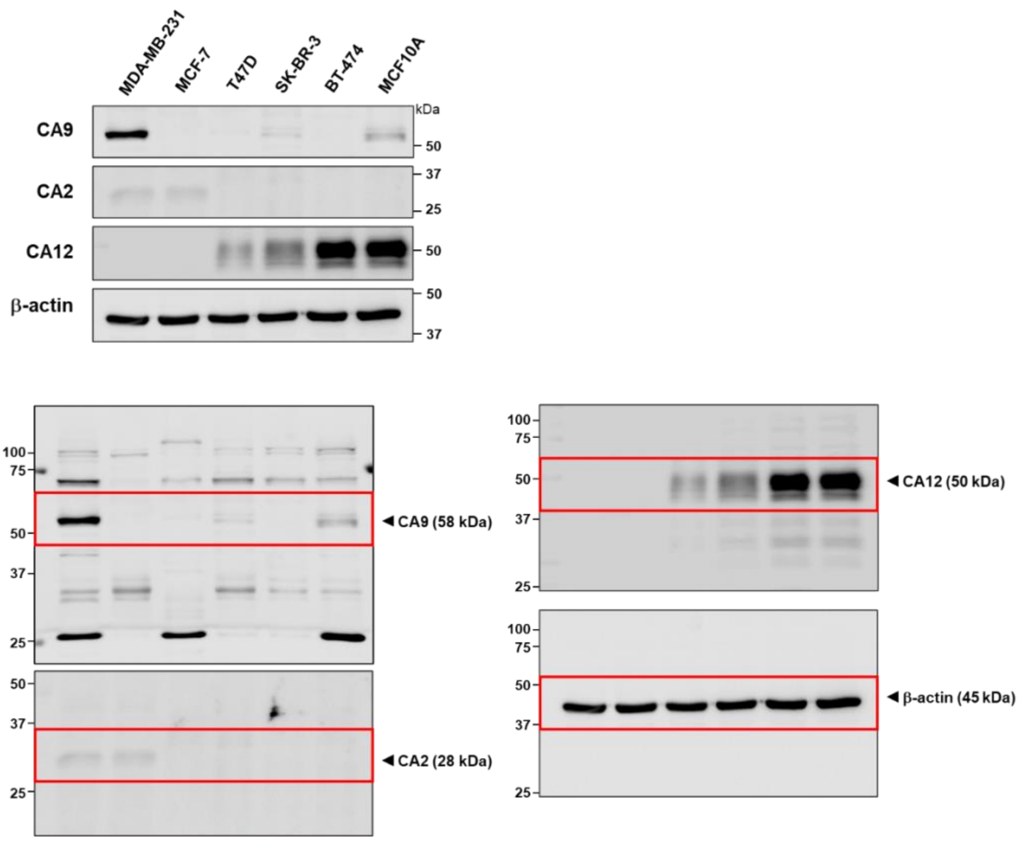

Figure S59. Original western blot images of CA isoforms in various breast cancer cells for Fig. S25.

32. References

1. J. K. J. Ahlskog, C. E. Dumelin, S. Trüssel, J. Mårlind and D. Neria, *Bioorg. Med. Chem. Lett.*, 2009, **19**, 4851–4856.
2. C. B. Nielsen, M. Johnsen, J. Arnbjerg, M. Pittelkow, S. P. McIlroy, P. R. Ogilby and M. Jørgensen, *J. Org. Chem.*, 2005, **70**, 7065–7079.
3. Y. Wang, H. Liu, X. Zhang, Z. Zhang and D. Huang, *Org. Biomol. Chem.*, 2017, **15**, 9164–9168.
4. P. S. Vincett, E. M. Voigt and K. E. Rieckhoff. *J. Phys. Chem.*, 1971, **55**, 4131–4140.
5. C. Campos, R. Guzmán, E. López-Fernández and A. Casado, *Anal. Biochem.*, 2009, **392**, 37–44.
6. M. Taki, S. Iyoshi, A. Ojida, I. Hamachi, and Y. Yamamoto, *J. Am. Chem. Soc.*, 2010, **132**, 5938–5939.
7. B. Ma, S. Wang, F. Liu, S. Zhang, J. Duan, Z. Li, Y. Kong, Y. Sang, H. Liu, W. Bu and L. Li, *J. Am. Chem. Soc.*, 2019, **141**, 849–857.
8. T.-C. Chou, *Cancer. Res.*, 2010, **70**, 2, 440–446.
